# Nature‐Inspired Compounds Targeting *Escherichia coli* WrbA as Biofilm‐Modulating Agents: Computational Design, Synthesis, and Biological Evaluation

**DOI:** 10.1002/ardp.70049

**Published:** 2025-07-11

**Authors:** Matteo Mori, Enrico Mario Alessandro Fassi, Federica Villa, Erica Ginevra Milano, Fabio Forlani, Francesca Cappitelli, Alessandro Ratti, Fiorella Meneghetti, Gabriella Roda, Giovanni Grazioso, Stefania Villa

**Affiliations:** ^1^ Department of Pharmaceutical Sciences University of Milan Milano Italy; ^2^ Department of Food, Environmental and Nutritional Sciences University of Milan Milano Italy

**Keywords:** antibiofilm assay, chroman‐4‐one derivatives, microscale thermophoresis, MM‐GBSA, reactive oxygen species

## Abstract

Biofilms pose significant challenges in multiple settings due to their resistance to conventional treatments. In this study, we designed and synthesized a novel class of nature‐inspired 5,7‐dihydroxy‐2,2‐dimethylchroman‐4‐one derivatives as binders of WrbA, a potential target for biofilm modulation. Using a structure‐based computational approach, a small library of analogs with varied amide moieties was developed and synthesized. The evaluation of their binding affinity to WrbA demonstrated good‐to‐excellent *K*_d_ values, as confirmed by microscale thermophoresis (MST). Antibiofilm assays against *Escherichia coli* and *Staphylococcus aureus* revealed different modulating effects on biofilm formation, conceivably linked to ROS production. These findings emphasize the importance of ROS levels in biofilm, as well as the pivotal role of WrbA as a target in its regulation.

## Graphical abstract

Nature‐inspired WrbA binders for biofilm modulation were designed and synthesized, utilizing computational methods to optimize and select key analogs. The resulting compounds exhibited varying effects on biofilm growth, with reactive oxygen species (ROS) levels likely playing a key role in regulating biofilm responses.

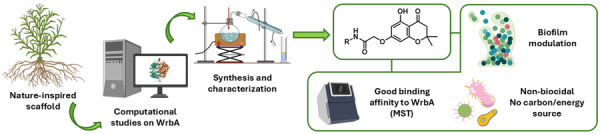

## 1 Introduction

Biofilms are structured microbial communities that develop on both biotic and abiotic surfaces in response to different environmental factors [1]. They consist of sessile cells enclosed within a self‐produced extracellular polymeric matrix (EPS), composed of polysaccharides, DNA, proteins, and other minor components [1]. This structure enables microbial cells to withstand adverse environmental conditions, including the presence of xenobiotics and antimicrobial agents [2, 3]. Hence, biofilms increase the virulence of pathogens and make them more resistant to conventional treatments, hindering effective eradication [2, 4]. In humans, biofilm‐associated conditions include cystic fibrosis (*Pseudomonas aeruginosa*), periodontitis (*Pseudomonas aerobicus*, and *Fusobacterium nucleatum*), otitis media (*Haemophilus influenzae*), infective endocarditis (*Staphylococcus aureus*, *Streptococcus viridans*, and *Enterococcus faecalis*), chronic wounds (*P. aeruginosa*), and osteomyelitis (*P. aeruginosa*) [5]. In the healthcare sector, exposed surfaces and medical devices (catheters, implants, etc.) provide ideal environments for the formation of biofilms [6, 7]. Besides the medical field, biofilms are also a cause of concern in many other areas, including water treatment, food, and maritime industries [6]. At the same time, it is worth mentioning that biofilms formed by nonpathogenic microorganisms can be neutral [8] or even beneficial in a variety of applications such as the production of bulk and fine chemicals, bioremediation, wastewater treatment, and agriculture [9, 10, 11].

In the pharmaceutical field, the primary strategy for combating biofilm predominantly involves the use of traditional antimicrobial agents. However, the current research focuses on the development of treatment approaches that hinder biofilm formation without affecting microbial life [12, 13, 14]. As a result, microorganisms are subjected to a lower selective pressure, preventing the development of drug‐resistant mutations [15]. This is especially important considering the alarming spread of antimicrobial resistance, a complex phenomenon heavily influenced by medical, social, cultural, and economic factors that is predicted to claim 10 million lives per year by 2050 [16, 17]. Recent research has suggested that oxidative stress is implicated in the shift from the planktonic state to the formation of biofilm [18, 19]. In this context, our team has contributed to the identification of a connection between the function of the tryptophan [W] repressor‐binding protein (WrbA) and biofilm [19]. WrbA was first discovered in *Escherichia coli*, but it is found in a wide range of cell types across all three domains of life [20]. This protein is a quinone oxidoreductase that influences biofilm formation via a reactive oxygen species (ROS)‐dependent mechanism [19]. The loss of WrbA function has been associated with a ROS‐sensitive phenotype, marked by decreased biofilm cell density, reduced biofilm thickness, lower EPS polysaccharide levels, and diminished tolerance to hydrogen peroxide (H_2_O_2_) [19]. Our group has also identified several natural compounds exhibiting antibiofilm activities at sublethal concentrations by a mechanism linked to oxidative stress. One of these derivatives, namely zosteric acid, has been shown to directly interact with WrbA as its primary target in a pull‐down assay [21]. Furthermore, cinnamic acid and salicylic acid have shown similar antibiofilm effects, related to the interaction with this protein [21, 22]. More recently, our team has also proposed a WrbA‐mediated mechanism for the antibiofilm action of ellagic acid [23] and disclosed a new group of natural products, including fisetin, morin, purpurogallin, and NZ034, in virtual screening campaigns on WrbA [24]. These compounds were capable of modulating cell redox homeostasis, resulting in the inhibition of biofilm formation at nonlethal concentrations. The correlation between the effect of these molecules and WrbA was especially evident for ellagic acid, purpurogallin, and NZ034, as confirmed by observations on a WrbA‐deleted *E. coli* mutant [23, 24]. At the same time, it is essential to acknowledge that WrbA might represent merely one among the possible molecular targets for these natural compounds, as demonstrated by fisetin and morin [24].

In this work, we built upon our previous studies to develop a series of specific synthetic WrbA binders as potential nontoxic biofilm‐modulating agents. A visual inspection of the most promising hit molecules selected by our computational method among a database of natural products revealed the recurrence of the 5,7‐dihydroxy‐2,2‐dimethylchroman‐4‐one core [24]. This observation, combined with the knowledge that 4‐chromanone makes up the core of several bioactive compounds [25, 26], encouraged us to further examine this nucleus to obtain novel WrbA ligands capable of altering bacterial biofilm. With this aim, we started from our WrbA model to develop a computational protocol for the design of new compounds, which were selected based on their docking score, predicted stability in the binding site, and synthetic feasibility. The best candidates were synthesized, characterized, and tested for their ability to hinder biofilm formation in *E. coli* and *S. aureus*, two medically relevant pathogens. Our results led to the identification of promising, non‐bactericidal biofilm‐modulating candidates, further emphasizing the potential of targeting WrbA for the development of agents that operate by a redox‐dependent mechanism to influence biofilm formation.

## 2 Results and Discussion

### 2.1 Rational Design of Compounds

**Figure 1:**
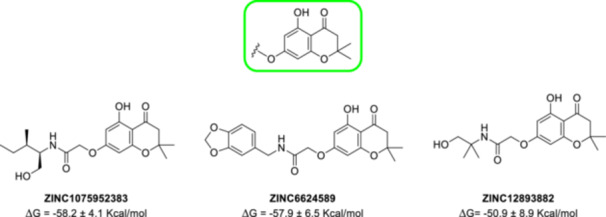
Structure of the recurrent 5,7‐dihydroxy‐2,2‐dimethylchroman‐4‐one moiety (in the green square) and three examples of natural compounds selected by the computational protocol described in our previous work [24].

In our previous paper, we reported on the identification of natural compounds displaying antibiofilm properties linked to WrbA inhibition [24]. Interestingly, the visual inspection of the top‐ranked compounds resulting from our virtual screening study revealed that the 5,7‐dihydroxy‐2,2‐dimethylchroman‐4‐one moiety was a recurring feature (Figure 1). Considering the relevance of chromanones in medicinal chemistry [25, 26], as well as their synthetic accessibility, we selected this scaffold as a starting point for the design of WrbA binders, with the aim of obtaining new compounds exhibiting biofilm‐modulating properties.

Therefore, adopting a computational protocol constituted by docking, molecular dynamics (MD), and molecular mechanics–generalized born surface area (MM‐GBSA) calculations (see Section 4 for details), the prototypal Compounds **A**–**C**, reported in Table 1, were simulated in a complex with WrbA.

**Table 1:** Predicted binding free energy values for the 5,7‐dihydroxy‐2,2‐dimethylchroman‐4‐one analogs **A**–**C**.

Entry	Structure	Δ*G* a ± SE of mean (kcal/mol)
**A**	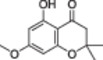	−41.0 ± 3.1
**B**	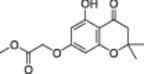	−47.0 ± 3.4
**C**	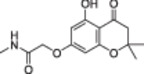	−50.1 ± 5.2

^a^ See Section 4 for details.

**Figure 2:**
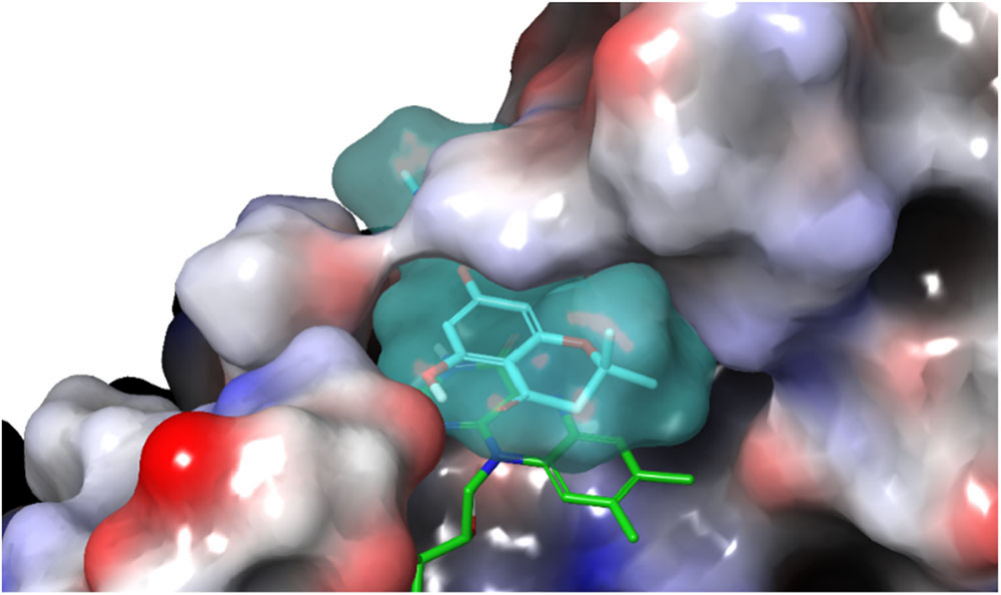
Compound **C** (light‐blue surface) in complex with WrbA. The flavin mononucleotide (FMN) cofactor is displayed as green sticks. The protein is represented as a molecular surface, color‐mapped based on the electrostatic potential (blue to red for positively to negatively charged areas, respectively).

The visual examination of the MD trajectory frames, along with the analysis of the RMSD/time plot of the ligand heavy atoms, revealed that Compound **A** remained stable only during the initial phase of the simulation, subsequently dissociating from the catalytic site. Conversely, Compound **B** displayed a remarkable stability for the entire duration of the simulation, thanks to (1) the hydrogen bond established between the ligand carbonyl group and the side chain of WrbA‐His132 and (2) the π–π stacking between the ligand ring and the side chain of WrbA‐Trp98. Furthermore, the presence of the amide group in Compound **C**, replacing the ester moiety of **B**, led to an additional interaction with WrbA‐Thr115 (Figure 2).

From these observations, Compound **C** emerged as the most promising candidate. Consequently, it was selected as the core scaffold for the design of new molecules, suitably decorated to achieve the highest predicted affinity on WrbA. Next, a structure‐based virtual screening study was carried out, aimed at identifying the best amide substituents to form optimal interactions with the WrbA binding site. In these calculations, a library of 25k compounds was generated by the “Reaction‐Based Enumeration” tool of Maestro (release 2021‐2, Schrödinger LLC, New York, NY, USA). With this method, a specific molecule (in this case, the acid derivative of **C**) can be coupled with a library of reactants (commercially available amines) to generate a collection of products (amides). Then, by applying four filters aimed at reducing the number of generated compounds and increasing the synthetic feasibility (see Section 4 for details), a final library of compounds was docked in the catalytic site of WrbA. The most promising hits (about 50) were simulated in complex with the target by 150‐ns‐long MD simulations, and the MM‐GBSA method was used to estimate their binding free energy values. Ultimately, the four best compounds overall (**1b–e**, Table 2) were selected to be synthesized and evaluated. To these, the unsubstituted amide **1a** was added to act as a negative control and proof‐of‐concept of our design approach.

**Table 2:** List of the compounds selected by our computational protocol, with their predicted Δ*G* values.

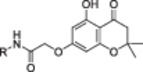		
Entry	R group	Δ*G* a ± SE of mean (kcal/mol)
**1a**	H	−53.0 ± 4.2
**1b**	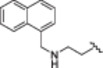	−75.0 ± 6.6
**1c**	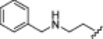	−69.6 ± 6.0
**1d**	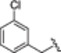	−67.6 ± 8.6
**1e**	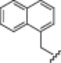	−66.7 ± 3.0

^a^ See Section 4 for details.

**Figure 3:**
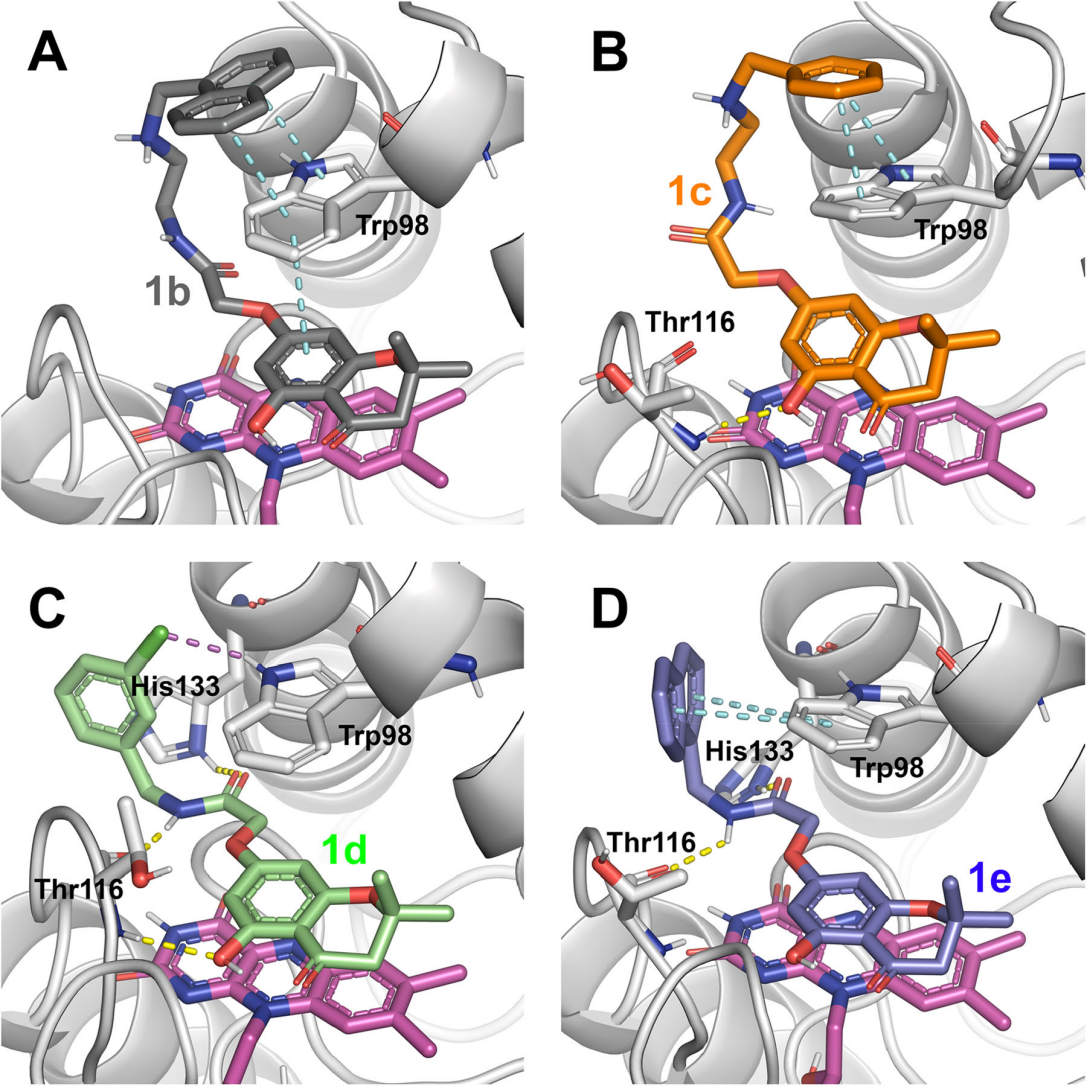
Predicted binding mode for Compounds **1b–e** (panels A–D, respectively) in complex with WrbA, resulting from the MD frames showing the lowest calculated binding free energy values. The secondary structure of WrbA is represented as gray ribbons with some key residues (Trp98, Thr116, and His133) shown as sticks, while FMN is represented as magenta sticks. Hydrogen bonds are represented as yellow dashed lines, π–π stacking as cyan dashed lines, and halogen bonds as purple dashed lines.

The analysis of the MD trajectory frames revealed that all selected compounds (**1b–e**) were firmly bound in the catalytic site of the enzyme over the whole simulation time. The R groups of **1b** and **1c** formed sandwich π–π stacking with the indole ring of WrbA‐Trp98, conferring high stability during the MD calculations. In a similar way, the naphthalene group of **1e** established a π–π bond with the same residue. This pose was additionally stabilized by a hydrogen bond network between the nitrogen and oxygen atoms of the amide group and WrbA‐His132. Finally, the R substituent in **1d** allowed the formation of a hydrogen bond with WrbA‐Trp97 and a π–π stacking with WrbA‐His132. A depiction of the predicted interaction network for **1b–e** is provided in Figure 3.

### 2.2 Chemistry

**Scheme 1:**
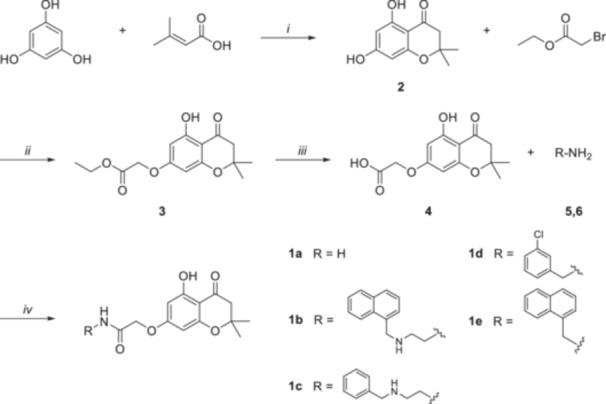
Reagents and conditions: (i) PPA, 1,4‐dioxane, 60°C, 3 h, N_2_ atm; (ii) K_2_CO_3_, CH_3_CN, 0°C, 5 h, N_2_ atm; (iii) 2.5 M NaOH, EtOH/H_2_O, 0°C, 0.5 h; (iv) *1*. HATU, DIPEA, DMF, RT, 45 min, N_2_ atm; *2*. R‐NH_2_, RT, overnight, N_2_ atm.

The 5,7‐dihydroxy‐2,2‐dimethylchroman‐4‐one scaffold was obtained by a cyclodehydration reaction between phloroglucinol and 3‐methylbutenoic acid in polyphosphoric acid (PPA). The latter was chosen as a greener and safer alternative to POCl_3_ to achieve the cyclized product. The resulting intermediate (**2**) was reacted with ethyl 2‐bromoacetate in the presence of K_2_CO_3_ to afford Compound **3**. After the hydrolysis of the ester function, the corresponding acid (**4**) was reacted with the appropriate amine in the presence of HATU and DIPEA to give amides **1a–e**. For the synthesis of **1a**, **1c**, and **1d**, commercially available amines were used, whereas **1b** and **1e** were obtained from previously synthesized intermediates (**5** and **6**, respectively). In detail, for intermediate **5**, 1‐(chloromethyl)naphthalene was reacted with *N*‐Boc‐ethane‐1,2‐diamine in a nucleophilic substitution promoted by TEA to afford Compound **7**, which was N*‐*deprotected with TFA in DCM to yield the desired amine (**5**). Naphthalen‐1‐ylmethanamine (**6**) was obtained by the Gabriel synthesis starting from 1‐(chloromethyl)naphthalene, which was reacted with potassium phthalimide in DMF to afford the corresponding N‐substituted phthalimide. The amine (**6**) was recovered by the Ing–Manske procedure, using hydrazine hydrate in EtOH at reflux. Further details regarding synthetic procedures are reported in Section 4 and Supporting Information S2. Scheme 1 describes the main steps of the synthetic procedure for the obtainment of **1a–e**.

### 2.3 Crystallography

**Figure 4:**
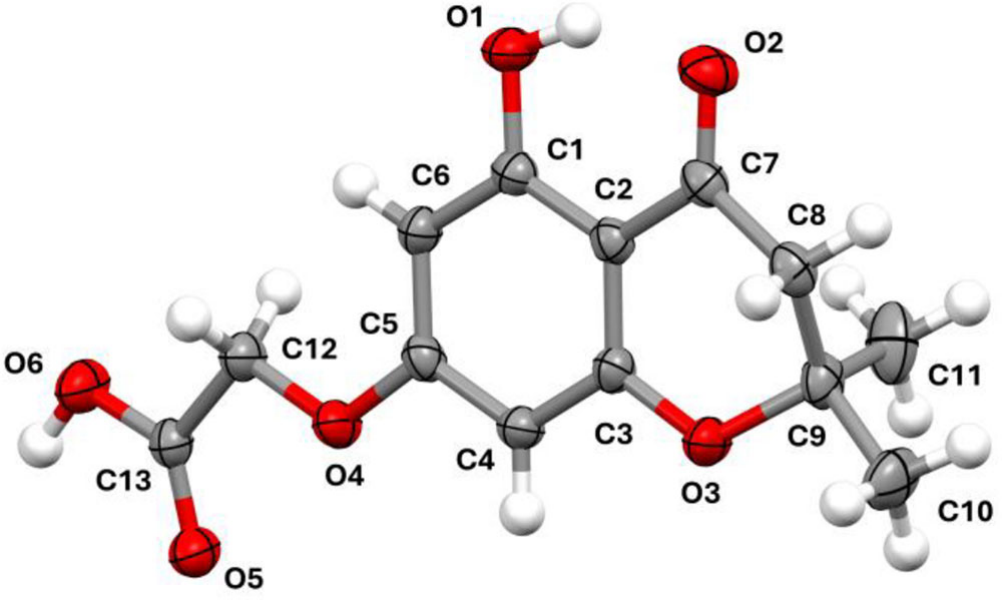
Thermal ellipsoid diagram of **4**, with the arbitrary atom‐numbering scheme used in the discussion. Ellipsoids represent atomic displacement parameters at the 40% probability level.

Compound **4** crystallized in the triclinic space group P−1, with a single molecule in the asymmetric unit. Its molecular structure is presented in Figure 4 as an ellipsoid diagram, with an arbitrary atom‐numbering scheme.

**Figure 5:**
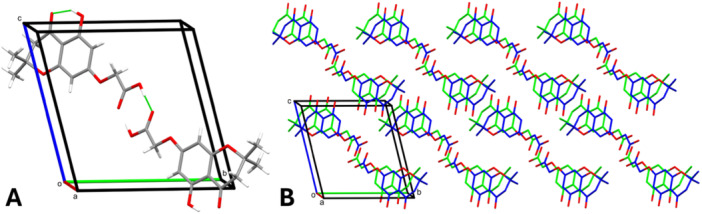
(A) Representation of the intermolecular and intramolecular H bonds (green lines). Symmetry‐equivalent interactions are not shown. (B) Representation of the crystal packing, viewed along the *a* axis. Hydrogen atoms have been omitted for clarity.

The molecule is characterized by a substituted 4‐chromanone ring. The aromatic portion is planar, while the pyrone ring is in a slightly distorted half‐chair conformation (Cremer–Pople puckering parameters: *θ* = 123.00°, *φ* = 18.02°, and *Q*_T_ = 0.4538) [27]. The maximum deviation from the best mean plane of the pyrone is 0.312 Å, corresponding to the position of C9. The acidic side chain is bent with respect to the aromatic ring, forming an angle of 9.29° (planes were calculated using the positions of C1–C2–C3–C4–C5–C6 and O4–C12–C13–O6, respectively). The crystal packing (Figure 5) is stabilized by H bonds between the acidic moieties of two adjacent molecules related by an inversion center (D–H = 0.936(33) Å, H⋯A = 1.708(33) Å, D⋯A = 2.633(2) Å, and D–H⋯A = 168(2)°). Despite the presence of an aromatic ring, there is no π–π stacking; instead, weak hydrophobic contacts complement the interaction network of the molecules. Finally, the hydroxyl group forms an intramolecular H bond with the carbonyl oxygen of the pyrone ring (D–H = 0.929(32) Å, H⋯A = 1.783(28) Å, D⋯A = 2.613(2) Å, and D–H⋯A = 147(2)°).

**Figure 6:**
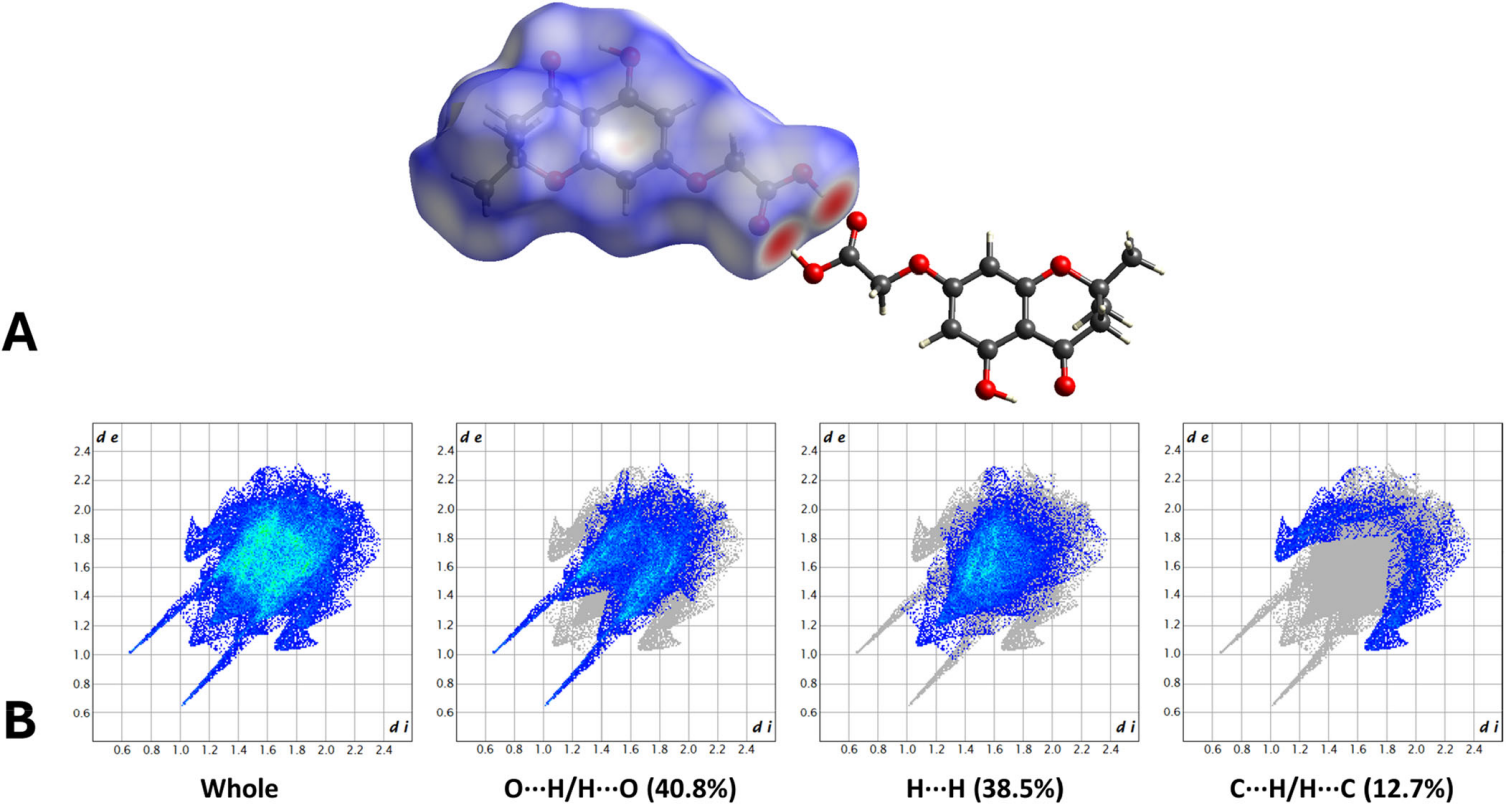
(A) HS mapped over *d*_norm_ with a fixed color scale from −0.7373 au (red) to 1.1305 au (blue), representing intermolecular contact length relative to the sum of the van der Waals radii (red: shorter; blue: longer; white: equivalent). (B) Two‐dimensional fingerprint plots of the HS, illustrating the frequency of each *d*_*e*_ and *d*_*i*_ combination. Points are color‐coded from blue to green based on their contribution.

The intermolecular contacts were also inspected by generating the Hirshfeld surface (HS; *V* = 300.91 Å^3^, *A* = 283.63 Å^2^, *G* = 0.766, and Ω = 0.255), visualized as a function of the normalized contact distance (*d*_norm_; Figure 6A). The analysis revealed the presence of intense red regions, corresponding to the H bonds bridging the acidic moieties of two adjacent molecules. These strong, short‐range interactions were also examined via the 2D fingerprint plot (Figure 6B), which showed two elongated spikes extending towards the lower left corner of the graph. The importance of O⋯H/H⋯O contacts was confirmed by their large surface area (40.8%) and by the calculation of their enrichment ratio (1.2) [28]. The remaining portions of the HS represented weak, nonspecific Van der Waals interactions, displayed by white‐to‐blue areas on the surface (Figure 6B). Further structural details are available in Supporting Information S2.

### 2.4 Biophysical Investigations

**Figure 7:**
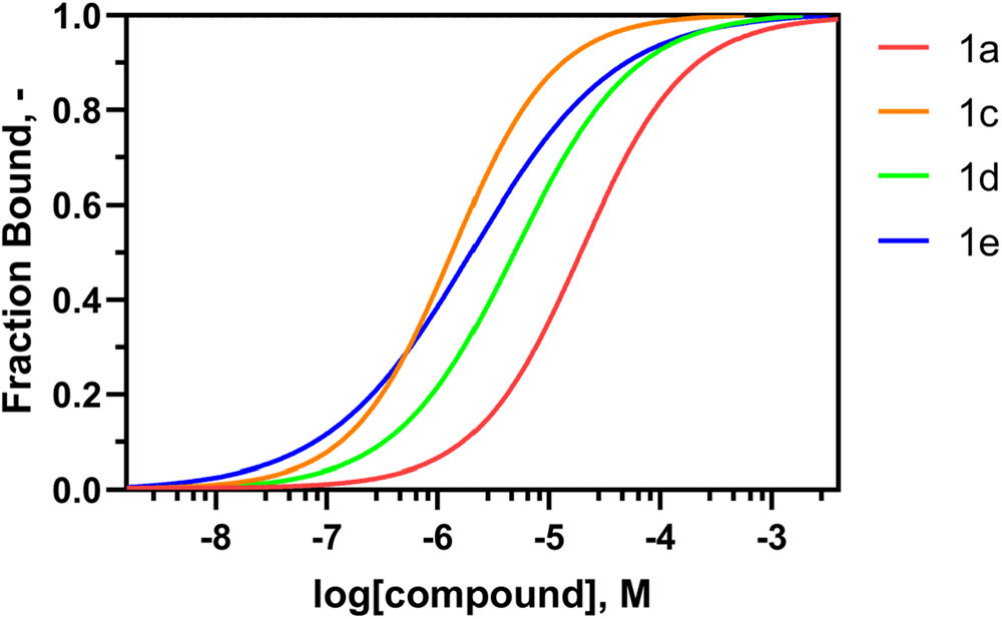
MST curves normalized by fraction bound acquired by incubation of recombinant WrbA protein with scaling concentrations of Compounds **1a** (red), **1c** (orange), **1d** (green), and **1e** (blue), using a Monolith NT.115 instrument. Two independent experiments were conducted to generate the *K*_d_ curve.

Microscale thermophoresis (MST) experiments were performed to measure the *K*_d_ values of the synthesized compounds on the recombinant WrbA protein (see Section 4 for details). Initially, the *K*_d_ of **1a** was determined to obtain a reference value to be compared to those of the amide derivatives **1b–e**. In these measurements, **1a** exhibited a *K*_d_ of 18.7 ± 6.3 μM, **1c** a *K*_d_ of 1.4 ± 0.7 μM, **1d** a *K*_d_ of 5.0 ± 1.8 μM, and **1e** a *K*_d_ of 2.1 ± 1.3 μM (Figure 7, Table 3). Concerning **1b**, the poor solubility of the compound in the assay buffer prevented an unambiguous determination of the binding curve and *K*_d_ value. The detailed MST *K*_d_ curves for each compound and the summary table of the experimental conditions applied in the MST assays are available in Supporting Information S2: Figure 27 and Table 4, respectively.

**Table 3:** Results of the MST measurements on the amide derivatives **1a** and **1c–e**, calculated on a Monolith NT.115 instrument. Compound **1b** was not assayed due to its poor solubility in the test conditions.

Entry	*K*_d_ ± confidence (μM)
Ellagic acid	1.5 ± 0.4^a^
**1a**	18.7 ± 6.3
**1c**	1.4 ± 0.7
**1d**	5.0 ± 1.8
**1e**	2.1 ± 1.3

^a^ Data retrieved from reference [23].

In summary, all the amide derivatives tested with this technique (**1c–e**) afforded *K*_d_ values lower than that of the negative control **1a** (Table 3). Hence, the substitution of the amide was confirmed to be pivotal in increasing the affinity of the compounds, as predicted by the computational simulations (Table 2). Our results also suggested that the presence of an aromatic group, such as a benzene (**1c**) or a naphthalene (**1e**), incorporated either into a long or short chain (as in the cases of **1c** and **1e**, respectively), enhances the affinity toward the WrbA protein. This improvement is likely attributable to the formation of π–π stacking interactions with the indole ring of WrbA‐Trp98, as observed in the best poses derived from MD simulations (Figure 3B–D), further supporting the accuracy of the computational predictions.

### 2.5 Biological Assays

Before assessing the biofilm‐modulating potential of each compound, their ability to function as carbon and energy sources, as well as their effects on the planktonic growth of *E. coli* and *S. aureus*, was evaluated. The results indicated that none of the selected compounds could serve as the only carbon and energy source, as no bacterial growth was observed when tested at 500 μM in phosphate‐buffered saline (PBS).

**Figure 8:**
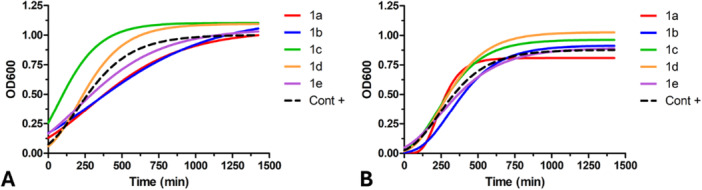
Planktonic growth of *E. coli* (A) and *S. aureus* (B) in the presence or absence (control) of 500 μM of each compound. Data are expressed as the mean of three independent measurements.

Additional experiments were performed in nutrient‐rich media, both in the presence and absence of 500 μM of each compound, to assess their impact on the planktonic growth of bacteria. The aim was to ensure that any potential antibiofilm effects of the selected molecules were not the result of biocidal action but rather a specific interaction with biofilm formation processes, leaving bacterial viability unaffected. The growth curves and kinetic parameters, shown in Figure 8 and Table 4, revealed that the maximum growth rates were not negatively affected by the presence of the compounds. These results confirmed that all the tested compounds, up to 500 μM, had no detrimental impact on the planktonic growth of *S. aureus* and *E. coli* and displayed no biocidal effects at these concentrations.

**Table 4:** The maximum growth rate was calculated using the Gompertz model applied to the planktonic growth curves of *E. coli* and *S. aureus*, both in the presence or absence of 500 μM of each compound. The goodness of fit (*R* ^2^) was also assessed to evaluate the quality of the Gompertz models. Data presented in the table are the mean values ± SDs obtained from independent measurements. No statistically significant differences between conditions were observed, as determined by Tukey's honestly significant difference (HSD) test, with a significance threshold of *p* ≤ 0.05.

		**1a**	**1b**	**1c**	**1d**	**1e**	Control
*E. coli*	Max growth rate (*A*₆₀₀; min)	1.22 × 10^−3^ ± 1.42 × 10^−4^	1.13 × 10^−3^ ± 1.41 × 10^−4^	2.48 × 10^−3^ ± 7.00 × 10^−4^	2.23 × 10^−3^ ± 3.88 × 10^−4^	1.20 × 10^−3^ ± 8.11 × 10^−4^	1.73 × 10^−3^ ± 4.18 × 10^−4^
	*R* ^2^	0.975	0.972	0.995	0.976	0.982	0.977
*S. aureus*	Max growth rate (*A*₆₀₀; min)	2.22 × 10^−3^ ± 2.65 × 10^−4^	1.93 × 10^−3^ ± 8.65 × 10^−5^	2.04 × 10^−3^ ± 3.26 × 10^−5^	2.16 × 10^−3^ ± 6.04 × 10^−5^	1.51 × 10^−3^ ± 3.72 × 10^−4^	1.88 × 10^−3^ ± 6.33 × 10^−4^
	*R* ^2^	0.990	0.984	0.960	0.968	0.981	0.972

The results of the adhesion assay in Table 5 showed that Compounds **1a**–**1c** had weak antibiofilm activities against both bacterial strains. Instead, these molecules increased the number of adhered cells at some concentrations, indicating possible biofilm stimulation. Compounds belonging to this class could act as prooxidants at sublethal concentrations, disrupting the normal redox cycle and leading to ROS accumulation by inhibiting the NADH:quinone oxidoreductase enzyme WrbA [19, 21]. The generated low levels of ROS may function as signaling molecules that can reduce or increase cell attachment and biofilm formation [18, 19]. Although high levels of ROS can induce cell death, lower concentrations are increasingly recognized for their critical role as signaling molecules with regulatory functions [18, 29, 30, 31]. For example, in *Candida albicans* biofilms, ROS are involved in signaling through the quorum‐sensing molecule farnesol, which significantly impacts biofilm formation [32]. Within bacterial biofilms, ROS promote genetic variability and regulate biofilm development [33]. Additionally, sublethal doses of hydrogen peroxide are essential in selecting pro‐biofilm‐forming pathogenic variants by modulating cyclic‐di‐GMP levels in *P. aeruginosa* [34]. Thus, ROS show a level‐dependent behavior, promoting biofilm formation at higher levels and reducing it at lower levels.

**Table 5:** Results of the antibiofilm assays on the synthesized compounds (**1a–e**). The table reports the number of adhered cells (mean values ± SDs) and the antibiofilm effect (%) of the compounds at different nonlethal concentrations against *S. aureus* and *E. coli*. The antibiofilm effect was quantified by calculating percentage values relative to the corresponding control samples. Negative values reflect a reduction in cell adhesion, whereas positive values indicate an enhancement.

		*Staphylococcus aureus*		*Escherichia coli*	
Compound	Concentration (µM)	Number of adhered cells	Antibiofilm effect (%)	Number of adhered cells	Antibiofilm effect (%)
**1a**	500	2.53 × 10^6^ ± 2.64 × 10^5^	−47	5.85 × 10^6^ ± 4.39 × 10^5^	−51
	50	2.15 × 10^6^ ± 2.91 × 10^5^	−25	5.52 × 10^6^ ± 6.15 × 10^4^	−43
	5	2.58 × 10^6^ ± 4.64 × 10^5^	−50	5.03 × 10^6^ ± 1.90 × 10^5^	−30
	0.5	1.61 × 10^6^ ± 2.25 × 10^5^	7	4.64 × 10^6^ ± 1.36 × 10^5^	−20
**1b**	500	6.12 × 10^6^ ± 3.96 × 10^5^	−256	6.61 × 10^6^ ± 1.96 × 10^5^	−71
	50	2.55 × 10^6^ ± 2.96 × 10^5^	−49	5.66 × 10^6^ ± 1.64 × 10^5^	−46
	5	3.20 × 10^6^ ± 4.86 × 10^5^	−86	5.38 × 10^6^ ± 2.19 × 10^5^	−39
	0.5	1.20 × 10^6^ ± 2.66 × 10^5^	30	4.69 × 10^6^ ± 2.50 × 10^5^	−21
**1c**	500	1.86 × 10^6^ ± 8.60 × 10^4^	−8	1.56 × 10^6^ ± 1.43 × 10^5^	60
	50	2.00 × 10^6^ ± 3.07 × 10^5^	−16	3.89 × 10^6^ ± 2.62 × 10^5^	0
	5	2.93 × 10^6^ ± 1.51 × 10^6^	−70	3.48 × 10^6^ ± 1.45 × 10^5^	10
	0.5	2.16 × 10^6^ ± 3.74 × 10^5^	−25	4.13 × 10^6^ ± 1.28 × 10^5^	−7
**1d**	500	2.84 × 10^6^ ± 1.15 × 10^5^	−65	1.72 × 10^6^ ± 2.72 × 10^5^	56
	50	1.19 × 10^6^ ± 7.52 × 10^4^	31	1.48 × 10^6^ ± 2.45 × 10^5^	62
	5	1.60 × 10^6^ ± 1.91 × 10^5^	7	3.02 × 10^6^ ± 2.80 × 10^5^	22
	0.5	1.58 × 10^6^ ± 1.22 × 10^5^	8	2.47 × 10^6^ ± 2.54 × 10^5^	36
**1e**	500	1.10 × 10^6^ ± 1.62 × 10^5^	36	1.31 × 10^6^ ± 7.05 × 10^4^	66
	50	7.61 × 10^5^ ± 7.21 × 10^4^	56	9.43 × 10^5^ ± 4.39 × 10^4^	76
	5	9.22 × 10^5^ ± 8.81 × 10^4^	46	3.62 × 10^6^ ± 2.34 × 10^5^	6
	0.5	1.38 × 10^6^ ± 2.30 × 10^5^	20	2.94 × 10^6^ ± 2.72 × 10^5^	24
Control		1.72 × 10^6^ ± 1.67 × 10^5^		3.87 × 10^6^ ± 3.12 × 10^5^	

Compound **1e** showed optimal antibiofilm activity against both the bacterial strains, demonstrating efficacy at concentrations of 50 μM (56%) and 5 μM (46%) for *S. aureus*, and 500 μM (66%) and 50 μM (76%) for *E. coli*. These results highlight Compound **1e** as a promising lead candidate for further investigation. Compound **1d** also exhibited notable antibiofilm activity, specifically against *E. coli*, with a significant reduction in adhered cells observed at 50 μM (62%) and 500 μM (56%). In general, the gram‐negative bacterium *E. coli* appears more responsive to the antibiofilm activity of the selected compounds than the gram‐positive bacterium *S. aureus*.

To determine whether the observed biofilm‐modulating effects were due to a target‐mediated mechanism rather than a direct activity on ROS, the total antioxidant capacity (TAC) of **1a–e** was assessed using the Sigma‐Aldrich TAC assay (Merck KGaA, Darmstadt, Germany). None of the tested compounds exhibited significant antioxidant activity, indicating that their biological properties are not mediated by general redox scavenging (see Supporting Information S2). This supports the hypothesis that these molecules may instead act by modulating intracellular redox homeostasis through specific interactions, such as the inhibition of WrbA.

Finally, the human toxicity profiles of Compounds **1a–e** were predicted by ADMETLab3.0 server (https://admet.scbdd.com/home/index/), a comprehensive tool for evaluating ADMET properties [35]. The predictions included assessments of hERG blocking potential, human hepatotoxicity, AMES mutagenicity, skin sensitization, acute toxicity (LD_50_), drug‐induced liver injury, and maximum recommended daily dose. Notably, all compounds were predicted to exhibit human toxicity only at doses exceeding 500 mg/kg (see Supporting Information S2 for details).

## 3 Conclusions

In this study, we designed and synthesized a nature‐inspired class of 5,7‐dihydroxy‐2,2‐dimethylchroman‐4‐one derivatives as WrbA binders and evaluated their effects on biofilm modulation. Specifically, we utilized a computationally driven structure‐based approach to develop and optimize a library of analogs bearing different amide moieties. Among these compounds, the best candidates were selected for synthesis based on molecular docking, MD simulations, MM‐GBSA calculations, and synthetic feasibility. Their binding affinity to the target enzyme was analyzed by MST, revealing good‐to‐excellent *K*_d_ values. None of the tested compounds exhibited biocidal effects or served as carbon and energy sources for *E. coli* or *S. aureus*, confirming their targeted mechanism of action. Subsequent antibiofilm assays identified Compound **1e** as the most effective candidate, reaching its maximal antibiofilm activity at 50 μM for *E. coli* (76%) and *S. aureus* (56%). Conversely, other analogs exhibited a pro‐biofilm effect. As observed in our study, the biphasic role of ROS in biofilm modulation aligns with existing literature highlighting their signaling roles in bacterial communities. These findings underscore the importance of carefully modulating ROS levels to maximize antibiofilm effects without inducing pro‐biofilm responses. Future efforts will focus on refining and optimizing the biological profile of these compounds, exploring their mechanisms of action in greater detail, and evaluating their efficacy in hindering biofilm formation in different experimental settings.

## 4 Experimental

### 4.1 Docking and MD Simulations

The 3D structures of the compounds listed in Table 1 were built using the “Reaction‐Based Enumeration” tool of Maestro (release 2021‐2, Schrödinger LLC, New York, NY, USA) and prepared for docking using the “LigPrep” tool of the same molecular modeling software. The binding site of WrbA was identified based on the presence of the benzoquinone ligand in the crystal structure of the protein (PDB accession code: 4YQE) [36]. The docking grid was positioned between the flavin mononucleotide (FMN) cofactor and Trp97, which are both located in the catalytic site of the enzyme. Benzoquinone was removed from the target structure to allow docking calculations of the competitive ligands. The extra precision (XP) docking protocol was employed to generate the most reliable complexes, consisting of the best docking pose of the ligands and WrbA [37]. Next, the “System Builder” tool of Maestro and the Desmond algorithm were utilized to construct the simulation system and conduct the MD simulations, respectively. The duration of the MD simulations was determined based on the stability of the ligands in the binding site, with most compounds undergoing simulations lasting 150 ns. Ligand stability was assessed by analyzing the root‐mean‐square deviation (RMSD) of heavy atoms over time. Subsequently, the 50 ns of MD simulations (corresponding to 50 frames) in which the ligands exhibited the highest stability were selected for binding free energy calculations using the MM‐GBSA method [38]. These estimations were performed using the single‐trajectory approach, applying the default parameters of the Prime Maestro protocol. The evaluation of the entropic contribution (Δ*S*) to the ligand binding free energy (Δ*G*) was omitted due to the computational cost and uncertainty associated with the calculations. Therefore, our Δ*G* values are denoted as “ΔG*.”

### 4.2 Target‐Based Design of 5,7‐Dihydroxy‐2,2‐Dimethylchroman‐4‐one Analogs

A library of amides was generated using the Maestro “Reaction‐Based Enumeration” tool. This feature utilizes two different libraries of starting reactants to create new compounds by simulating their reaction. In this case, the reaction between the core carboxylic acid, namely 2‐[(5‐hydroxy‐2,2‐dimethyl‐4‐oxochroman‐7‐yl)oxy]acetic acid, and a database of amines was chosen to afford the corresponding amides. The library of amines included the 20 natural amino acids and a collection of achiral amines, with a maximum of 15 heavy atoms and 2 rings. This process resulted in the selection of 25,000 compounds. Docking calculations were performed using the protocol previously described for the compounds listed in Table 1. However, for these calculations, the standard precision (SP) level of accuracy was employed to enhance the calculation speed. Subsequently, the 1000 compounds with the lowest SP score, indicating the highest compatibility between the ligand and the target, were subjected to further docking in the WrbA catalytic site using the XP docking algorithm. The results were visually examined, and the amides with the most favorable combination of reactant cost and synthetic feasibility were selected. The selected compounds were then simulated in a complex with WrbA, and their ΔG* values were calculated using the MM‐GBSA approach. The Cα RMSD graphs of Compounds **1a–e** are available in Supporting Information S2: Figure 28. The computational protocol described earlier, which has been frequently used by our team and is referenced in the literature [23, 24], was employed for these calculations. The most promising compounds resulting from this process are presented in Table 2.

### 4.3 Chemistry

All starting materials and solvents were acquired from commercial sources (Merck KGaA, Darmstadt, Germany; FluoroChem, Hadfield, UK) and used as received. The reactions were monitored by thin‐layer chromatography (TLC), performed on aluminum‐backed silica gel 60 plates (0.2 mm; Merck). Purification of crude products was carried out via flash column chromatography on silica gel 60 (40–63 μM; Merck, Darmstadt, Germany) using the specified solvent system. Melting points were measured in open capillary tubes using a Stuart SMP30 Melting Point Apparatus (Cole‐Parmer Stuart, Stone, UK). Characterization of all tested compounds was performed by ^1^H and ^13^C NMR spectroscopy and high‐resolution mass spectrometry (HRMS). NMR spectra were acquired at room temperature on a Varian Oxford 300 MHz instrument (Varian, Palo Alto, CA, USA), operating at 300 MHz for ^1^H and 75 MHz for ^13^C. Chemical shifts are expressed in ppm (*δ*), and *J*‐couplings are provided in Hertz. HRMS experiments were carried out on a Q‐ToF Synapt G2‐Si HDMS spectrometer (Waters, Milford, MA, USA). Purity of the tested compounds (≥ 95%) was determined by reversed‐phase HPLC on a Waters system, using a Phenomenex Luna 3 μM C18(2) 100 Å, 100 mm × 4.6 mm column (Phenomenex, Torrance, CA, USA) in the following operative conditions—mobile phase: water/acetonitrile + 0.05% TFA (gradient mode, see Supporting Information S2 for further details); flow rate: 1 mL/min; detector *λ*: 220 nm; time: 30 min; temperature: 23°C. The synthesis of the final compounds (**1a–e**) and key intermediates (**2**–**4**) is described in the following paragraphs. The preparation of the remaining synthetic precursors (**5**, **6**) is reported in Supporting Information S2, along with all relevant spectra and chromatograms. NMR signal assignments and the atom‐numbering scheme referenced in the following section are provided in Supporting Information S2. The InChI codes of the investigated compounds, together with some biological activity data, are provided as Supporting Information S1.

#### 4.3.1 Synthesis of Compounds **2**–**4**

5,7‐Dihydroxy‐2,2‐dimethylchroman‐4‐one (**2**): Phloroglucinol (500 mg, 3.96 mmol) and 3‐methylbutenoic acid (546.6 mg, 5.46 mmol) were dissolved in dry 1,4‐dioxane (2.40 mL) under a nitrogen atmosphere. Then, PPA (1 mL) was added to the reaction using a syringe, and the mixture was stirred at 60°C for 3 h. The resulting red, dense fluid was treated with ice and a saturated solution of NaHCO_3_ to pH 7 and then extracted with EtOAc. The combined organic layers were dried over anhydrous Na_2_SO_4_, filtered, and concentrated under reduced pressure. The crude was purified by flash column chromatography (cyclohexane/EtOAc 8:2) and recrystallization from EtOAc/hexane to afford the desired compound (**2**) as a yellow solid. Yield: 50%. TLC (cyclohexane/EtOAc 7:3): *R*_f_ = 0.48. Mp: 191.3°C–192.8°C. ^1^H NMR (300 MHz, CDCl_3_) *δ* 12.02 (s exch. D_2_O, 1H, H_14_), 5.93 (d, *J* = 2.3 Hz, 1H, H_3_), 5.87 (d, *J* = 2.3 Hz, 1H, H_1_), 5.75 (br s exch. D_2_O, 1H, H_15_), 2.68 (s, 2H, H_9_), 1.45 (s, 6H, H_12,13_) ppm. ^13^C NMR (75 MHz, acetone‐*d*_*6*_) *δ* 196.03 (C_10_), 166.48 (C_2_), 164.16 (C_6_), 161.84 (C_4_), 101.61 (C_5_), 95.40 (C_1_), 95.32 (C_3_), 78.84 (C_8_), 46.93 (C_9_), 25.84 (C_12,13_) ppm.

Ethyl 2‐[(5‐hydroxy‐2,2‐dimethyl‐4‐oxochroman‐7‐yl)oxy]acetate (**3**): To a stirred solution of **2** (730 mg, 3.51 mmol) in dry DMF (8.7 mL), oven‐dried K_2_CO_3_ (968 mg, 7.02 mmol) and ethyl 2‐bromoacetate (0.47 mL, 4.21 mmol) were added at 0°C under a nitrogen atmosphere. The mixture was stirred at 0°C for 5 h. After completion, the reaction was diluted with water, acidified with 1 M HCl to pH 3–4, and extracted with EtOAc. The combined organic layers were dried over anhydrous Na_2_SO_4_, filtered, and concentrated under reduced pressure. The resulting oil was purified by flash column chromatography (cyclohexane/EtOAc 9:1) to yield Compound **3** as a yellow solid. Yield: 93%. TLC (cyclohexane/EtOAc 8:2): *R*_f_ = 0.52. Mp: 81.0°C–82.5°C. ^1^H NMR (300 MHz, CDCl_3_) *δ* 11.97 (s exch. D_2_O, 1H, H_15_), 5.98 (d, *J* = 2.3 Hz, 1H, H_3_), 5.94 (d, *J* = 2.3 Hz, 1H, H_1_), 4.60 (s, 2H, H_16_), 4.27 (q, *J* = 7.2 Hz, 2H, H_20_), 2.68 (s, 2H, H_9_), 1.45 (s, 6H, H_12,13_), 1.30 (t, *J* = 7.2 Hz, 3H, H_21_) ppm. ^13^C NMR (75 MHz, CDCl_3_) *δ* 196.24 (C_10_), 167.92 (C_17_), 165.93 (C_2_), 163.81 (C_6_), 161.49 (C_4_), 102.92 (C_5_), 94.97 (C_1_), 94.80 (C_3_), 79.12 (C_8_), 65.02 (C_16_), 61.63 (C_20_), 47.68 (C_9_), 26.69 (C_12,13_), 14.11 (C_21_) ppm.

2‐[(5‐Hydroxy‐2,2‐dimethyl‐4‐oxochroman‐7‐yl)oxy]acetic acid (**4**): Compound **3** (920 mg, 3.12 mmol) was dissolved in a 9:1 EtOH/H_2_O solution (19.5 mL). The mixture was cooled to 0°C, 2.5 M NaOH (7.36 mL) was added, and the reaction was stirred for 30 min until the completion of the hydrolysis, as indicated by TLC. Then, the mixture was acidified with 1 M HCl to pH 3–4, and the pure compound (**4**) was recovered by filtration as a white solid. Yield: 96%. TLC (DCM/MeOH 8:2): *R*_f_ = 0.32. Mp: 198.5°C–201.5°C. ^1^H NMR (300 MHz, CD_3_OD) *δ* 5.99 (d, *J* = 2.4 Hz, 1H, H_3_), 5.97 (d, *J* = 2.4 Hz, 1H, H_1_), 4.65 (s, 2H, H_16_), 2.73 (s, 2H, H_9_), 1.43 (s, 6H, H_12,13_) ppm. ^13^C NMR (75 MHz, DMSO‐*d*_*6*_) *δ* 197.13 (C_10_), 169.91 (C_17_), 166.41 (C_2_), 163.32 (C_6_), 161.55 (C_4_), 102.47 (C_5_), 95.04 (C_1_), 94.92 (C_3_), 79.80 (C_8_), 65.13 (C_16_), 47.16 (C_9_), 26.54 (C_13,14_) ppm.

#### 4.3.2 General Procedure for the Synthesis of 1a–e

To a solution of Compound **4** (1 mmol) in dry DMF (4 mL), 1‐[bis(dimethylamine)methylene]‐1*H*‐1,2,3‐triazolo[4,5‐*b*]pyridinium3‐oxid hexafluorophosphate (HATU) (1 mmol) and *N*,*N*‐diisopropylethylamine (DIPEA) (0.7 mL) were added under a nitrogen atmosphere. The reaction mixture was stirred for 45 min at room temperature; then, the appropriate amine (1 mmol; or 3 mmol of NH_4_Cl for **1a**) was added, and the stirring was continued overnight. For the synthesis of **1a**, **1c**, and **1d**, commercially available amines were used, while for **1b** and **1e**, intermediates **5** and **6** were, respectively, employed (see Supporting Information S2). The reaction mixture was diluted with EtOAc and washed two times with a saturated solution of NaHCO_3_ and once with brine. The organic layer was dried over anhydrous Na_2_SO_4_, filtered, and evaporated under reduced pressure. The crude product was purified by flash column chromatography in the indicated eluent to afford the desired products **1a–e**.

2‐[(5‐Hydroxy‐2,2‐dimethyl‐4‐oxochroman‐7‐yl)oxy]acetamide (**1a**): Starting reagent: NH_4_Cl. Purification: cyclohexane/EtOAc 4:6. Aspect: white solid. Yield: 45%. TLC (cyclohexane/EtOAc 4:6): *R*_f_ = 0.29. Mp: 172.6°C–174.1°C. ^1^H NMR (300 MHz, CDCl_3_) *δ* 11.97 (s exch. D_2_O, 1H, H_19_), 6.40 (br s exch. D_2_O, 1H, H_4_), 6.03 (d, *J* = 2.4 Hz, 1H, H_13_), 5.96 (d, *J* = 2.4 Hz, 1H, H_11_), 5.58 (br s exch. D_2_O, 1H, H_4_), 4.49 (s, 2H, H_2_), 2.70 (s, 2H, H_7_), 1.46 (s, 6H, H_17,18_) ppm. ^13^C NMR (75 MHz, CDCl_3_) *δ* 196.31 (C_8_), 164.90 (C_1_), 163.90 (C_10_), 163.41 (C_12_), 161.75 (C_14_), 103.17 (C_9_), 95.21 (C_11_), 94.59 (C_13_), 79.36 (C_6_), 66.89 (C_2_), 47.70 (C_7_), 26.67 (C_17,18_) ppm. HRMS (ESI/Q‐ToF): *m*/*z* calcd. for C_13_H_15_NO_5_Na 288.0848, found 288.0845.

2‐[(5‐Hydroxy‐2,2‐dimethyl‐4‐oxochroman‐7‐yl)oxy]‐*N*‐{2‐[(naphthalen‐1 ylmethyl)amino]ethyl}acetamide (**1b**): Starting reagent: *N* ^1^‐(naphthalen‐1‐ylmethyl)ethane‐1,2‐diamine (**5**). Purification: DCM/MeOH 95:5. Aspect: light‐brown solid. Yield: 25%. TLC (DCM/MeOH 9:1): *R*_f_ = 0.62. Mp: 115.8°C–117.2°C. ^1^H NMR (300 MHz, CDCl_3_) *δ* 11.92 (br s exch. D_2_O, 1H, H_11_), 8.12 (d, *J* = 7.6 Hz, 1H, H_30_), 7.86 (dd, *J* = 7.6, 1.8 Hz, 1H, H_27_), 7.78 (dd, *J* = 7.3, 2.2 Hz, 1H, H_33_), 7.55–7.39 (m, 4H, H_28,29,31,32_), 7.02 (br s exch. D_2_O, 1H, H_18_), 5.99 (d, *J* = 2.3 Hz, 1H, H_3_), 5.92 (d, *J* = 2.3 Hz, 1H, H_1_), 4.42 (s, 2H, H_16_), 4.25 (s, 2H, H_23_), 3.46 (q, *J* = 5.7 Hz, 2H, H_20_), 2.92 (t, *J* = 5.7 Hz, 2H, H_21_), 2.66 (s, 2H, H_9_), 1.89 (br s exch. D_2_O, 1H, H_22_), 1.44 (s, 6H, H_13,14_) ppm. ^13^C NMR (75 MHz, CDCl_3_) *δ* 196.25 (C_10_), 167.14 (C_17_), 165.08 (C_6_), 163.85 (C_2_), 161.64 (C_4_), 135.14 (C_26_), 133.89 (C_25_), 131.70 (C_33_), 128.77 (C_29_), 128.10 (C_27_), 126.29 (C_32_), 126.27 (C_28_), 125.77 (C_31_), 125.35 (C_24_), 123.54 (C_30_), 103.06 (C_5_), 95.25 (C_1_), 94.73 (C_3_), 79.23 (C_8_), 67.09 (C_16_), 51.21 (C_23_), 48.13 (C_9_), 47.67 (C_21_), 38.61 (C_20_), 26.68 (C_13,14_) ppm. HRMS (ESI/Q‐ToF): *m*/*z* calcd. for C_26_H_29_N_2_O_5_ 449.2076, found 449.2083; *m*/*z* calcd. for C_26_H_28_N_2_O_5_Na 471.1896, found 471.1896.

*N*‐[2‐(Benzylamino)ethyl]‐2‐[(5‐hydroxy‐2,2‐dimethyl‐4‐oxochroman‐7‐yl)oxy]acetamide (**1c**): Starting reagent: *N* ^1^‐benzylethane‐1,2‐diamine. Purification: DCM/MeOH 9:1. Aspect: yellow solid. Yield: 87%. TLC (DCM/MeOH 9:1): *R*_f_ = 0.55. Mp: 100.2°C–101.7°C. ^1^H NMR (300 MHz, CDCl_3_) *δ* 7.35–7.22 (m, 5H, H_25,26,27,28,29_ partially overlapped with solvent peak), 7.08 (br s exch. D_2_O, 1H, H_18_), 6.05 (d, *J* = 2.4 Hz, 1H, H_3_), 5.97 (d, *J* = 2.4 Hz, 1H, H_1_), 4.46 (s, 2H, H_16_), 3.79 (s, 2H, H_23_), 3.43 (q, *J* = 5.8 Hz, 2H, H_20_), 2.83–2.79 (m, 3H, H_21_), 2.68 (s, 2H, H_9_), 1.44 (s, 6H, H_12,13_) ppm. ^13^C NMR (75 MHz, CDCl_3_) *δ* 196.27 (C_10_), 167.26 (C_17_), 165.13 (C_2_), 163.87 (C_6_), 161.70 (C_4_), 139.38 (C_24_), 128.53 (C_26,28_), 128.16 (C_25,29_), 127.29 (C_27_), 103.08 (C_5_), 95.23 (C_1_), 94.69 (C_3_), 79.27 (C_8_), 67.12 (C_16_), 53.27 (C_23_), 47.67 (C_9_), 47.59 (C_21_), 38.46 (C_20_), 26.67 (C_12,13_) ppm. HRMS (ESI/Q‐ToF): *m*/*z* calcd. for C_22_H_27_N_2_O_5_ 399.1920, found 399.1919; *m*/*z* calcd. for C_22_H_26_N_2_O_5_Na 421.1739, found 421.1738.

*N*‐(3‐Chlorobenzyl)‐2‐[(5‐hydroxy‐2,2‐dimethyl‐4‐oxochroman‐7‐yl)oxy]acetamide (**1d**): Starting reagent: (3‐chlorophenyl)methanamine. Purification: cyclohexane/EtOAc 7:3. Aspect: yellowish semisolid. Yield: 57%. TLC (cyclohexane/EtOAc 7:3): *R*_f_ = 0.35. ^1^H NMR (300 MHz, CDCl_3_) *δ* 11.95 (s exch. D_2_O, 1H, H_14_), 7.29–7.24 (m, 3H, H_22,25,26_ partially overlapped with solvent peak), 7.18–7.14 (m, 1H, H_24_), 6.84 (br s exch. D_2_O, 1H, H_18_), 6.01 (d, *J* = 2.4 Hz, 1H, H_3_), 5.94 (d, *J* = 2.4 Hz, 1H, H_1_), 4.53–4.49 (m, 4H, H_16,20_), 2.68 (s, 2H, H_9_), 1.42 (s, 6H, H_12,13_) ppm. ^13^C NMR (75 MHz, CDCl_3_) *δ* 196.32 (C_10_), 167.18 (C_17_), 164.84 (C_6_), 163.87 (C_2_), 161.74 (C_4_), 139.66 (C_21_), 134.60 (C_23_), 130.03 (C_25_), 127.85 (C_22_), 127.72 (C_24_), 125.80 (C_26_), 103.16 (C_5_), 95.23 (C_1_), 94.62 (C_3_), 79.34 (C_8_), 67.08 (C_16_), 47.66 (C_9_), 42.41 (C_20_), 26.66 (C_12,13_) ppm. HRMS (ESI/Q‐ToF): *m*/*z* calcd. for C_20_H_20_NO_5_NaCl 412.0928, found 412.0925.

2‐[(5‐Hydroxy‐2,2‐dimethyl‐4‐oxochroman‐7‐yl)oxy]‐*N*‐(naphthalen‐1‐ylmethyl)acetamide (**1e**): Starting reagent: naphthalen‐1‐ylmethanamine (**6**). Purification: cyclohexane/EtOAc 7:3. Aspect: off‐white solid. Yield: 42%. TLC (cyclohexane/EtOAc 7:3): *R*_f_ = 0.38. Mp: 90.5°C–93.5°C. ^1^H NMR (300 MHz, CDCl_3_) *δ* 11.92 (s exch. D_2_O, 1H, H_15_), 7.99 (d, *J* = 7.4 Hz, 1H, H_30_), 7.89–7.81 (m, 2H, H_24,27_), 7.58–7.40 (m, 4H, H_22,23,28,29_), 6.72 (br s exch. D_2_O, 1H, H_18_), 5.95 (d, *J* = 2.4 Hz, 1H, H_3_), 5.88 (d, *J* = 2.4 Hz, 1H, H_1_), 4.99 (d, *J* = 5.6 Hz, 2H, H_20_), 4.53 (s, 2H, H_16_), 2.66 (s, 2H, H_9_), 1.42 (s, 6H, H_12,13_) ppm. ^13^C NMR (75 MHz, CDCl_3_) *δ* 196.25 (C_10_), 166.75 (C_17_), 164.85 (C_2_), 163.82(C_6_), 161.62 (C_4_), 133.87 (C_21_), 132.77 (C_25_), 131.29 (C_26_), 128.86 (C_27_), 128.85 (C_24_), 126.77 (C_23_), 126.70 (C_28_), 126.08 (C_29_), 125.36 (C_22_), 123.21 (C_30_), 103.09 (C_5_), 95.18 (C_1_), 94.67 (C_3_), 79.26 (C_8_), 67.09 (C_16_), 47.66 (C_9_), 41.22 (C_20_), 26.64 (C_12,13_) ppm. HRMS (ESI/Q‐ToF): *m*/*z* calcd. for C_24_H_23_NO_5_Na 428.1474, found 428.1475.

### 4.4 Crystallography

Crystals of **4** were obtained as colorless tabular prisms from the slow evaporation of a 1:1 MeOH/water solution. Diffraction data were collected at 293 K on an Enraf‐Nonius CAD4 four‐circle diffractometer, using graphite‐monochromatized Mo‐Kα X‐radiation (*λ* = 0.7107 Å). Data collection was performed in the 2*θ* range 3.8°–60.2°, employing a profiled ω‐scan mode with scan angles of (1.20 + 0.35 tan *θ*)° and a prescan speed of 3.30° min^−1^. Accurate unit‐cell parameters were determined via a least‐squares fit of 2*θ* values from 25 reflections within the 2*θ* range 15.8°–30.6°. Data reduction—including intensity integration, background subtraction, and Lorentz and polarization corrections—was carried out using the WinGX package [39]. Absorption effects were assessed with the psi‐scan method [40], and a corresponding correction was applied to the data (min./max. transmission factors were 0.955/0.992). The structure was solved in the triclinic space group P−1 by direct methods using SIR2019 [41] and completed through iterative full‐matrix least‐squares refinement on *F*_o_^2^ and Δ*F* synthesis using SHELXL‐2019/2 [42], within the WinGX suite (WinGX v.2023.1) [39]. Hydrogen atoms bonded to carbon were placed in geometrically calculated positions and refined using a riding model with fixed isotropic thermal parameters (1.2 U_eq_ and 1.5 U_eq_ of the parent atom for methylene and methyl groups, respectively). Hydrogen atoms bonded to oxygen were located in a difference‐Fourier map and refined freely. The structure was analyzed with CCDC Mercury (v.2022.3.0) [43], PARST [44], and CrystalExplorer (v.21.5) [45]. Graphical representations were generated using Mercury and CrystalExplorer.

Crystal data for **4**: Formula: C_13_H_14_O_6_; MW = 266.24 g/mol; crystal system: triclinic; space group: P−1; cell dimensions: *a* = 5.5149(12) Å, *b* = 10.6529(12) Å, *c* = 10.8699(19) Å, *α* = 103.704(11)°, *β* = 95.187(11)°, *γ* = 95.346(12)°; *V* = 613.53(19) Å^3^; *Z* = 2; *D*_calc_ = 1.441 Mg/m^3^; 2*θ*_min_ = 1.942°; 2*θ*_max_ = 26.369°; limiting indices = −6 ≤ *h* ≤ 6, −13 ≤ *k* ≤ 12, −1 ≤ *l* ≤ 13; crystal size: 0.52 × 0.29 × 0.10 mm; *T* = 293(2) K; *F*(000) = 280; *R*_int_ = 0.0145; data/restraints/parameters: 2510/0/182; *R* = 0.0485 for 2044 reflections with *F*_o_ > 4*σ*(*F*_o_) (*R* = 0.0616 for all 2510 unique/2694 collected reflections), wR2 = 0.1122 for reflections with *F*_o_ > 4*σ*(*F*_o_) (wR2 = 0.1247 for all unique reflections); GOOF = 1.085; residual positive and negative electron densities in the final map: 0.184 and −0.185 eÅ^−3^. CCDC accession number: 2345344.

### 4.5 Biophysical Assays

MST experiments were conducted on a Monolith NT.115 instrument (NanoTemper Technologies GmbH, Munich, Germany), using the WrbA recombinant protein, produced by us according to the method described in our previous work [23]. In this case, the MST *K*_d_ curves were normalized by fraction bound. Supporting Information S2 includes detailed affinity curves for each compound, along with a summary table of the experimental conditions applied. All the figures were generated using GraphPad Prism software v8.0.2 (GraphPad, Boston, MA, USA).

### 4.6 Bacterial Strain and Growth Conditions

The bacterial biofilm model systems employed in this study were *E. coli* K‐12 wild‐type strain ATCC 25404 and Methicillin‐resistant *S. aureus* (MRSA) strain ATCC 43300. The strains were stored at −80°C in suspensions containing 20% glycerol and 2% peptone. For routine cultivation, the strains were grown in Tryptic Soy Broth (TSB, Sigma‐Aldrich, St. Louis, MO, USA) at a temperature of 37°C for 15 h.

### 4.7 Planktonic Growth

To assess whether the bacterial strains could utilize the selected compounds as their sole carbon and energy source, growth experiments in PBS (Sigma‐Aldrich/Merck KGaA, Darmstadt, Germany) supplemented with 500 μM of each compound were conducted. Stock solutions in DMSO (100 mM) were prepared for each compound, ensuring the complete solubility of the molecules. Working solutions were prepared in PBS. At the highest concentration tested (500 μM), the final DMSO content in the biological system was well below the 3% threshold reported in the literature as nontoxic to bacterial cells [21, 46]. Microbial growth was monitored by measuring absorbance at 600 nm (*A*_600_). Each experiment was performed in triplicate. The planktonic growth of both *E. coli* and *S. aureus* in a TSB medium supplemented with either 0 μM (control) or 500 μM of the test compound was also evaluated. Experiments were conducted in 96‐well microtiter plates at 37°C, with growth curves generated using the Infinite F200 PRO microplate reader (TECAN, Männedorf, Switzerland). To begin the experiments, 3 μL (3% v/v) of overnight bacterial culture was added to each well and adjusted to a final concentration of 10^6^ cells/mL. The *A*_600_ readings were taken every 10 min over a 24‐h period. Growth curves were constructed by plotting the difference between the *A*_600_ of cultures with and without the compounds, subtracting the *A*_600_ of non‐inoculated medium against incubation time. The data were fitted to the Gompertz model using XLSTAT software (Version 2022.2.1, Addinsoft, Paris, France). From these fits, we calculated the maximum growth rate (*A*_600_; min). Each condition was tested in triplicate.

### 4.8 Antibiofilm Assays

To assess the effect of the selected compounds on cell adhesion, we conducted a quantitative analysis using fluorochrome‐labeled cells in hydrophobic 96‐well black‐sided plates, following the methodology outlined by Ratti et al. [24]. Each well contained 200 μL of PBS and 10⁷ cells, with individual compounds at concentrations of 0, 0.5, 5, 50, or 500 μM. We selected 500 μM as the highest working concentration, as this dose had previously been tested for antibiofilm activity with other compounds [23, 24]. The plates were incubated for 18 h at 37°C. Post‐incubation, wells were washed twice with 200 μL of PBS, and adhered cells were stained with 10 μM SYTO 9 (ThermoFisher Scientific, Waltham, MA, USA) in PBS for 20 min at room temperature. Fluorescence intensity was measured using the Infinite F200 PRO microplate reader (TECAN) with excitation at 483 nm and emission at 503 nm. A standard curve correlating fluorescence intensity with cell number was generated to quantify the antibiofilm effect of the compounds. Biofilm inhibition was determined by calculating the percentage reduction in adhered cells compared to control groups. Each experimental condition was tested in six biological replicates. According to the classification proposed by Cattò et al. [21], compounds were categorized based on their ability to reduce the number of adhered bacterial cells compared to the negative control. Compounds reducing adhesion by less than 20% were classified as having no antibiofilm activity; reductions between 20% and 30% indicated low antibiofilm activity; reductions between 30% and 40% indicated moderate activity; and reductions exceeding 40% were considered indicative of optimal antibiofilm activity.

## Conflicts of Interest

The authors declare no conflicts of interest.

## Supporting information

Supporting Information.

Supporting Information.

## Data Availability

The data that support the findings of this study are available from the corresponding author upon reasonable request.

## References

[1] K. Sauer , P. Stoodley , D. M. Goeres , et al., “The Biofilm Life Cycle: Expanding the Conceptual Model of Biofilm Formation,” Nature Reviews Microbiology 20 (2022): 608–620. 35922483 10.1038/s41579-022-00767-0 PMC9841534

[2] O. Ciofu , C. Moser , P. Ø. Jensen , and N. Høiby , “Tolerance and Resistance of Microbial Biofilms,” Nature Reviews Microbiology 20 (2022): 621–635. 35115704 10.1038/s41579-022-00682-4

[3] D. Sharma , L. Misba , and A. U. Khan , “Antibiotics Versus Biofilm: An Emerging Battleground in Microbial Communities,” Antimicrobial Resistance & Infection Control 8 (2019): 76. 31131107 10.1186/s13756-019-0533-3 PMC6524306

[4] S. Singh , S. K. Singh , I. Chowdhury , and R. Singh , “Understanding the Mechanism of Bacterial Biofilms Resistance to Antimicrobial Agents,” Open Microbiology Journal 11 (2017): 53–62. 28553416 10.2174/1874285801711010053 PMC5427689

[5] V. S. Gondil and B. Subhadra , “Biofilms and Their Role on Diseases,” BMC Microbiology 23 (2023): 203. 37525111 10.1186/s12866-023-02954-2 PMC10391977

[6] G. Shineh , M. Mobaraki , M. J. Perves Bappy , and D. K. Mills , “Biofilm Formation, and Related Impacts on Healthcare, Food Processing and Packaging, Industrial Manufacturing, Marine Industries, and Sanitation—A Review,” Applied Microbiology 3 (2023): 629–665.

[7] S. L. Percival , L. Suleman , C. Vuotto , and G. Donelli , “Healthcare‐Associated Infections, Medical Devices and Biofilms: Risk, Tolerance and Control,” Journal of Medical Microbiology 64 (2015): 323–334. 25670813 10.1099/jmm.0.000032

[8] D. Gulotta , F. Villa , F. Cappitelli , and L. Toniolo , “Biofilm Colonization of Metamorphic Lithotypes of a Renaissance Cathedral Exposed to Urban Atmosphere,” Science of the Total Environment 639 (2018): 1480–1490. 29929311 10.1016/j.scitotenv.2018.05.277

[9] L. A. Philipp , K. Bühler , R. Ulber , and J. Gescher , “Beneficial Applications of Biofilms,” Nature Reviews Microbiology 22 (2023): 276–290. 37957398 10.1038/s41579-023-00985-0

[10] S. Saini , S. Tewari , J. Dwivedi , and V. Sharma , “Biofilm‐Mediated Wastewater Treatment: A Comprehensive Review,” Materials Advances 4 (2023): 1415–1443.

[11] M. H. Muhammad , A. L. Idris , X. Fan , et al., “Beyond Risk: Bacterial Biofilms and Their Regulating Approaches,” Frontiers in Microbiology 11 (2020): 928. 32508772 10.3389/fmicb.2020.00928 PMC7253578

[12] G. Annunziato , “Strategies to Overcome Antimicrobial Resistance (AMR) Making Use of Non‐Essential Target Inhibitors: A Review,” International Journal of Molecular Sciences 20 (2019): 5844. 31766441 10.3390/ijms20235844 PMC6928725

[13] R. Srinivasan , S. Santhakumari , P. Poonguzhali , M. Geetha , M. Dyavaiah , and L. Xiangmin , “Bacterial Biofilm Inhibition: A Focused Review on Recent Therapeutic Strategies for Combating the Biofilm Mediated Infections,” Frontiers in Microbiology 12 (2021): 676458. 34054785 10.3389/fmicb.2021.676458 PMC8149761

[14] V. C. Kalia , S. K. S. Patel , and J. K. Lee , “Bacterial Biofilm Inhibitors: An Overview,” Ecotoxicology and Environmental Safety 264 (2023): 115389. 37634478 10.1016/j.ecoenv.2023.115389

[15] S. Buroni and L. R. Chiarelli , “Antivirulence Compounds: A Future Direction to Overcome Antibiotic Resistance,” Future Microbiology 15 (2020): 299–301. 32286100 10.2217/fmb-2019-0294

[16] D. Papadimou , E. Malmqvist , and M. Ancillotti , “Socio‐Cultural Determinants of Antibiotic Resistance: A Qualitative Study of Greeks' Attitudes, Perceptions and Values,” BMC Public Health 22 (2022): 1439. 35902816 10.1186/s12889-022-13855-w PMC9333897

[17] K. W. K. Tang , B. C. Millar , and J. E. Moore , “Antimicrobial Resistance (AMR),” British Journal of Biomedical Science 80 (2023): 11387. 37448857 10.3389/bjbs.2023.11387 PMC10336207

[18] M. Gambino and F. Cappitelli , “Mini‐Review: Biofilm Responses to Oxidative Stress,” Biofouling 32 (2016): 167–178. 26901587 10.1080/08927014.2015.1134515

[19] F. Rossi , C. Cattò , G. Mugnai , F. Villa , and F. Forlani , “Effects of the Quinone Oxidoreductase WrbA on *Escherichia coli* Biofilm Formation and Oxidative Stress,” Antioxidants 10 (2021): 919. 34204135 10.3390/antiox10060919 PMC8229589

[20] E. V. Patridge and J. G. Ferry , “WrbA From *Escherichia coli* and *Archaeoglobus fulgidus* Is an NAD(P)H:Quinone Oxidoreductase,” Journal of Bacteriology 188 (2006): 3498–3506. 16672604 10.1128/JB.188.10.3498-3506.2006 PMC1482846

[21] C. Cattò , S. Dell'Orto , F. Villa , et al., “Unravelling the Structural and Molecular Basis Responsible for the Anti‐Biofilm Activity of Zosteric Acid,” PLoS One 10 (2015): e0131519. 26132116 10.1371/journal.pone.0131519 PMC4488431

[22] C. Cattò , G. Grazioso , S. Dell'Orto , et al., “The Response of *Escherichia coli* Biofilm to Salicylic Acid,” Biofouling 33 (2017): 235–251. 28270055 10.1080/08927014.2017.1286649

[23] A. Ratti , E. M. A. Fassi , F. Forlani , et al., “Mechanistic Insights Into the Antibiofilm Mode of Action of Ellagic Acid,” Pharmaceutics 15 (2023): 1757. 37376205 10.3390/pharmaceutics15061757 PMC10302398

[24] A. Ratti , E. M. A. Fassi , F. Forlani , et al., “Unlocking the Antibiofilm Potential of Natural Compounds by Targeting the NADH:Quinone Oxidoreductase WrbA,” Antioxidants 12 (2023): 1612. 37627607 10.3390/antiox12081612 PMC10451263

[25] S. Kamboj and R. Singh , “Chromanone—A Prerogative Therapeutic Scaffold: An Overview,” Arabian Journal for Science and Engineering 47 (2021): 75–111. 34226859 10.1007/s13369-021-05858-3 PMC8244469

[26] S. Emami and Z. Ghanbarimasir , “Recent Advances of Chroman‐4‐One Derivatives: Synthetic Approaches and Bioactivities,” European Journal of Medicinal Chemistry 93 (2015): 539–563. 25743215 10.1016/j.ejmech.2015.02.048

[27] D. Cremer and J. A. Pople , “General Definition of Ring Puckering Coordinates,” Journal of the American Chemical Society 97 (1975): 1354–1358.

[28] C. Jelsch , K. Ejsmont , and L. Huder , “The Enrichment Ratio of Atomic Contacts in Crystals, an Indicator Derived From the Hirshfeld Surface Analysis,” IUCrJ 1 (2014): 119–128. 10.1107/S2052252514003327 PMC4062089 25075328

[29] P. Kovacic , “Unifying Mechanism for Bacterial Cell Signalers (4,5‐Dihydroxy‐2,3‐Pentanedione, Lactones and Oligopeptides): Electron Transfer and Reactive Oxygen Species. Practical Medical Features,” Medical Hypotheses 69 (2007): 1105–1110. 17445992 10.1016/j.mehy.2007.01.085

[30] O. Nasution , K. Srinivasa , M. Kim , et al., “Hydrogen Peroxide Induces Hyphal Differentiation in *Candida albicans* ,” Eukaryotic Cell 7 (2008): 2008–2011. 18791036 10.1128/EC.00105-08 PMC2583538

[31] J. Checa and J. M. Aran , “Reactive Oxygen Species: Drivers of Physiological and Pathological Processes,” Journal of Inflammation Research 13 (2020): 1057–1073. 33293849 10.2147/JIR.S275595 PMC7719303

[32] M. Čáp , L. Váchová , and Z. Palková , “Reactive Oxygen Species in the Signaling and Adaptation of Multicellular Microbial Communities,” Oxidative Medicine and Cellular Longevity 2012 (2012): 976753. 22829965 10.1155/2012/976753 PMC3395218

[33] B. R. Boles and P. K. Singh , “Endogenous Oxidative Stress Produces Diversity and Adaptability in Biofilm Communities,” Proceedings of the National Academy of Sciences 105 (2008): 12503–12508. 10.1073/pnas.0801499105 PMC2527941 18719125

[34] S. L. Chua , Y. Ding , Y. Liu , et al., “Reactive Oxygen Species Drive Evolution of Pro‐Biofilm Variants in Pathogens by Modulating Cyclic‐di‐GMP Levels,” Open Biology 6 (2016): 160162. 27881736 10.1098/rsob.160162 PMC5133437

[35] L. Fu , S. Shi , J. Yi , et al., “ADMETlab 3.0: An Updated Comprehensive Online ADMET Prediction Platform Enhanced With Broader Coverage, Improved Performance, API Functionality and Decision Support,” Nucleic Acids Research 52 (2024): W422–W431. 38572755 10.1093/nar/gkae236 PMC11223840

[36] O. Degtjarik , J. Brynda , O. Ettrichova , et al., “Quantum Calculations Indicate Effective Electron Transfer between FMN and Benzoquinone in a New Crystal Structure of *Escherichia coli* WrbA,” Journal of Physical Chemistry B 120 (2016): 4867–4877. 27183467 10.1021/acs.jpcb.5b11958

[37] R. A. Friesner , R. B. Murphy , M. P. Repasky , et al., “Extra Precision Glide: Docking and Scoring Incorporating a Model of Hydrophobic Enclosure for Protein−Ligand Complexes,” Journal of Medicinal Chemistry 49 (2006): 6177–6196. 17034125 10.1021/jm051256o

[38] S. Genheden and U. Ryde , “The MM/PBSA and MM/GBSA Methods to Estimate Ligand‐Binding Affinities,” Expert Opinion on Drug Discovery 10 (2015): 449–461. 25835573 10.1517/17460441.2015.1032936 PMC4487606

[39] L. J. Farrugia , “WinGX and ORTEP for Windows: An Update,” Journal of Applied Crystallography 45 (2012): 849–854.

[40] A. C. T. North , D. C. Phillips , and F. S. Mathews , “A Semi‐Empirical Method of Absorption Correction,” Acta Crystallographica Section A 24 (1968): 351–359.

[41] M. C. Burla , R. Caliandro , B. Carrozzini , et al., “Crystal Structure Determination and Refinement via SIR2014,” Journal of Applied Crystallography 48 (2015): 306–309.

[42] G. M. Sheldrick , “Crystal Structure Refinement With SHELXL,” Acta Crystallographica, Section C: Structural Chemistry 71 (2015): 3–8. 25567568 10.1107/S2053229614024218 PMC4294323

[43] C. F. MacRae , I. Sovago , S. J. Cottrell , et al., “Mercury 4.0: From Visualization to Analysis, Design and Prediction,” Journal of Applied Crystallography 53 (2020): 226–235. 32047413 10.1107/S1600576719014092 PMC6998782

[44] M. Nardelli , “Parst: A System of Fortran Routines for Calculating Molecular Structure Parameters From Results of Crystal Structure Analyses,” Computers & Chemistry 7 (1983): 95–98.

[45] P. R. Spackman , M. J. Turner , J. J. McKinnon , et al., “CrystalExplorer: A Program for Hirshfeld Surface Analysis, Visualization and Quantitative Analysis of Molecular Crystals,” Journal of Applied Crystallography 54 (2021): 1006–1011. 34188619 10.1107/S1600576721002910 PMC8202033

[46] C. Cattò , E. M. A. Fassi , G. Grazioso , A. Gelain , S. Villa , and F. Cappitelli , “Insights on Zosteric Acid Analogues Activity Against *Candida albicans* Biofilm Formation,” ACS Omega 10 (2025): 22285–22295. 40488070 10.1021/acsomega.5c03581 PMC12138599

