# Supplementary material for: Nature‐Inspired Compounds Targeting Escherichia coli WrbA as Biofilm‐Modulating Agents: Computational Design, Synthesis, and Biological Evaluation
Source: Arch Pharm (Weinheim). 2025 Jul 11;358(7):e70049. doi: 10.1002/ardp.70049 (PMC12247152; doi:10.1002/ardp.70049)
Supplement: Supplementary file 1 — Supporting Information. [file ARDP-358-e70049-s002.docx]

**Supplemental Material: Novel Compounds and Biological Screening Results**

**Nature-inspired compounds targeting *E. coli* WrbA as biofilm-modulating agents: computational design, synthesis, and biological evaluation**

Matteo Mori^1^, Enrico Mario Alessandro Fassi^1^, Federica Villa^2^, Erica Ginevra Milano^1^, Fabio Forlani^2^, Francesca Cappitelli^2^, Alessandro Ratti^1^, Fiorella Meneghetti^1^, Gabriella Roda^1^, Giovanni Grazioso^1,*^, Stefania Villa^1,*^

^1^Department of Pharmaceutical Sciences, University of Milan, Via L. Mangiagalli 25, 20133 Milano, Italy

^2^Department of Food, Environmental and Nutritional Sciences, University of Milan, Via L. Mangiagalli 25, 20133 Milano, Italy

*Correspondence: Prof. Giovanni Grazioso, Department of Pharmaceutical Sciences, University of Milan, Via L. Mangiagalli 25, 20133 Milano, Italy; Prof. Stefania Villa, Department of Pharmaceutical Sciences, University of Milan, Via L. Mangiagalli 25, 20133 Milano, Italy

Email: giovanni.grazioso@unimi.it, stefania.villa@unimi.it

| **Compound No.** | **InChI** | **Biological Activity (% antibiofilm effect)^a^** | |
| --- | --- | --- | --- |
|  |  | ***S. aureus*** | ***E. coli*** |
| **1a** | InChI=1S/C13H15NO5/c1-13(2)5-9(16)12-8(15)3-7(4-10(12)19-13)18-6-11(14)17/h3-4,15H,5-6H2,1-2H3,(H2,14,17) | -47% (500 μM) | -51% (500 μM) |
|  |  | -25% (50 μM) | -43% (50 μM) |
|  |  | -50% (5 μM) | -30% (5 μM) |
|  |  | 7% (0.5 μM) | -20% (0.5 μM) |
| **1b** | InChI=1S/C26H28N2O5/c1-26(2)14-22(30)25-21(29)12-19(13-23(25)33-26)32-16-24(31)28-11-10-27-15-18-8-5-7-17-6-3-4-9-20(17)18/h3-9,12-13,27,29H,10-11,14-16H2,1-2H3,(H,28,31) | -256% (500 μM) | -71% (500 μM) |
|  |  | -49% (50 μM) | -46% (50 μM) |
|  |  | -86% (5 μM) | -39% (5 μM) |
|  |  | 30% (0.5 μM) | -21% (0.5 μM) |
| **1c** | InChI=1S/C22H26N2O5/c1-22(2)12-18(26)21-17(25)10-16(11-19(21)29-22)28-14-20(27)24-9-8-23-13-15-6-4-3-5-7-15/h3-7,10-11,23,25H,8-9,12-14H2,1-2H3,(H,24,27) | -8% (500 μM) | 60% (500 μM) |
|  |  | -16% (50 μM) | 0% (50 μM) |
|  |  | -70% (5 μM) | 10% (5 μM) |
|  |  | -25% (0.5 μM) | -7% (0.5 μM) |
| **1d** | InChI=1S/C20H20ClNO5/c1-20(2)9-16(24)19-15(23)7-14(8-17(19)27-20)26-11-18(25)22-10-12-4-3-5-13(21)6-12/h3-8,23H,9-11H2,1-2H3,(H,22,25) | -65% (500 μM) | 56% (500 μM) |
|  |  | 31% (50 μM) | 62% (50 μM) |
|  |  | 7% (5 μM) | 22% (5 μM) |
|  |  | 8% (0.5 μM) | 36% (0.5 μM) |
| **1e** | InChI=1S/C24H23NO5/c1-24(2)12-20(27)23-19(26)10-17(11-21(23)30-24)29-14-22(28)25-13-16-8-5-7-15-6-3-4-9-18(15)16/h3-11,26H,12-14H2,1-2H3,(H,25,28) | 36% (500 μM) | 66% (500 μM) |
|  |  | 56% (50 μM) | 76% (50 μM) |
|  |  | 46% (5 μM) | 6% (5 μM) |
|  |  | 20% (0.5 μM) | 24% (0.5 μM) |

^a^To assess the effect of **1a-e** on cell adhesion, a quantitative analysis using fluorochrome-labeled cells in hydrophobic 96-well black-sided plates was conducted, according to the methodology outlined in the following paper: A. Ratti, E. M. A. Fassi, F. Forlani, M. Zangrossi, M. Mori, F. Cappitelli, G. Roda, S. Villa, F. Villa, G. Grazioso, *Antioxidants* **2023**, *12*, 1612.
