# Supplementary material for: Nature‐Inspired Compounds Targeting Escherichia coli WrbA as Biofilm‐Modulating Agents: Computational Design, Synthesis, and Biological Evaluation
Source: Arch Pharm (Weinheim). 2025 Jul 11;358(7):e70049. doi: 10.1002/ardp.70049 (PMC12247152; doi:10.1002/ardp.70049)
Supplement: Supplementary file 2 — Supporting Information. [file ARDP-358-e70049-s001.docx]

**Supporting Information**

**Nature-inspired compounds targeting *E. coli* WrbA as biofilm-modulating agents: computational design, synthesis, and biological evaluation**

Matteo Mori^1^, Enrico Mario Alessandro Fassi^1^, Federica Villa^2^, Erica Ginevra Milano^1^, Fabio Forlani^2^, Francesca Cappitelli^2^, Alessandro Ratti^1^, Fiorella Meneghetti^1^, Gabriella Roda^1^, Giovanni Grazioso^1,^*, and Stefania Villa^1,^*

^1^ Department of Pharmaceutical Sciences, University of Milan, Via L. Mangiagalli 25, 20133 Milano, Italy

^2^ Department of Food, Environmental and Nutritional Sciences, University of Milan, Via L. Mangiagalli 25, 20133 Milano, Italy

* Corresponding authors: giovanni.grazioso@unimi.it, stefania.villa@unimi.it

**Table of contents**

| **Chemistry – Additional Experimental Procedures** | **S2** |
| --- | --- |
| **Chemistry – Analytical characterization** | **S4** |
| **Crystallographic analysis of 4** | **S25** |
| **Biophysical Investigation – Detailed MST assays** | **S29** |
| **MD simulations – RMSD graphs of compounds 1a-e** | **S30** |
| **Total Antioxidant Capacity (TAC) of 1a-e** | **S31** |
| **Predicted human toxicity of compounds 1a-e** | **S32** |

**Chemistry – Additional Experimental Procedures**

***N^1^-(Naphthalen-1-ylmethyl)ethane-1,2-diamine (5)***

**Reagents and conditions:** *a.* TEA, THF, reflux, overnight, N_2_; *b.* TFA, DCM, RT, 2.5 h, N_2_.

*N*^1^-Boc-ethane-1,2-diamine (0.045 mL, 0.28 mmol) was added to a solution of 1-(chloromethyl)naphthalene (50 mg, 0.28 mmol) and TEA (0.078 mL, 0.56 mmol) in dry THF (1.65 mL) under a nitrogen atmosphere. The reaction was stirred at reflux overnight. After completion, the mixture was cooled down to room temperature and filtered. The organic layer was evaporated under reduced pressure, and the resulting crude was purified by flash column chromatography (DCM/MeOH 95:5) to afford the desired product (*tert*-butyl (2-((naphthalen-1-ylmethyl)amino)ethyl)carbamate, **7**) as a yellow oil. Yield: 73%. TLC (DCM/MeOH 9:1): R_f_ = 0.68. ^1^H NMR (300 MHz, CDCl_3_) δ 8.12 (d, *J* = 8.2 Hz, 1H, H_10_), 7.88 – 7.85 (m, 1H, H_7_), 7.78 (dd, *J* = 7.7, 1.5 Hz, 1H, H_6_), 7.56 – 7.39 (m, 4H, H_4,5,8,9_), 4.93 (br s exch. D_2_O, 1H, H_15_), 4.24 (s, 2H, H_11_), 3.30 – 3.24 (m, 2H, H_14_), 2.86 (t, *J* = 5.9 Hz, 2H, H_13_), 1.44 (s, 9H, H_20,21,22_) ppm. ^13^C NMR (75 MHz, CDCl_3_) δ 156.17 (C_16_), 135.63 (C_3_), 133.89 (C_1_), 131.75 (C_2_), 128.73 (C_7_), 127.87 (C_6_), 126.12 (2C, C_5,9_), 125.66 (C_4_), 125.36 (C_8_), 123.65 (C_10_), 79.07 (C_19_), 51.22 (C_11_), 48.97 (C_13_), 40.28 (C_14_), 28.43 (3C, C_20,21,22_) ppm.

To a solution of compound **7** (150 mg, 0.5 mmol) in dry DCM (2.8 mL) was added TFA (2.8 mL). The reaction was stirred at room temperature under a nitrogen atmosphere for 2.5 h. Then, the mixture was evaporated under reduced pressure, and the resulting oil was repeatedly washed with hexane and evaporated *in vacuo*. The brown solid residue was dissolved in 5 mL of 1 M HCl, and then 1M NaOH was added until pH 10-11. The aqueous phase was extracted five times with DCM. The combined organic layers were dried over anhydrous Na_2_SO_4_, filtered, and concentrated under reduced pressure to yield the pure compound **5** as a yellow oil. Yield: 98%. TLC (DCM/MeOH 9:1): R_f_ = 0.21. ^1^H NMR (300 MHz, CDCl_3_) δ 8.14 (d, *J* = 8.1 Hz, 1H, H_10_), 7.88 – 7.85 (m, 1H, H_7_), 7.78 – 7.76 (m, 1H, H_6_), 7.56 – 7.40 (m, 4H, H_4,5,8,9_), 4.26 (s, 2H, H_11_), 2.87 – 2.80 (m, 4H, H_13,14_) ppm. ^13^C NMR (75 MHz, CDCl_3_) δ 135.90 (C_3_), 133.88 (C_1_), 131.78 (C_2_), 128.72 (C_7_), 127.78 (C_6_), 126.09 (C_5_), 125.05 (C_9_), 125.62 (C_4_), 125.37 (C_8_), 123.65 (C_10_), 52.19 (C_11_), 51.53 (C_13_), 41.67 (C_14_) ppm.

***Naphthalen-1-ylmethanamine (6)***

**Reagents and conditions:** *1.* Potassium phthalimide, DMF, RT, overnight, N_2_; *2.* NH_2_NH_2_·H_2_O, EtOH, reflux, 4 h, N_2_.

To a solution of 1-(chloromethyl) naphthalene (100 mg, 0.56 mmol) in dry DMF (0.56 mL) was added phthalimide potassium salt (114 mg, 0.62 mmol). The reaction was stirred overnight at room temperature under a nitrogen atmosphere. Then, water was added, and the white precipitate was removed by filtration and dried under vacuum.

The crude phthalimide derivative was suspended in EtOH (1 mL), and hydrazine hydrate (12.3 mmol, 0.6 mL) was added. The reaction was refluxed for 4 h. After cooling to room temperature, the reaction mixture was diluted with water and DCM and extracted four times with DCM. The combined organic layers were dried over anhydrous Na_2_SO_4_, filtered, and concentrated under reduced pressure to afford the desired compound as a light-yellow oil of sufficient purity. Washing with few drops of EtOAc leads to precipitation of pure **6** as a white solid. Warning! The compound is unstable to air and light. Yield: 72%. TLC (CHCl_3_/MeOH/NH_3_ 10:1:2 drops): R_f_ = 0.62. ^1^H NMR (300 MHz, CDCl_3_) δ 8.08 (d, *J* = 8.1 Hz, 1H, H_3_), 7.88 (dd, *J* = 8.1, 1.2 Hz, 1H, H_6_), 7.77 (dd, *J* = 7.6, 1.9 Hz, 1H, H_10_), 7.58 – 7.42 (m, 4H, H_1,2,8,9_), 4.35 (s, 2H, H_11_), 1.25 (s exch. D_2_O, 2H, H_12_) ppm. ^13^C NMR (75 MHz, CDCl_3_) δ 138.94 (C_7_), 133.90 (C_5_), 131.23 (C_4_), 128.87 (C_6_), 127.54 (C_10_), 126.19 (C_2_), 125.72 (C_9_), 125.66 (C_1_), 124.46 (C_8_), 123.25 (C_3_), 43.99 (C_11_) ppm. Analytical data are consistent with the literature (*J. Med. Chem.* **2021**, *64*, 18114–18142).

**Chemistry – Analytical characterization**

***2-((5-Hydroxy-2,2-dimethyl-4-oxochroman-7-yl)oxy)acetamide (1a)***


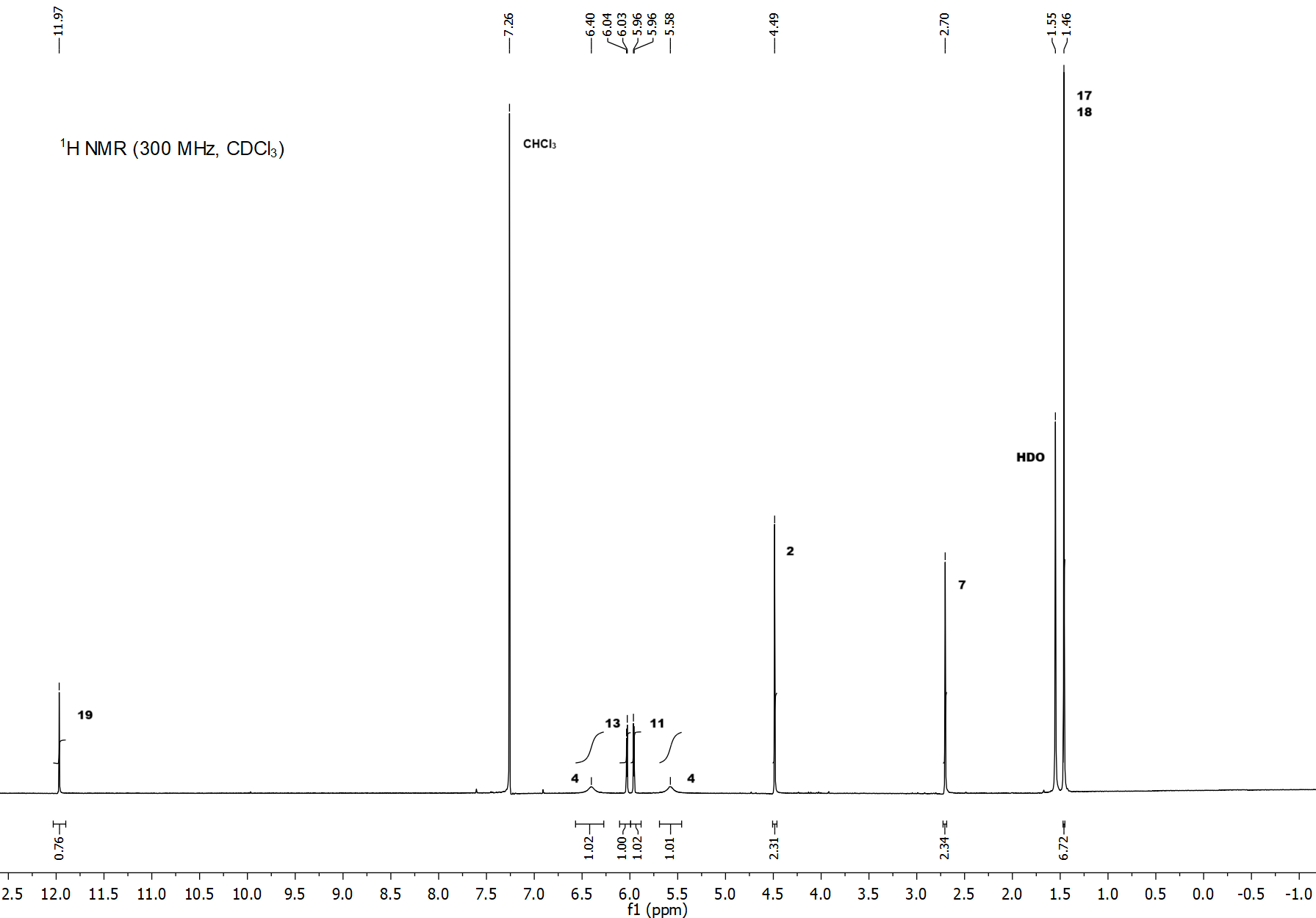


Figure S1. ^1^H NMR spectrum of 1a.


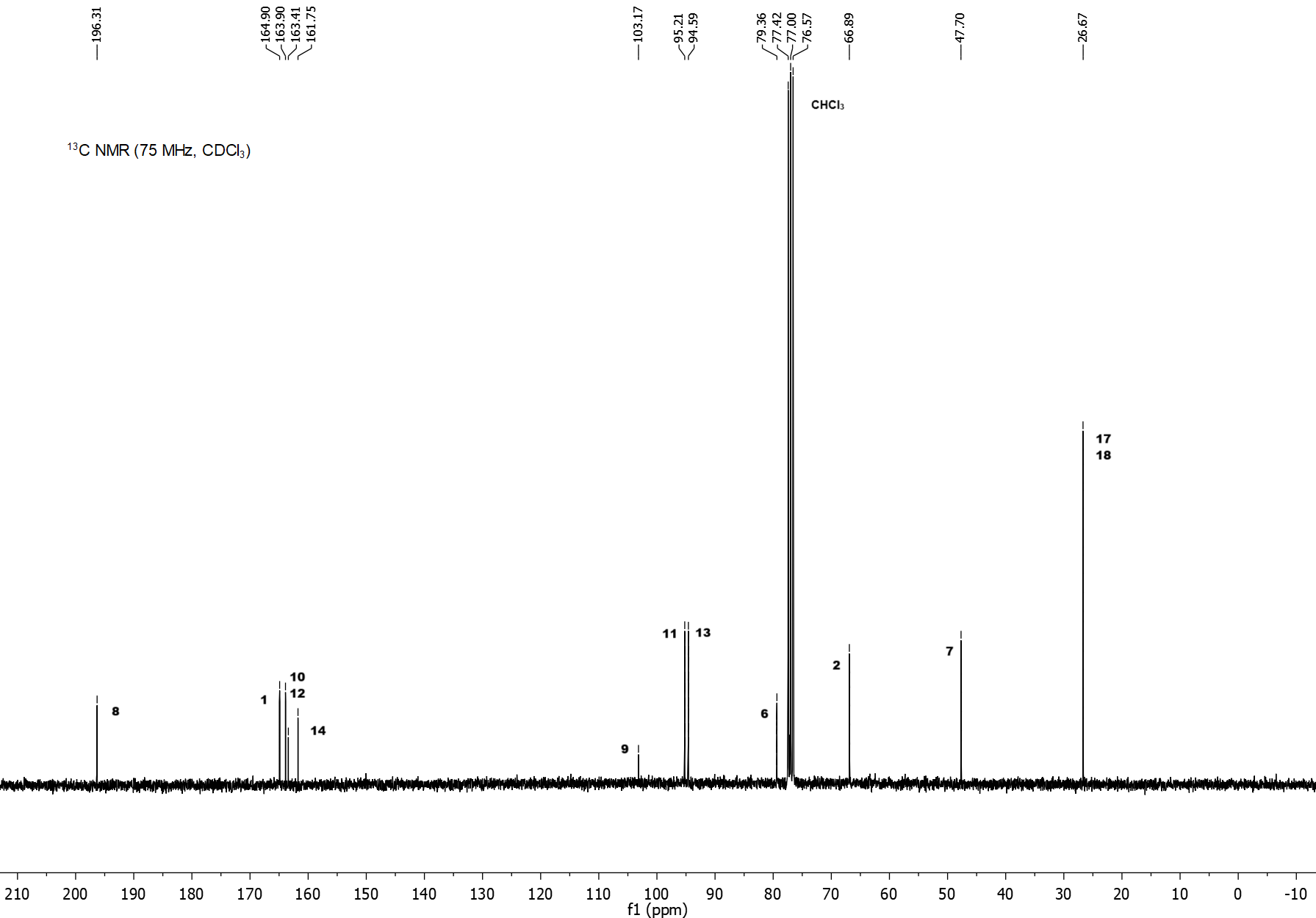


Figure S2. ^13^C NMR spectrum of 1a.


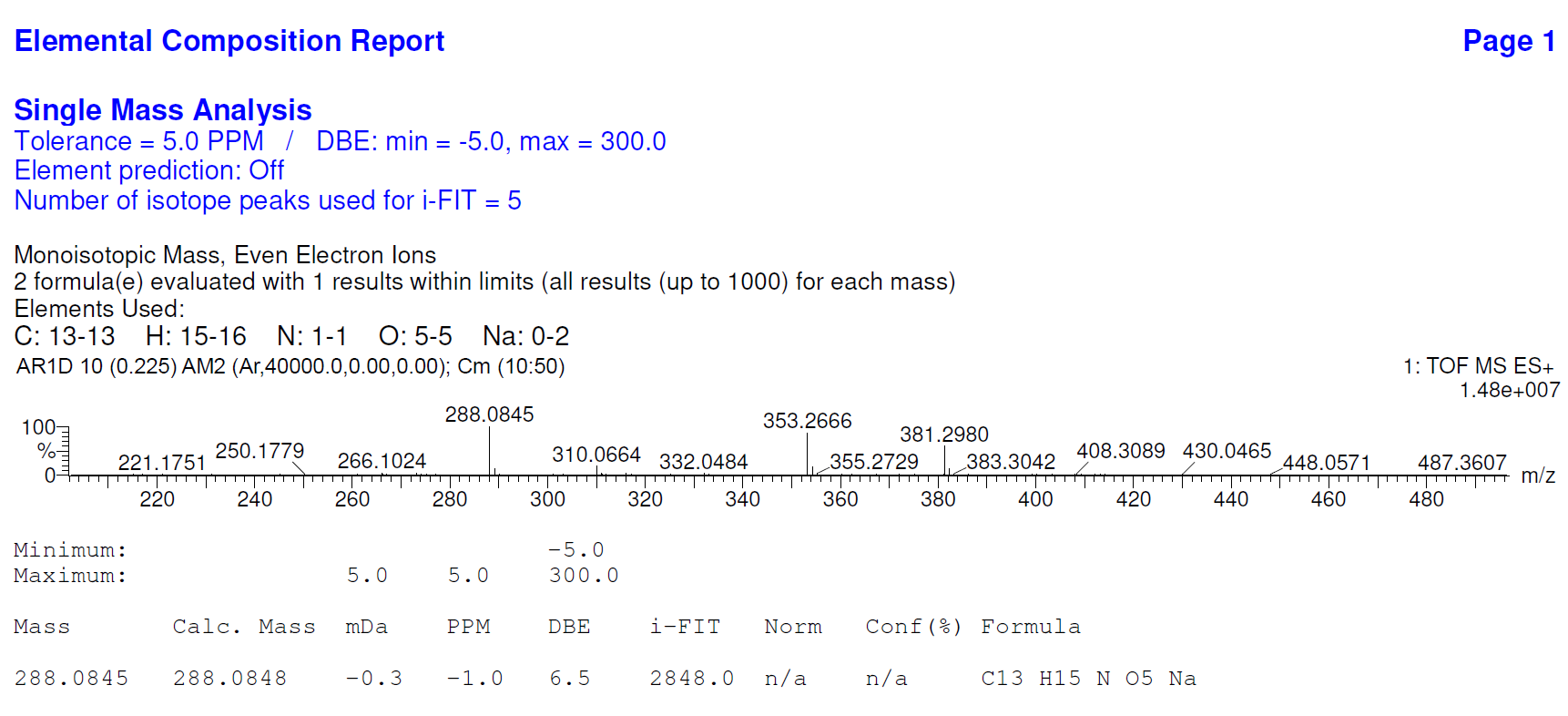


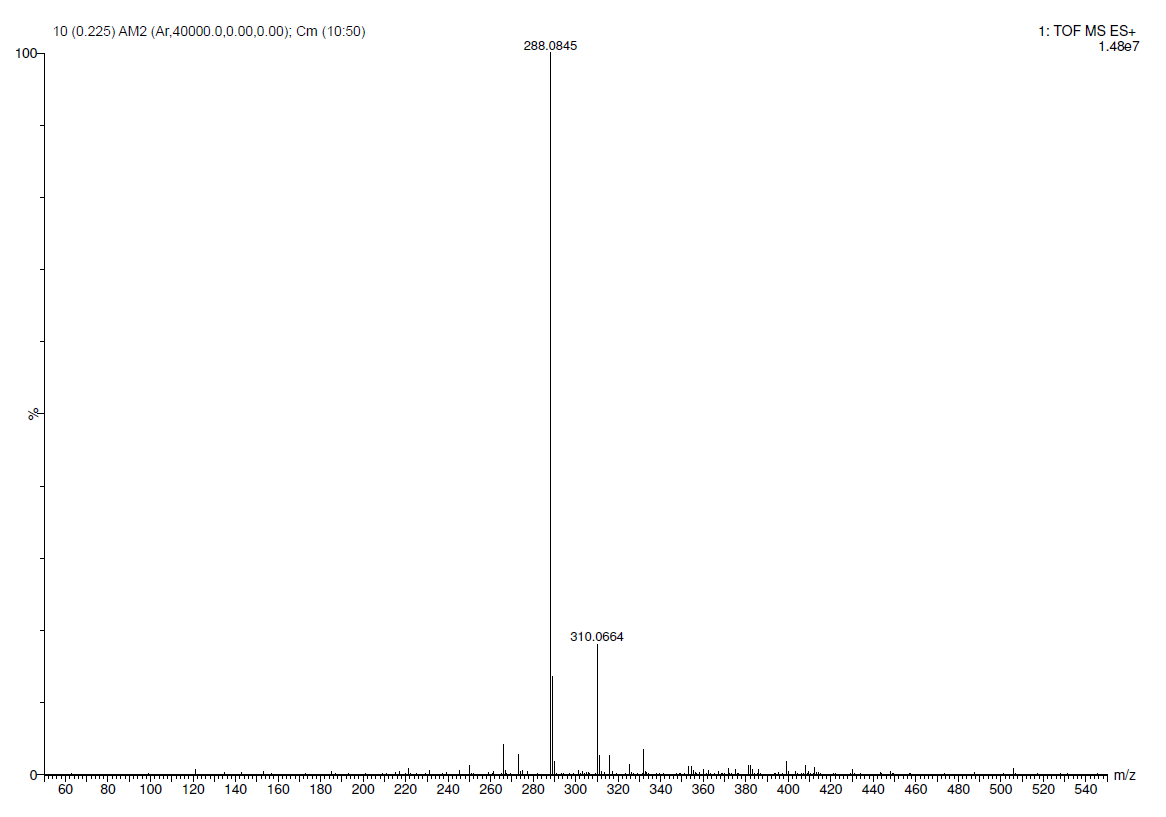


Figure S3. HRMS spectrum of 1a.

| 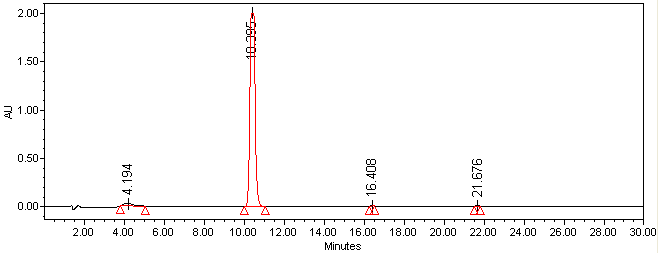 | **Retention time (min)** | **Area (%)** |
| --- | --- | --- |
|  | 4.194 | 2.32 |
|  | 10.395 | 97.43 |
|  | 16.408 | 0.18 |
|  | 21.676 | 0.08 |
|  |  |  |

Figure S4. HPLC chromatogram of 1a. Solvent A: 0.05% TFA in H_2_O. Solvent B: 0.05% TFA in MeCN. Method: linear-gradient mode, 30% B → 30% B (5 min) → 70% B (25 min). Total run time: 30 min. Flow rate: 1 mL/min. Detector wavelength: 220 nm.

***2-((5-Hydroxy-2,2-dimethyl-4-oxochroman-7-yl)oxy)-N-(2-((naphthalen-1 ylmethyl)amino)ethyl)acetamide (1b)***


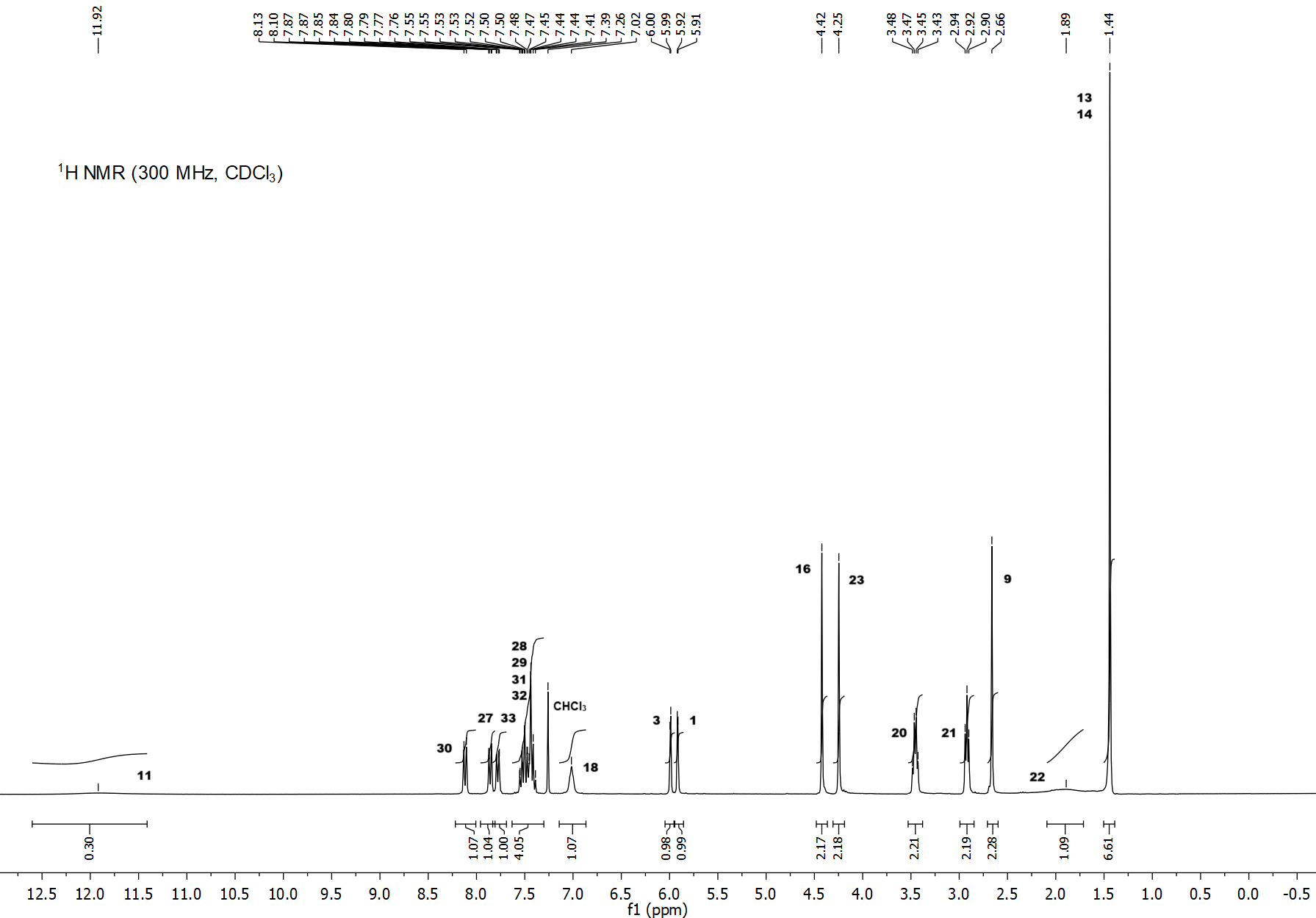


Figure S5. ^1^H NMR spectrum of 1b.


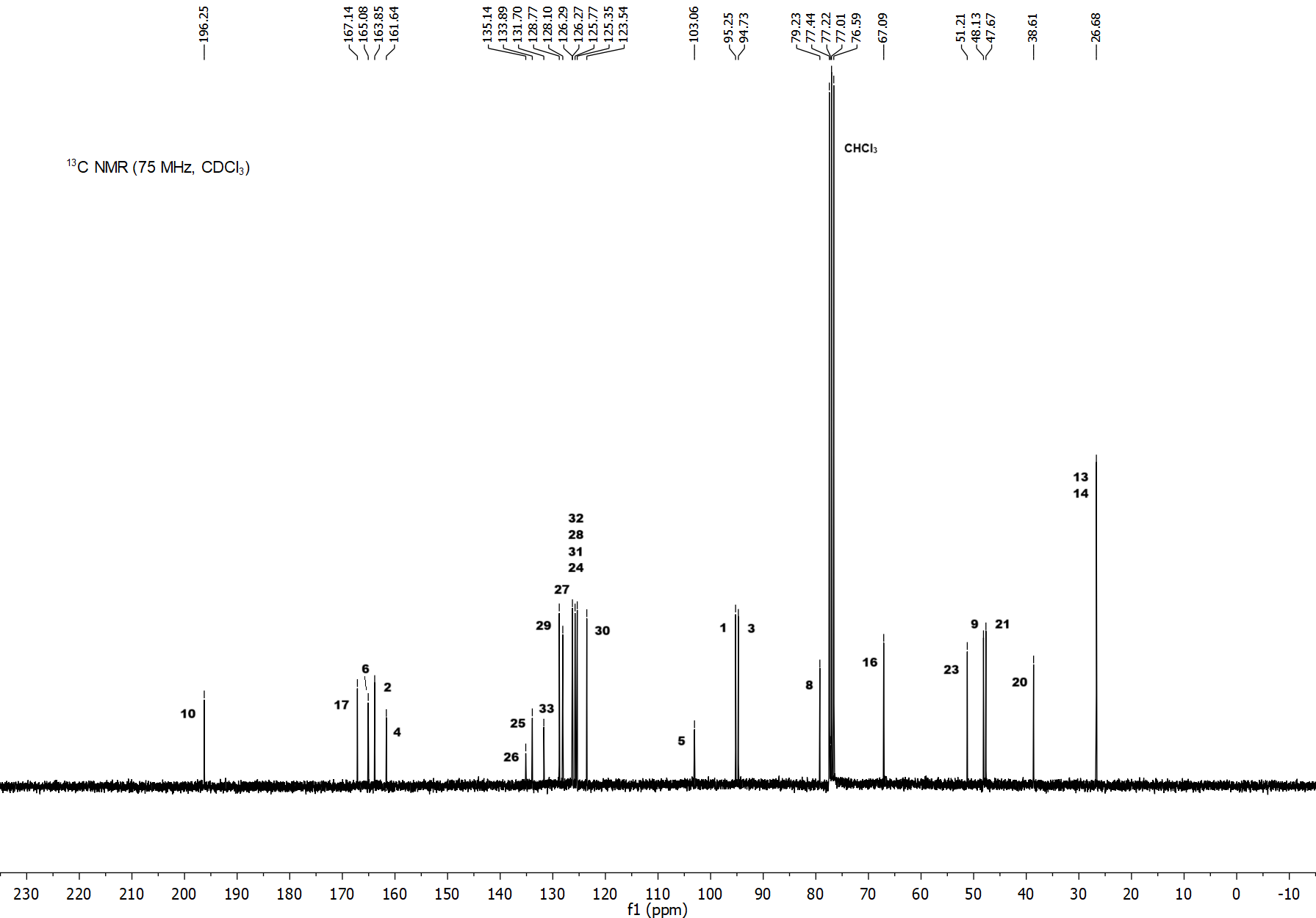


Figure S6. ^13^C NMR spectrum of 1b.

***
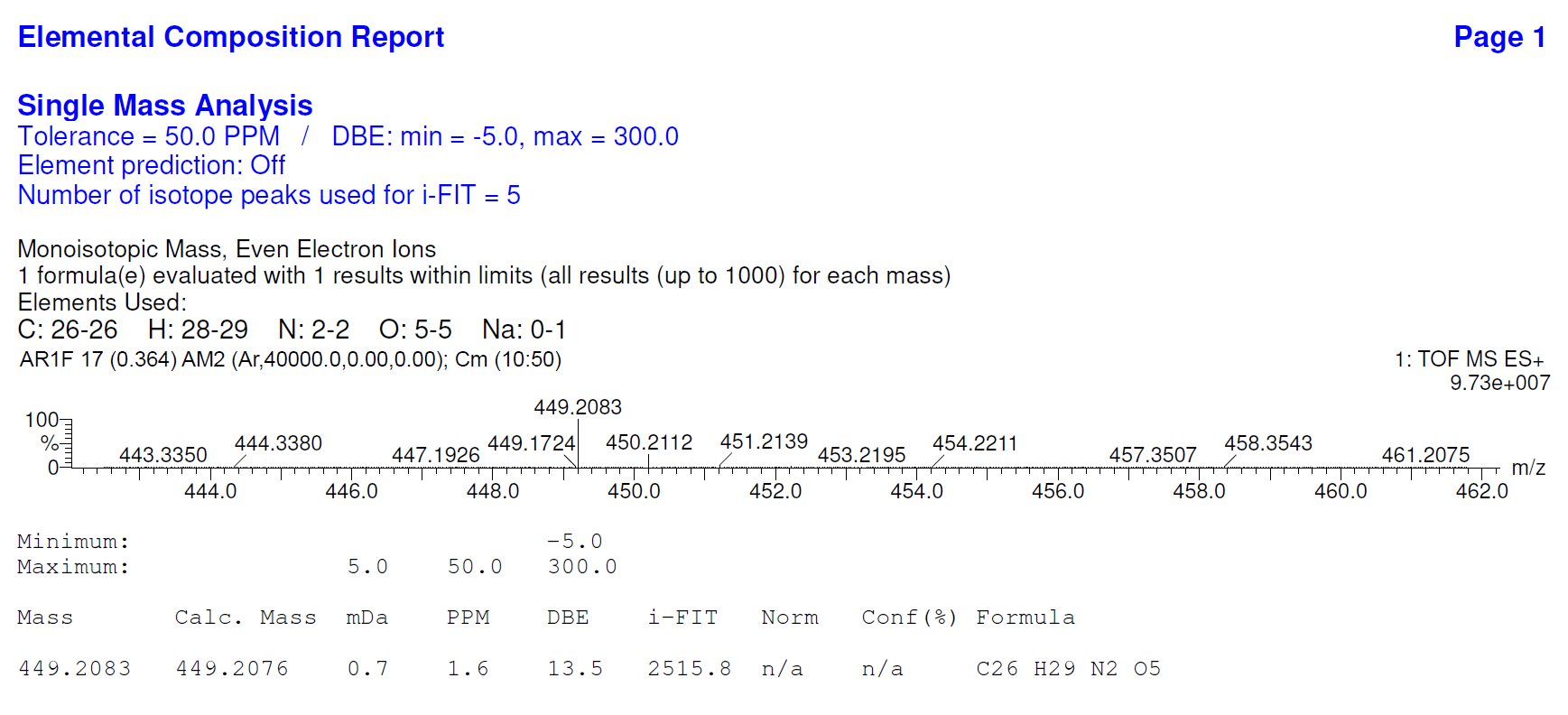
***

***
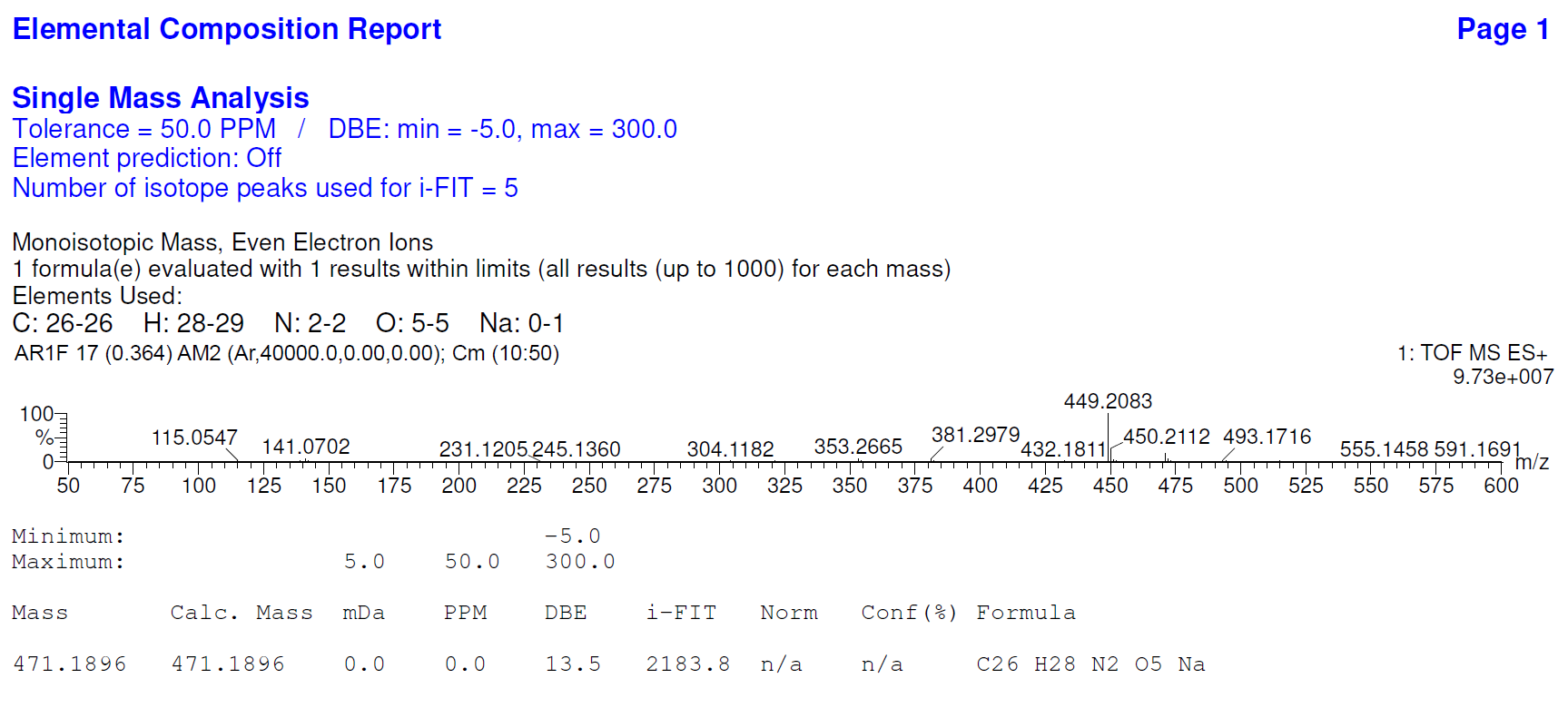
***

***
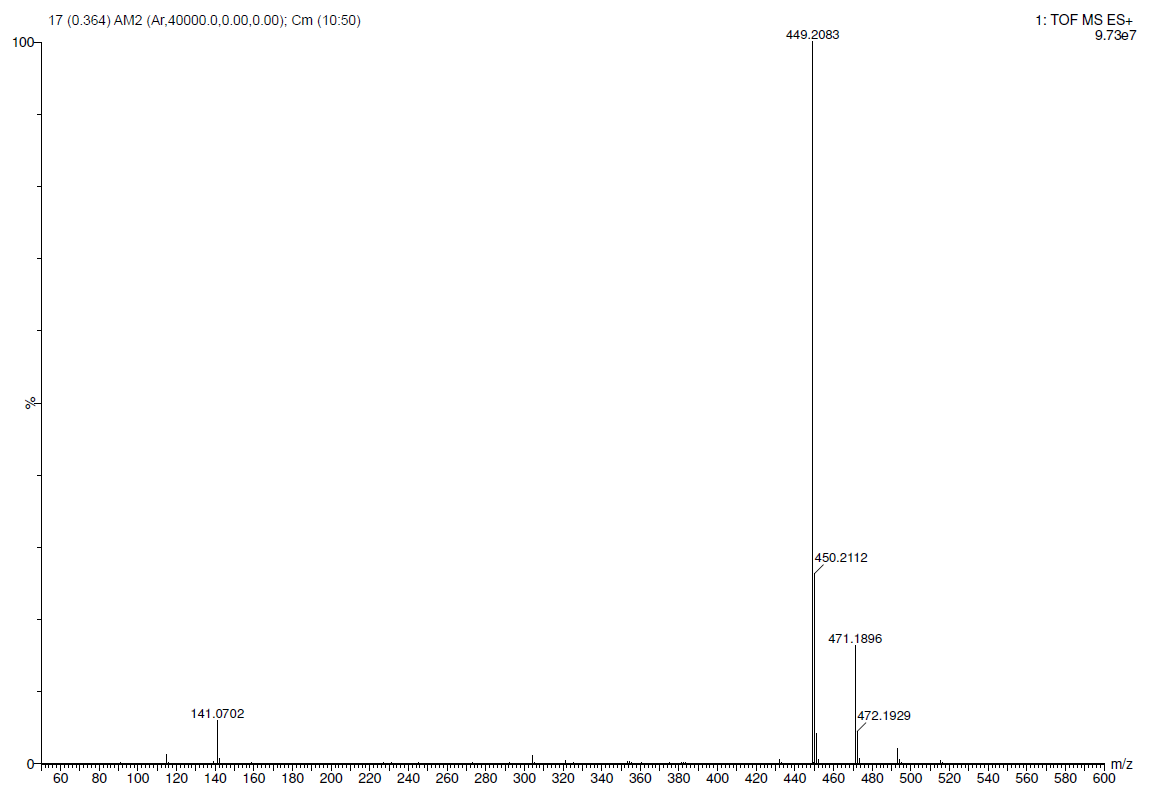
***

Figure S7. HRMS spectrum of 1b.

| 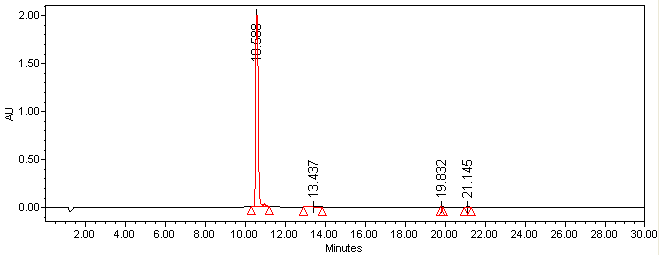 | **Retention time (min)** | **Area (%)** |
| --- | --- | --- |
|  | 10.588 | 99.27 |
|  | 13.437 | 0.48 |
|  | 19.832 | 0.02 |
|  | 21.145 | 0.22 |
|  |  |  |

Figure S8. HPLC chromatogram of 1b. Solvent A: 0.05% TFA in H_2_O. Solvent B: 0.05% TFA in MeCN. Method: linear-gradient mode, 30% B → 30% B (5 min) → 70% B (25 min). Total run time: 30 min. Flow rate: 1 mL/min. Detector wavelength: 220 nm.

***N-(2-(Benzylamino)ethyl)-2-((5-hydroxy-2,2-dimethyl-4-oxochroman-7-yl)oxy)acetamide (1c)***


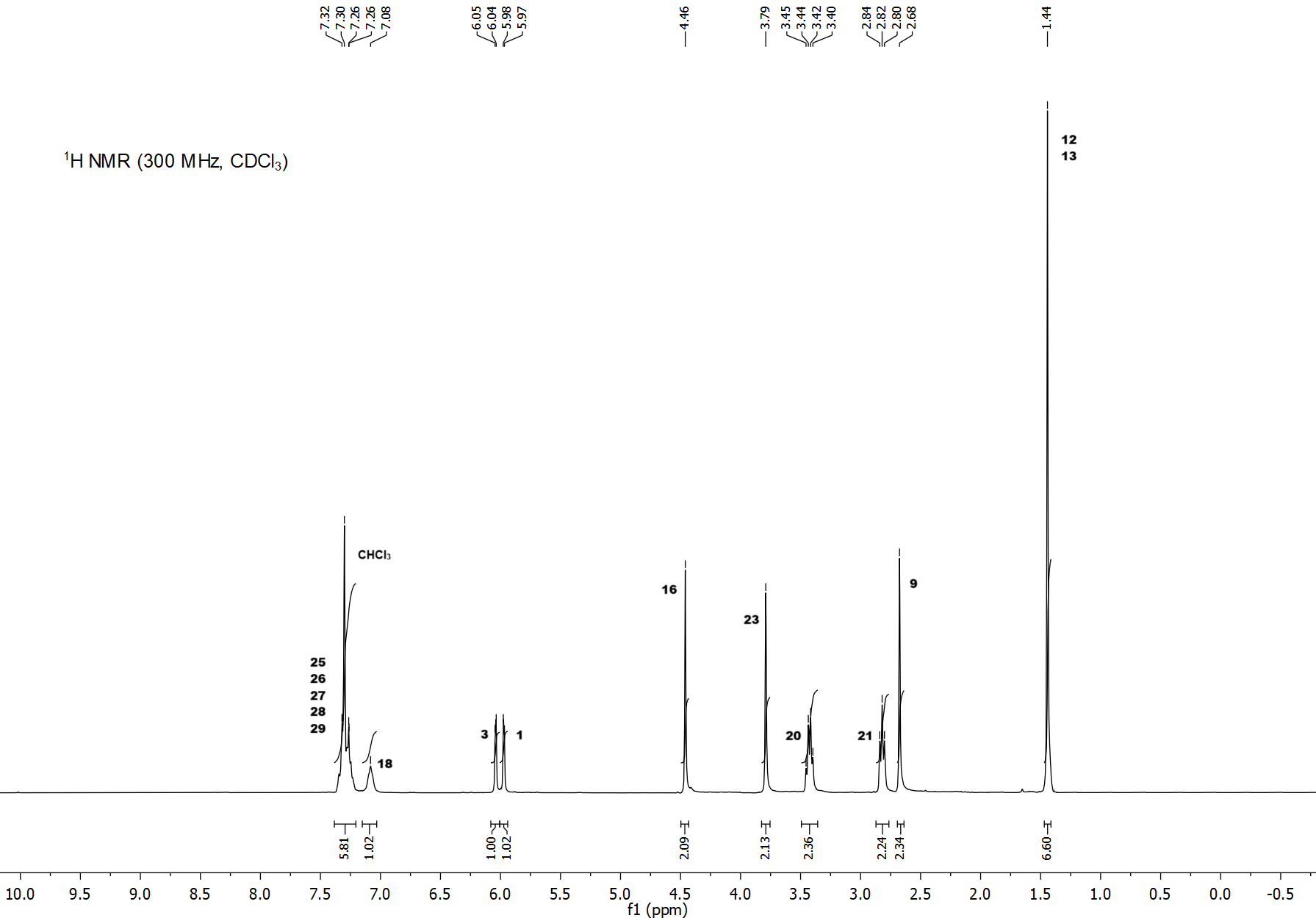


Figure S9. ^1^H NMR spectrum of 1c.


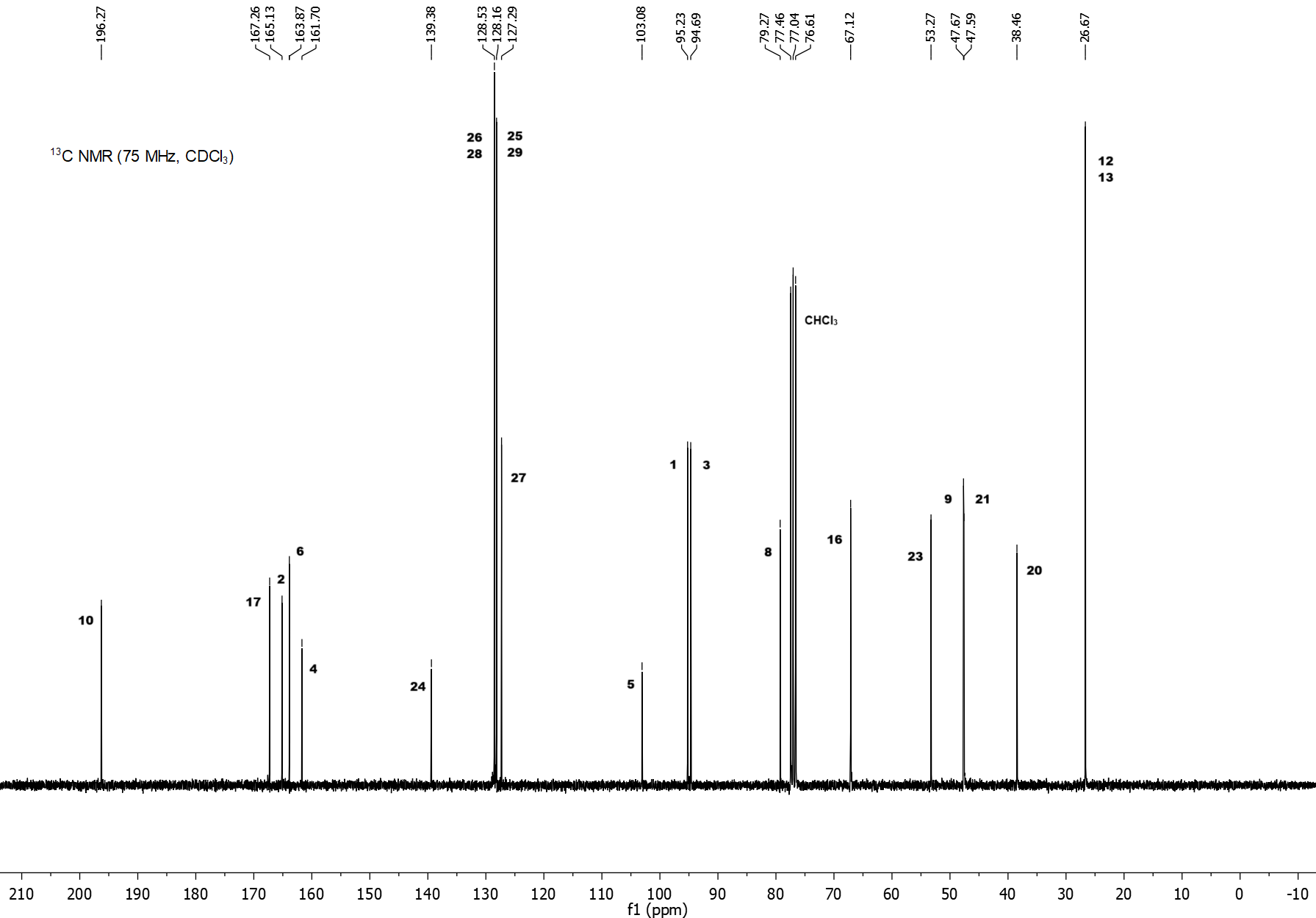


Figure S10. ^13^C NMR spectrum of 1c.

***
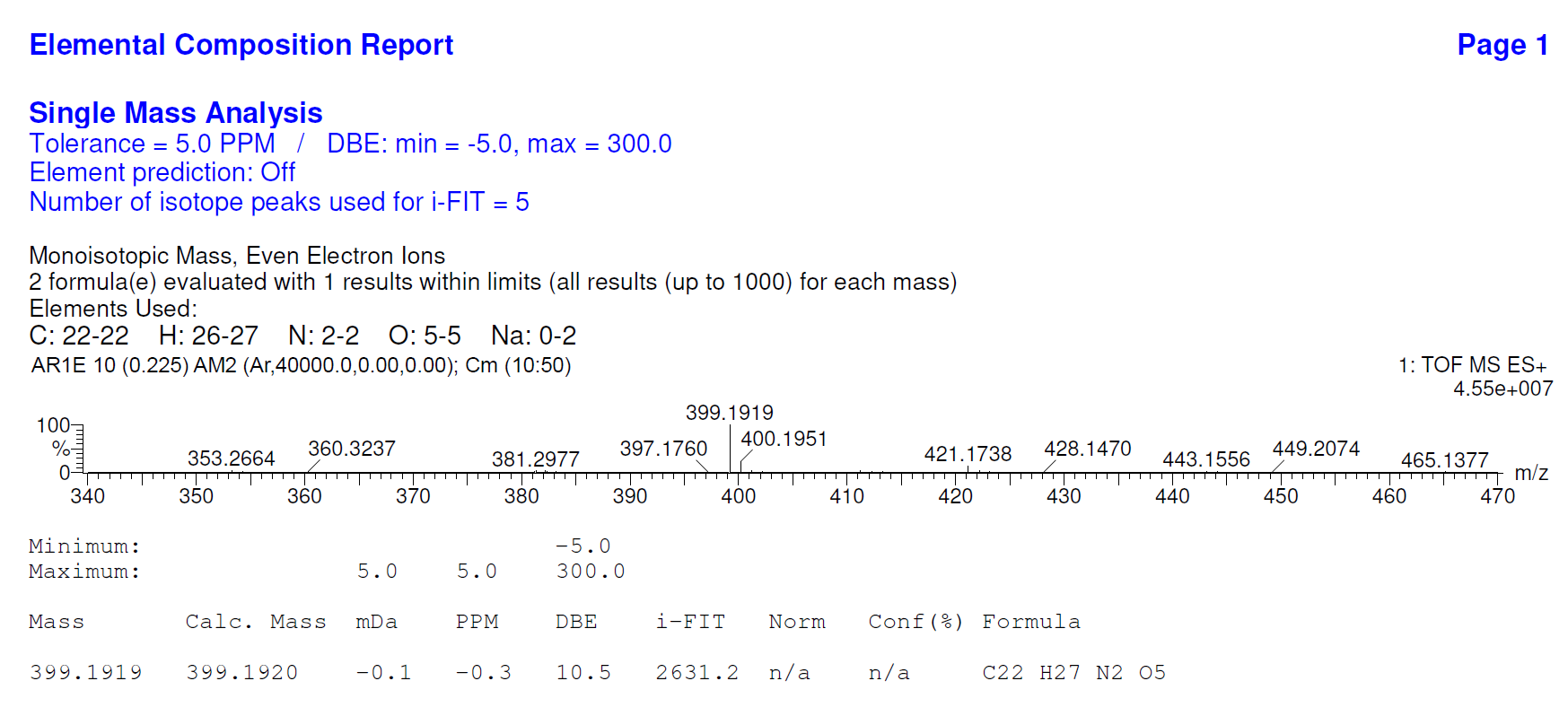
***

***
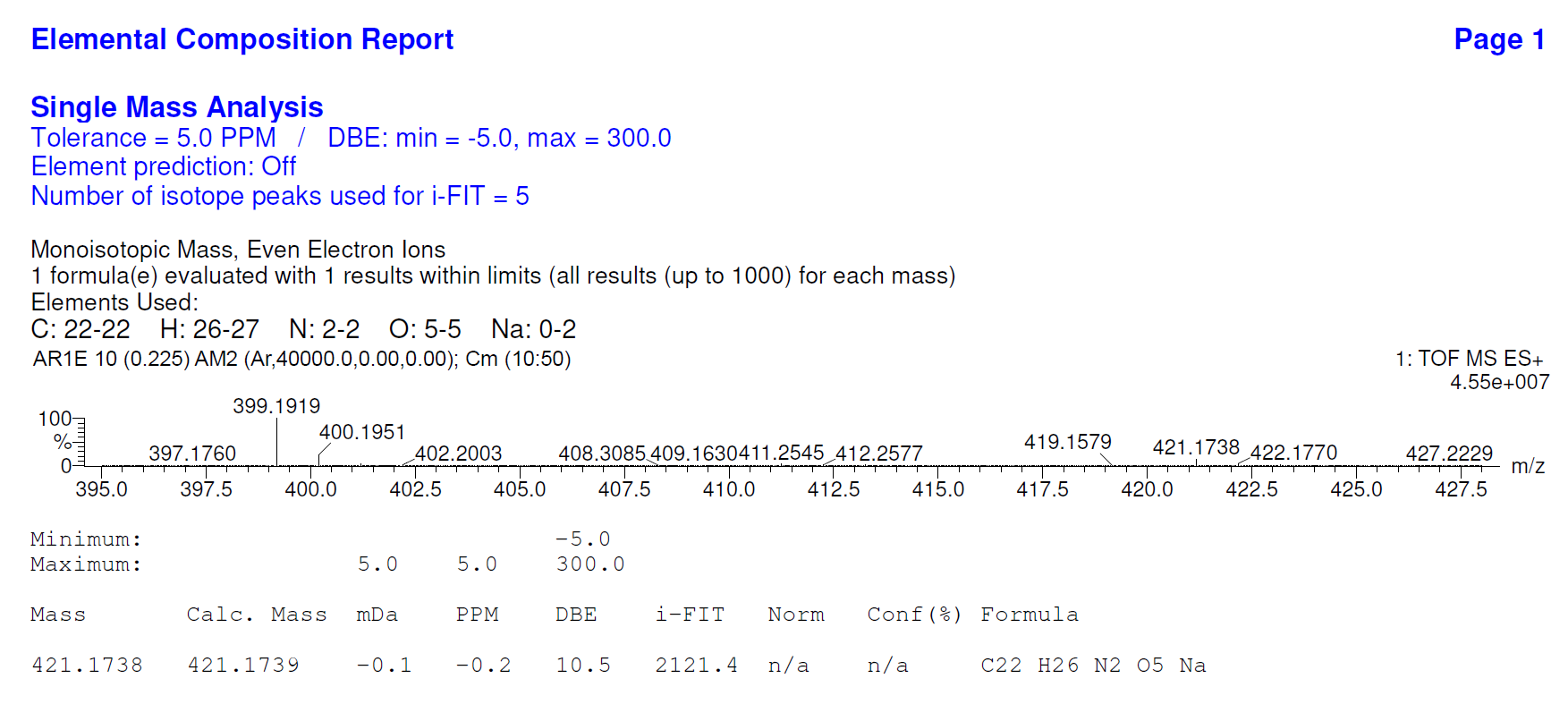
***

***
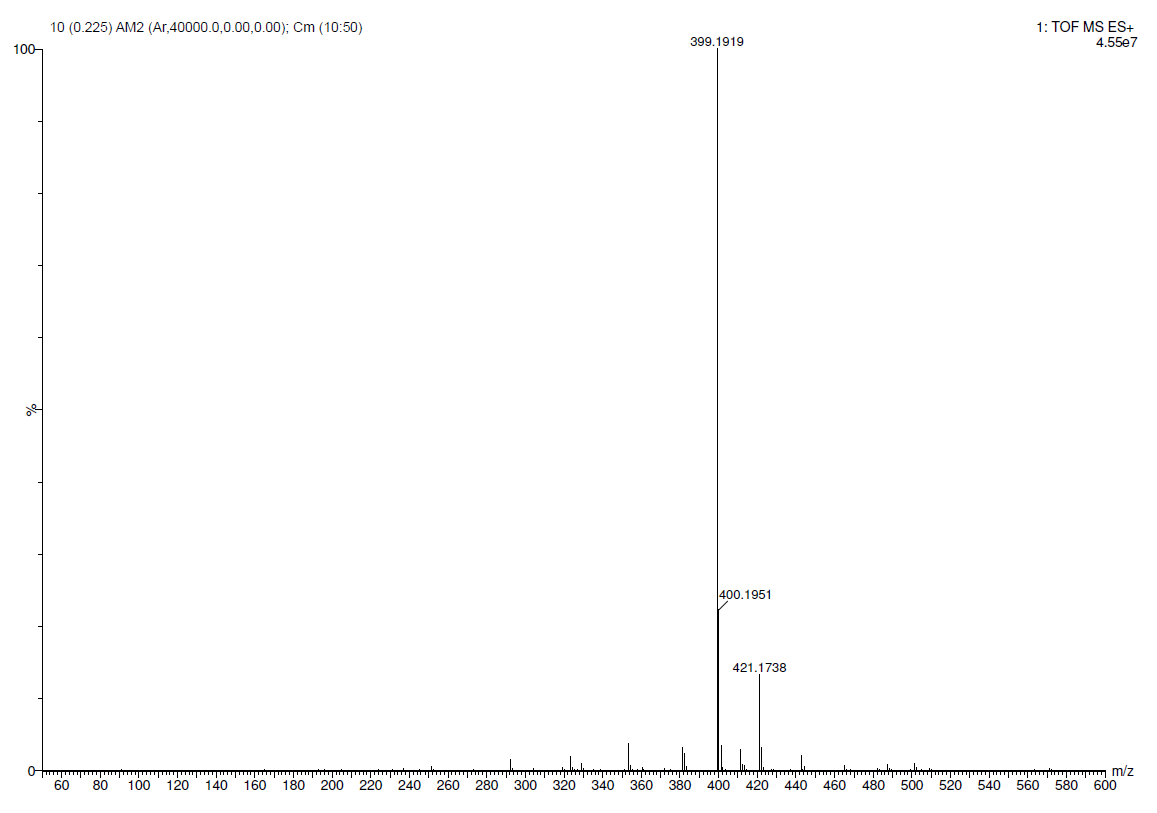
***

Figure S11. HRMS spectrum of 1c.

| 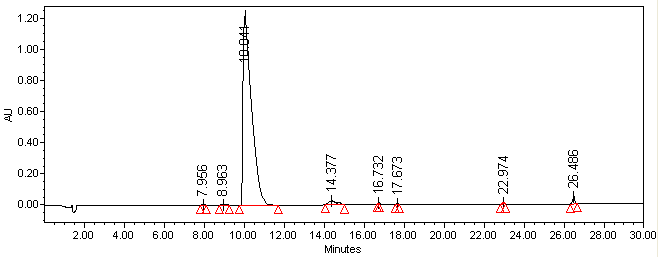 | **Retention time (min)** | **Area (%)** |
| --- | --- | --- |
|  | 7.956 | 0.03 |
|  | 8.963 | 0.22 |
|  | 10.041 | 96.59 |
|  | 14.377 | 1.87 |
|  | 16.732 | 0.03 |
|  | 17.673 | 0.11 |
|  | 22.974 | 0.19 |
|  | 26.486 | 0.95 |

Figure S12. HPLC chromatogram of 1c. Solvent A: 0.05% TFA in H_2_O. Solvent B: 0.05% TFA in MeCN. Method: linear-gradient mode, 30% B → 30% B (5 min) → 70% B (25 min). Total run time: 30 min. Flow rate: 1 mL/min. Detector wavelength: 220 nm.

***N-(3-Chlorobenzyl)-2-((5-hydroxy-2,2-dimethyl-4-oxochroman-7-yl)oxy)acetamide (1d)***


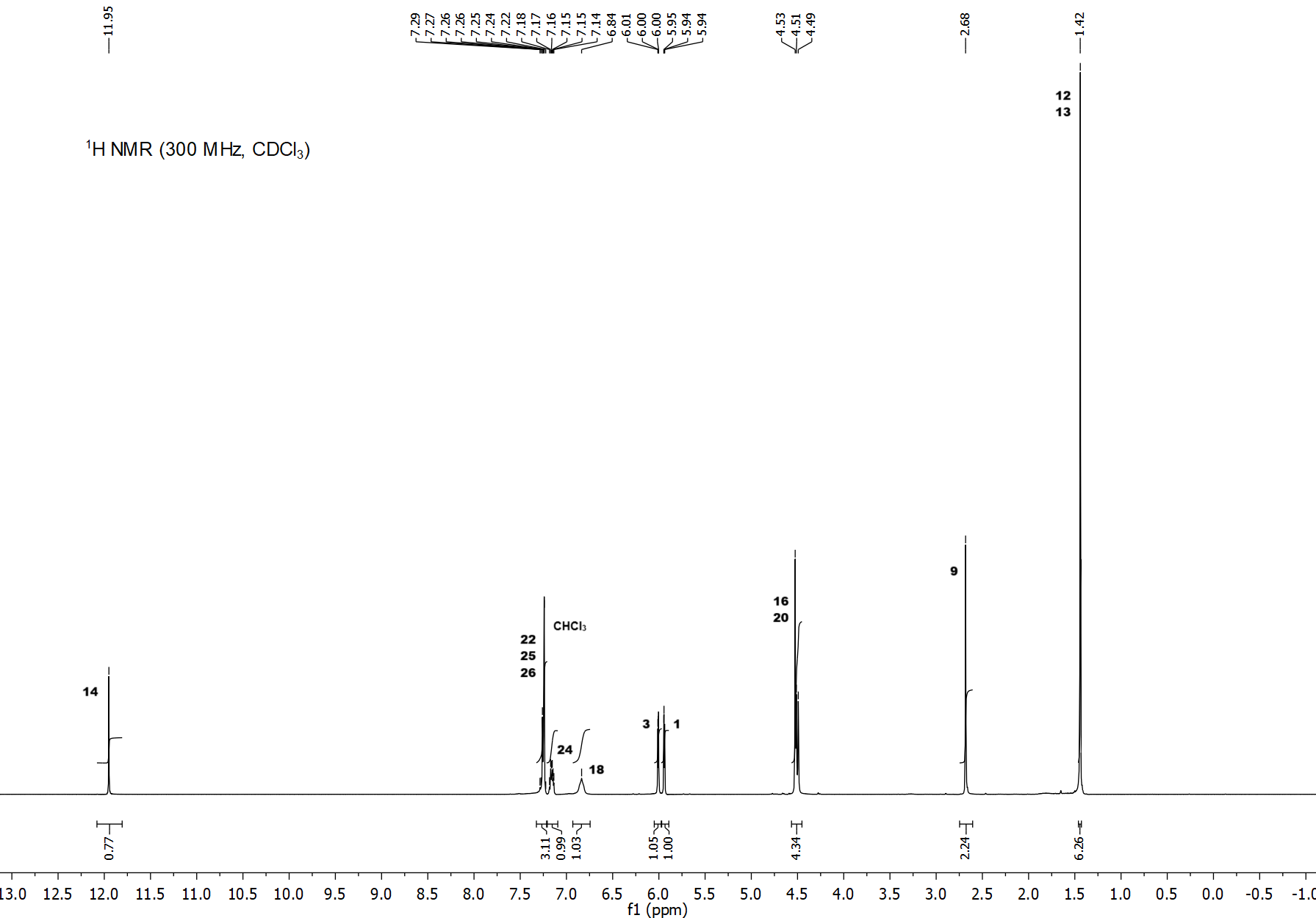


Figure S13. ^1^H NMR spectrum of 1d.


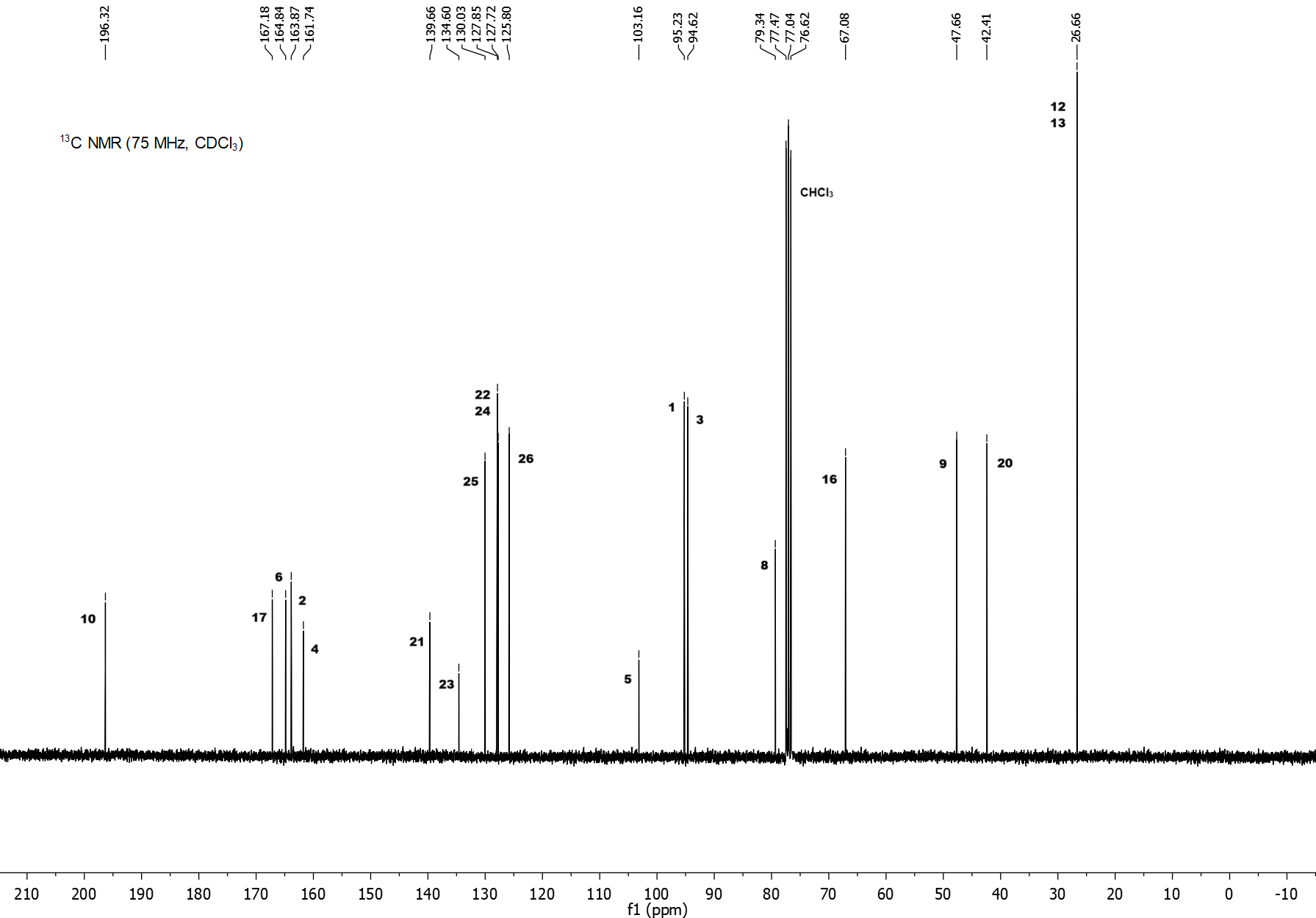


Figure S14. ^13^C NMR spectrum of 1d.


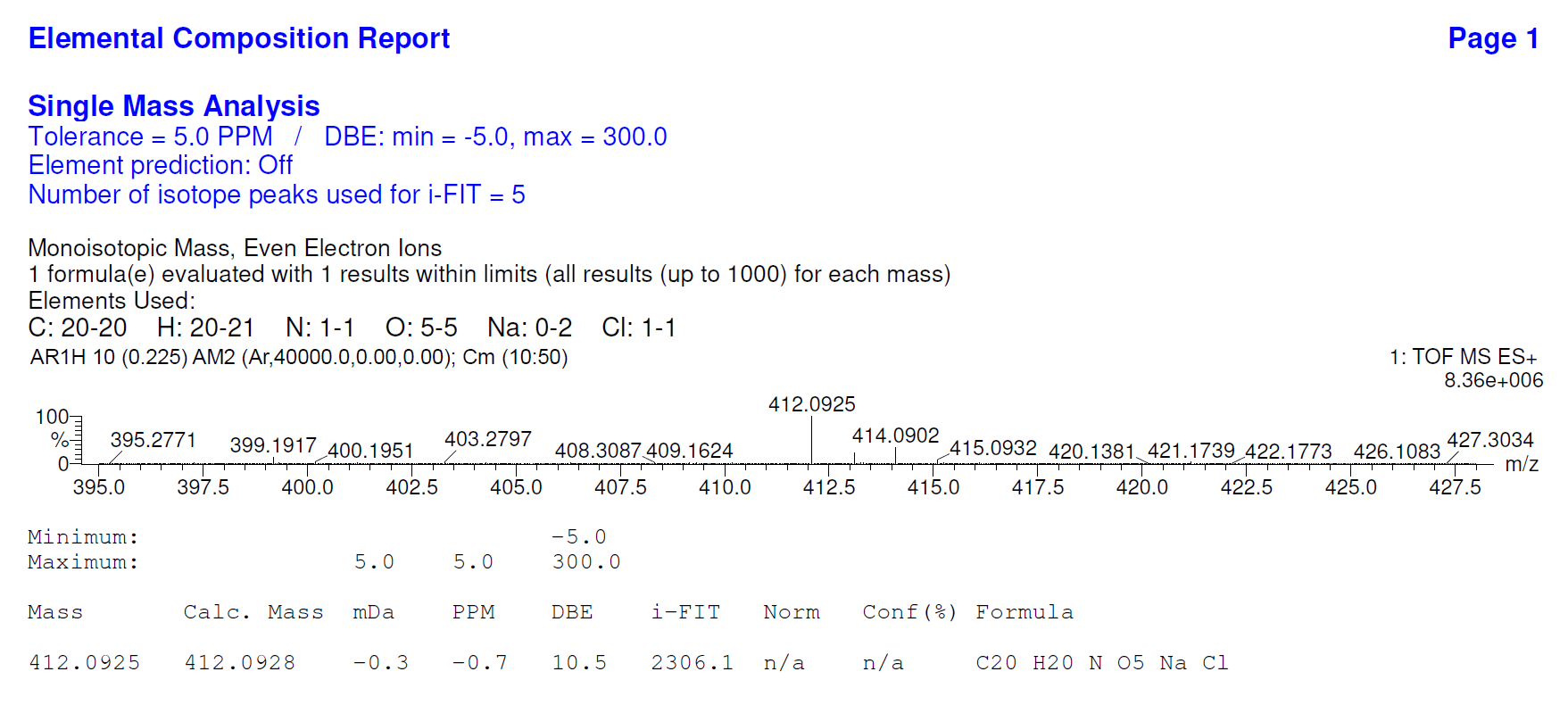


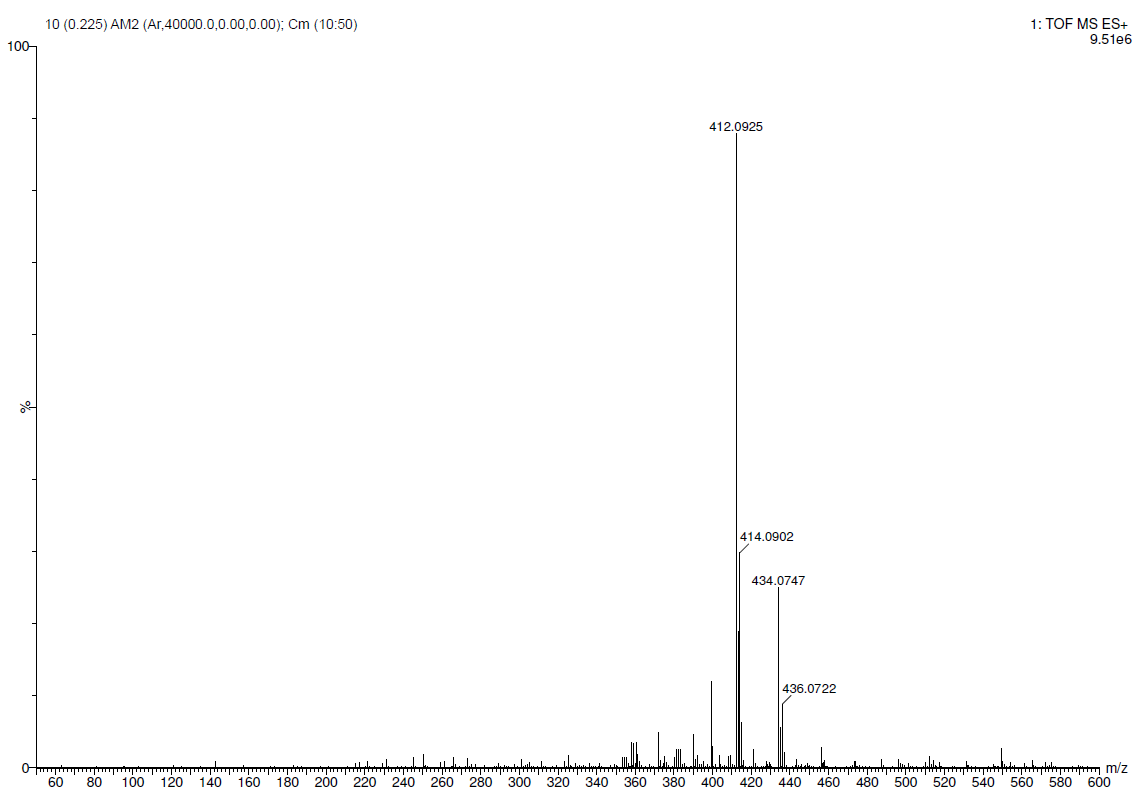


Figure S15. HRMS spectrum of 1d.

| 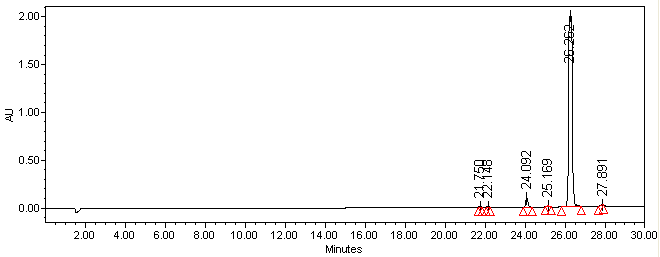 | **Retention time (min)** | **Area (%)** |
| --- | --- | --- |
|  | 21.750 | 0.15 |
|  | 22.148 | 0.17 |
|  | 24.092 | 2.70 |
|  | 25.169 | 0.35 |
|  | 26.262 | 96.49 |
|  | 27.891 | 0.15 |

Figure S16. HPLC chromatogram of 1d. Solvent A: 0.05% TFA in H_2_O. Solvent B: 0.05% TFA in MeCN. Method: linear-gradient mode, 30% B → 30% B (5 min) → 70% B (25 min). Total run time: 30 min. Flow rate: 1 mL/min. Detector wavelength: 220 nm.

***2-((5-Hydroxy-2,2-dimethyl-4-oxochroman-7-yl)oxy)-N-(naphthalen-1-ylmethyl)acetamide (1e)***


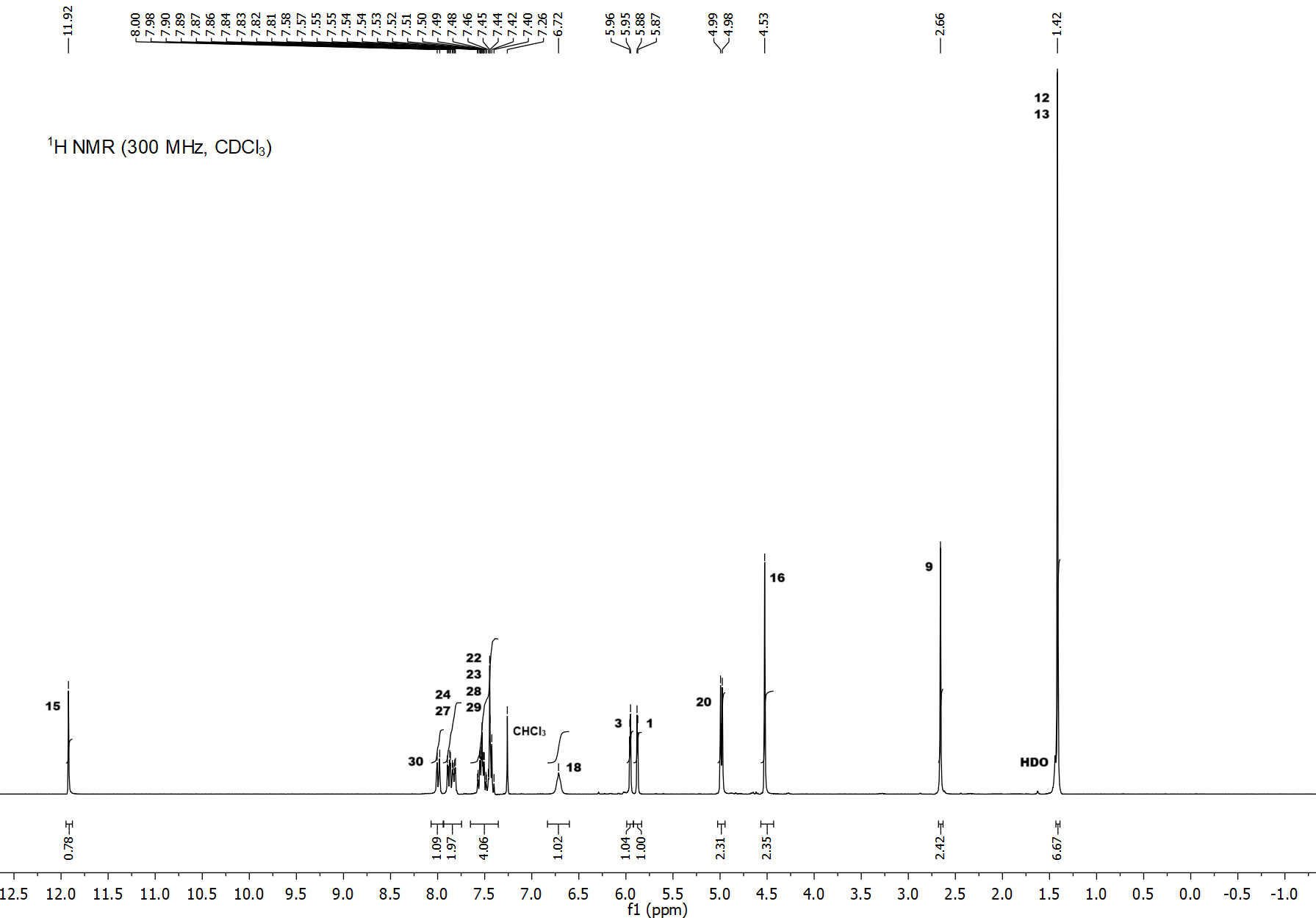


Figure S17. ^1^H NMR spectrum of 1e.


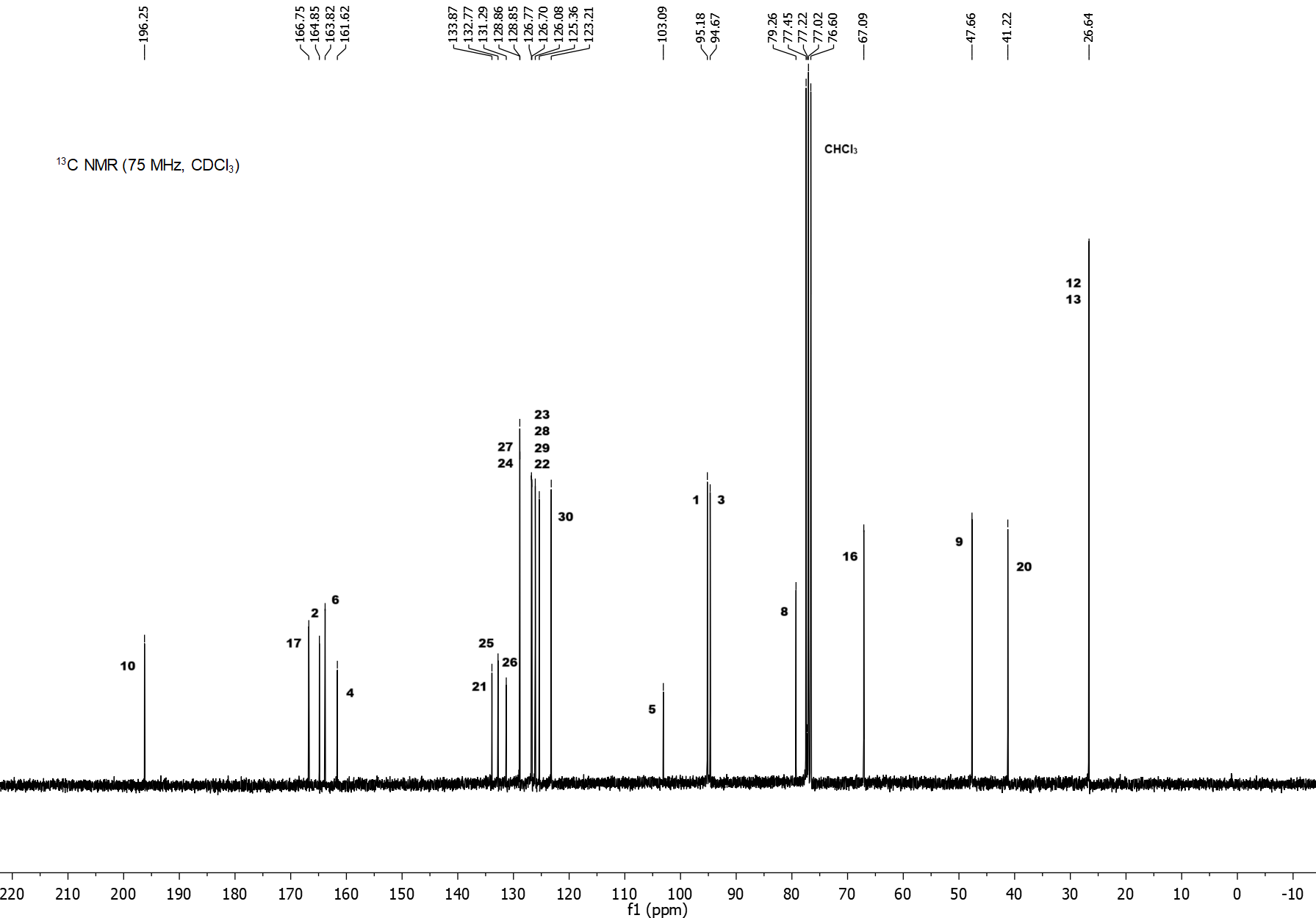


Figure S18. ^13^C NMR spectrum of 1e.


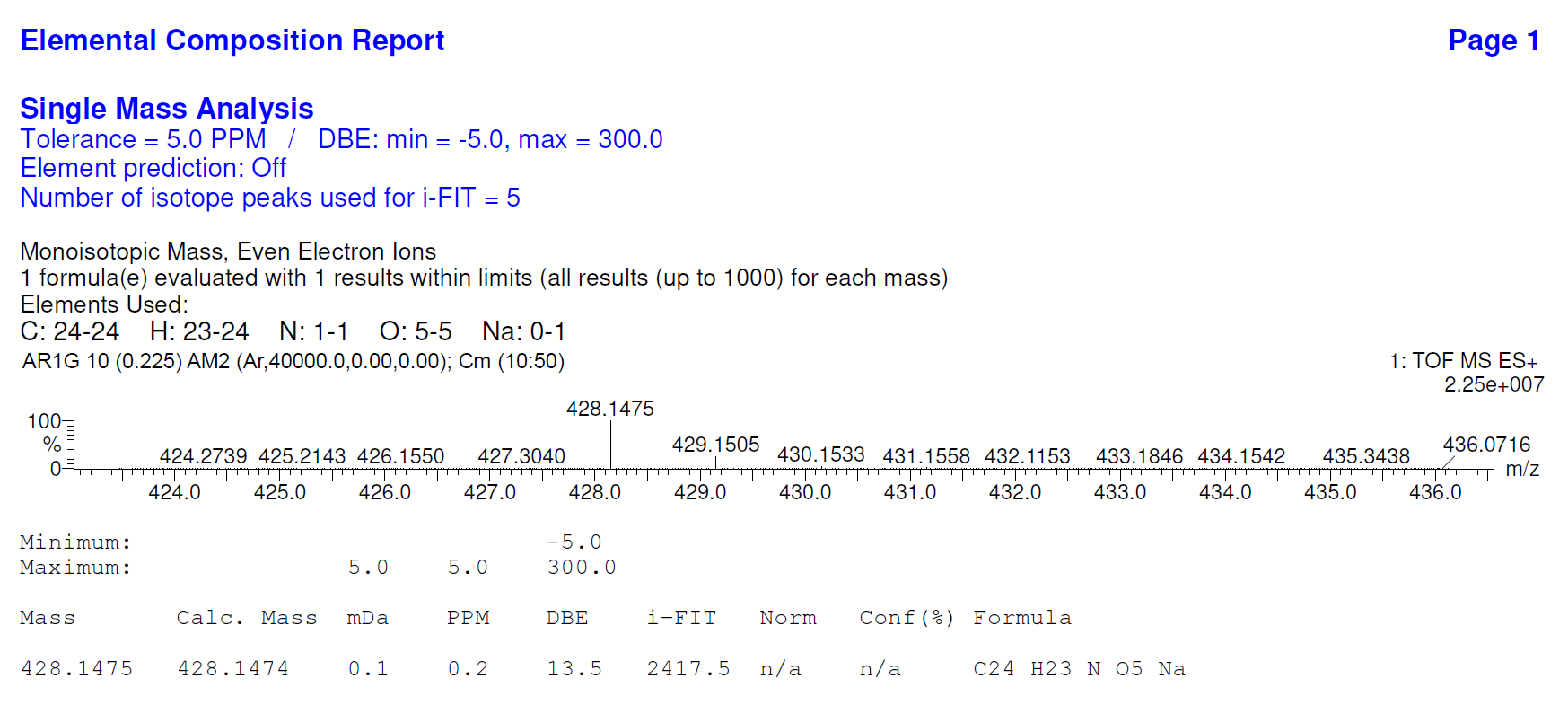


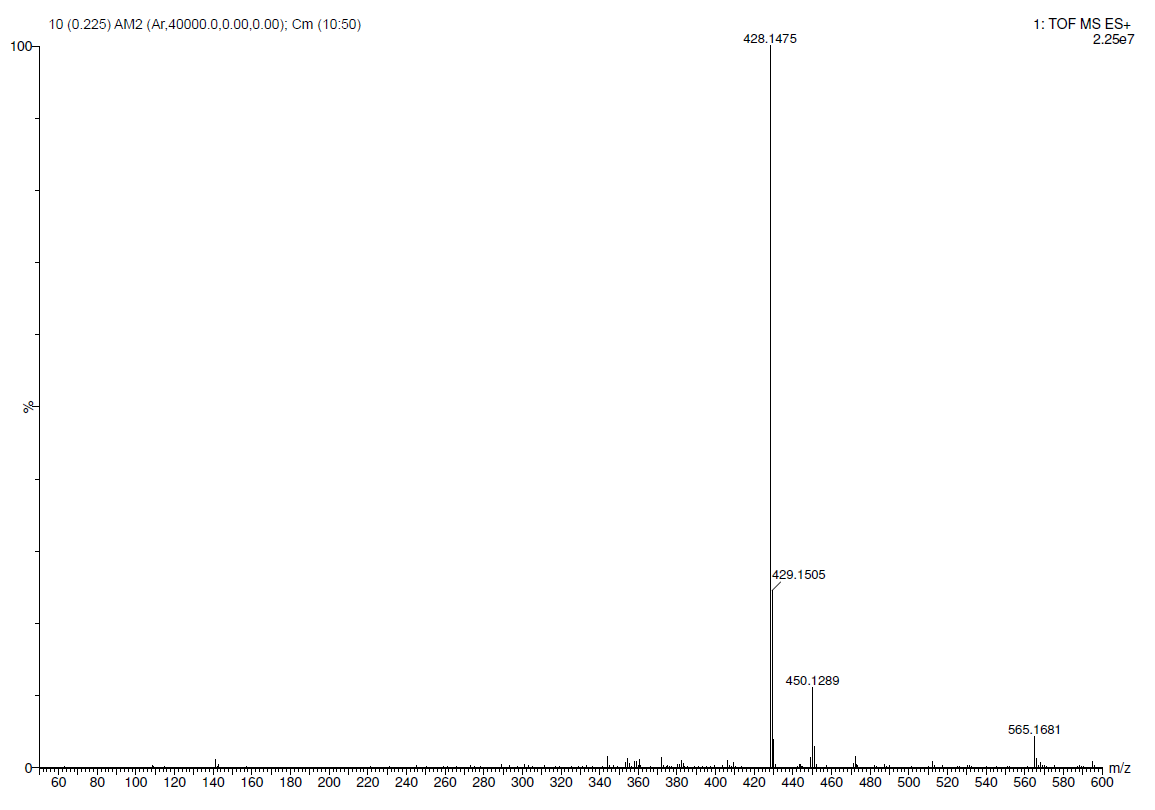


Figure S19. HRMS spectrum of 1e.

| 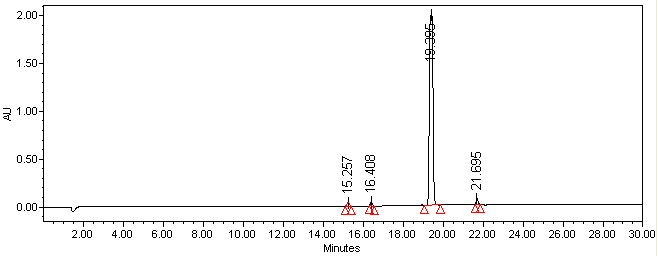 | **Retention time (min)** | **Area (%)** |
| --- | --- | --- |
|  | 15.257 | 1.27 |
|  | 16.408 | 1.01 |
|  | 19.395 | 96.18 |
|  | 21.695 | 1.54 |
|  |  |  |

Figure S20. HPLC chromatogram of 1e. Solvent A: 0.05% TFA in H_2_O. Solvent B: 0.05% TFA in MeCN. Method: linear-gradient mode, 40% B → 100% B (20 min). Total run time: 30 min. Flow rate: 1 mL/min. Detector wavelength: 220 nm.

***5,7-Dihydroxy-2,2-dimethylchroman-4-one (2)***


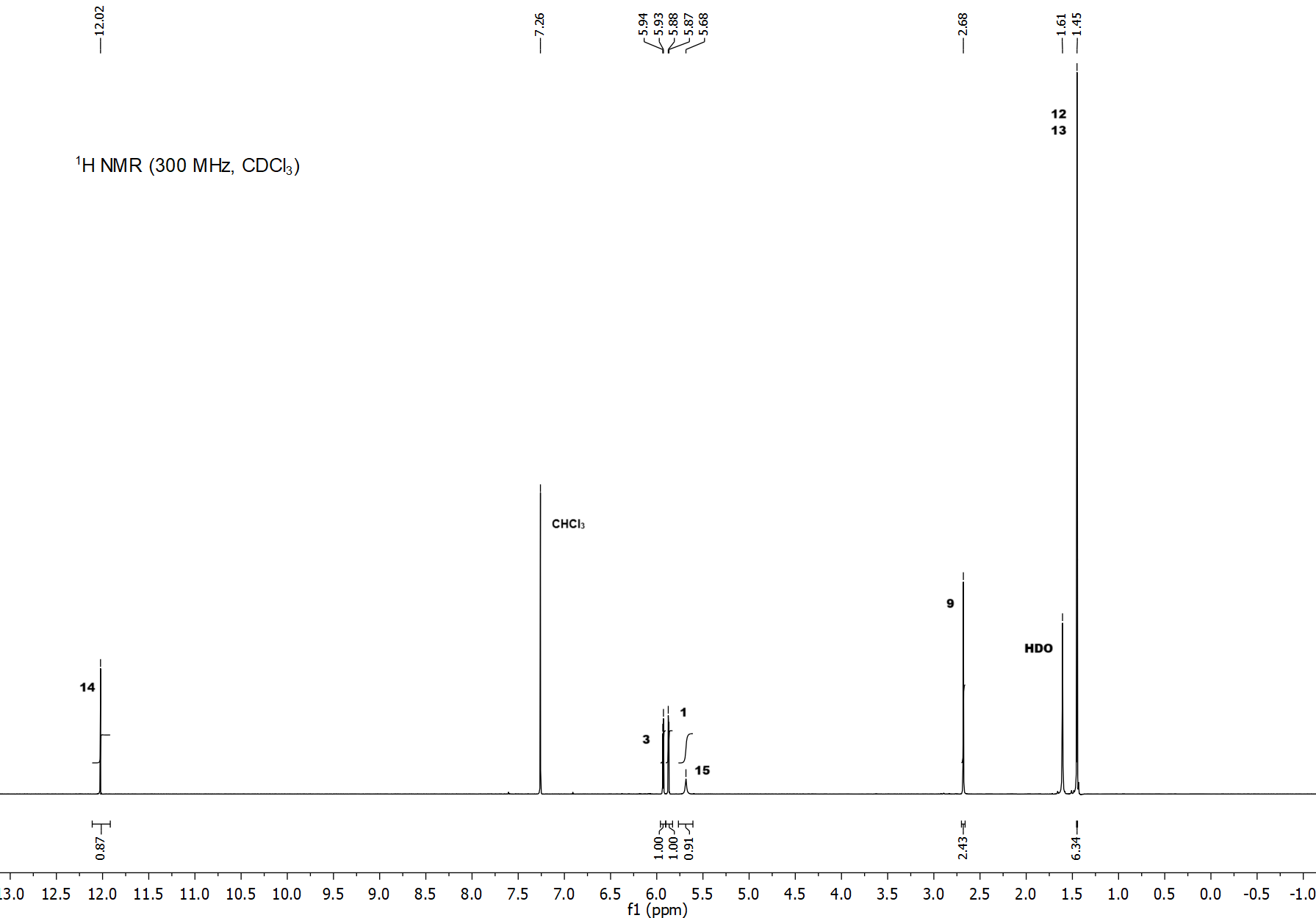


Figure S21. ^1^H NMR spectrum of 2.


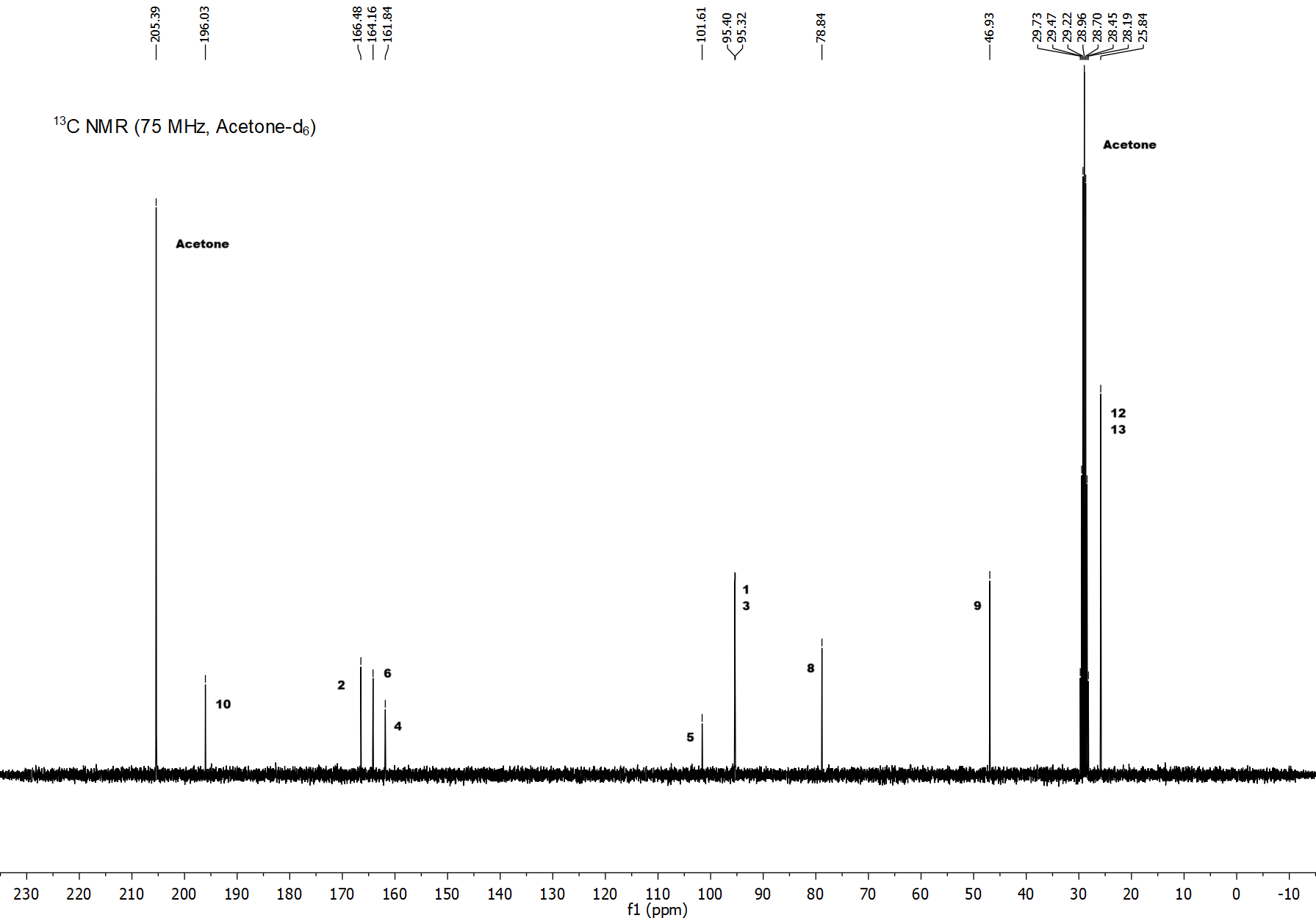


Figure S22. ^13^C NMR spectrum of 2.

***Ethyl 2-((5-hydroxy-2,2-dimethyl-4-oxochroman-7-yl) oxy) acetate (3)***


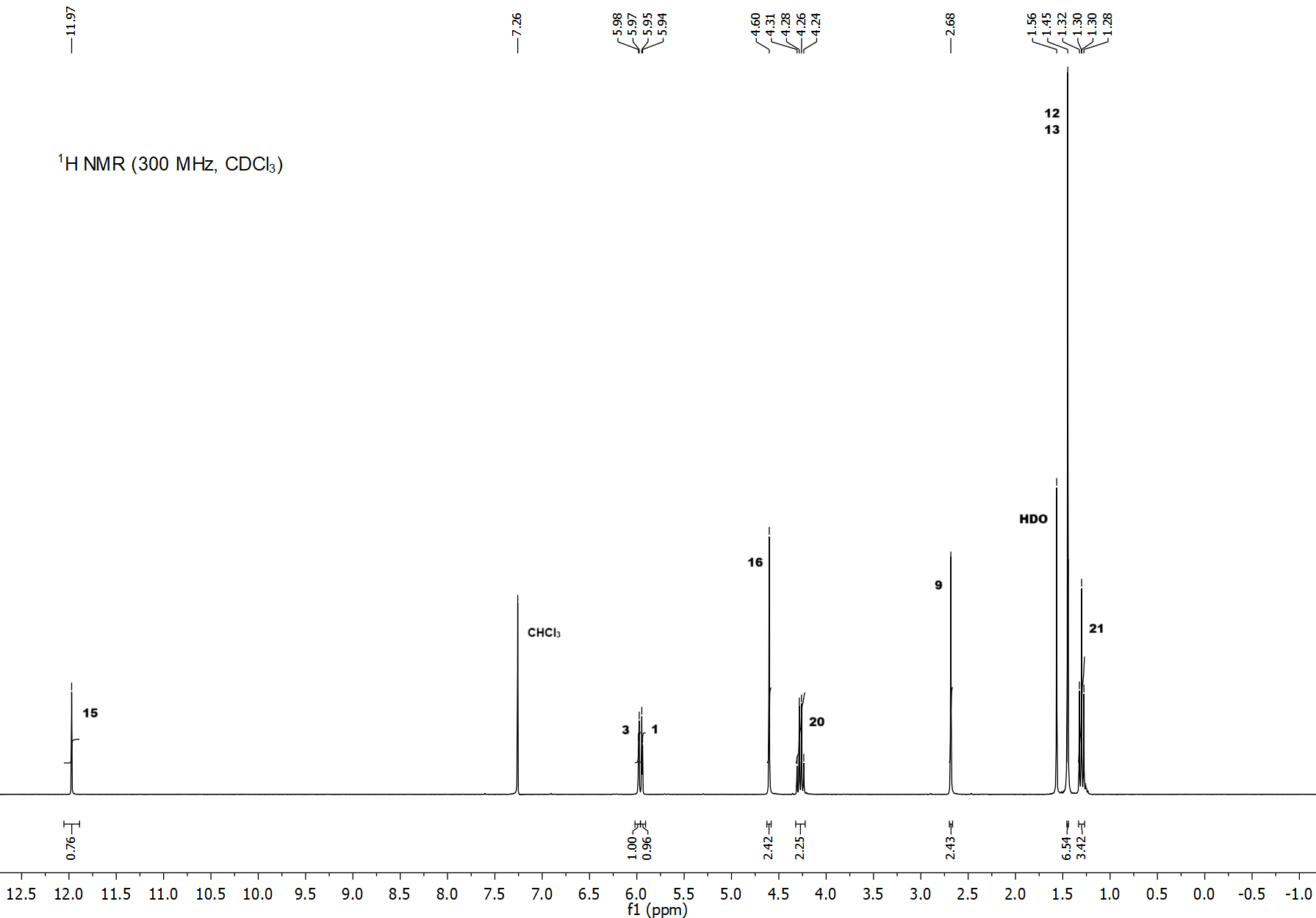


Figure S23. ^1^H NMR spectrum of 3.


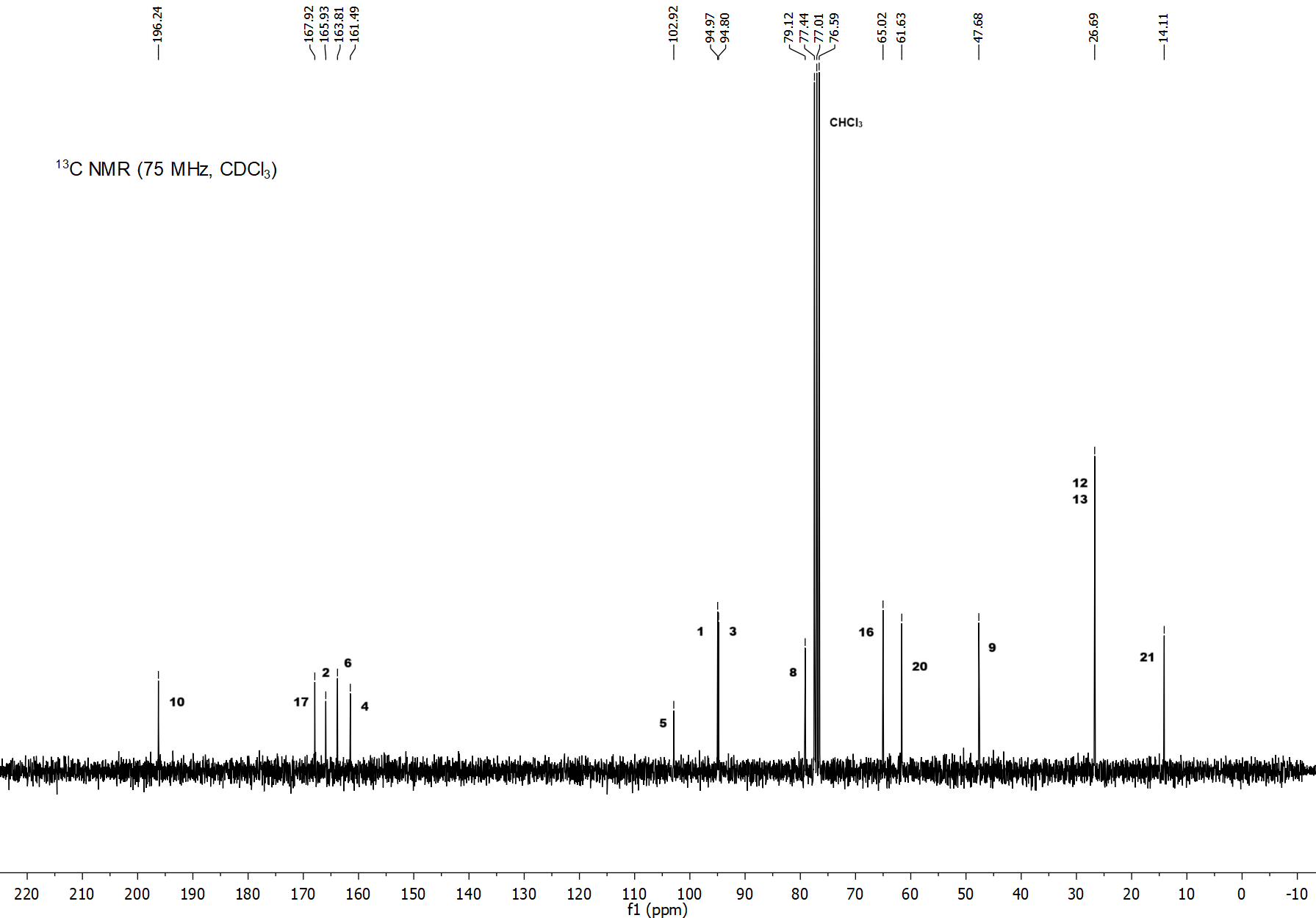


Figure S24. ^13^C NMR spectrum of 3.

*2-((5-Hydroxy-2,2-dimethyl-4-oxochroman-7-yl) oxy) acetic acid (4)*


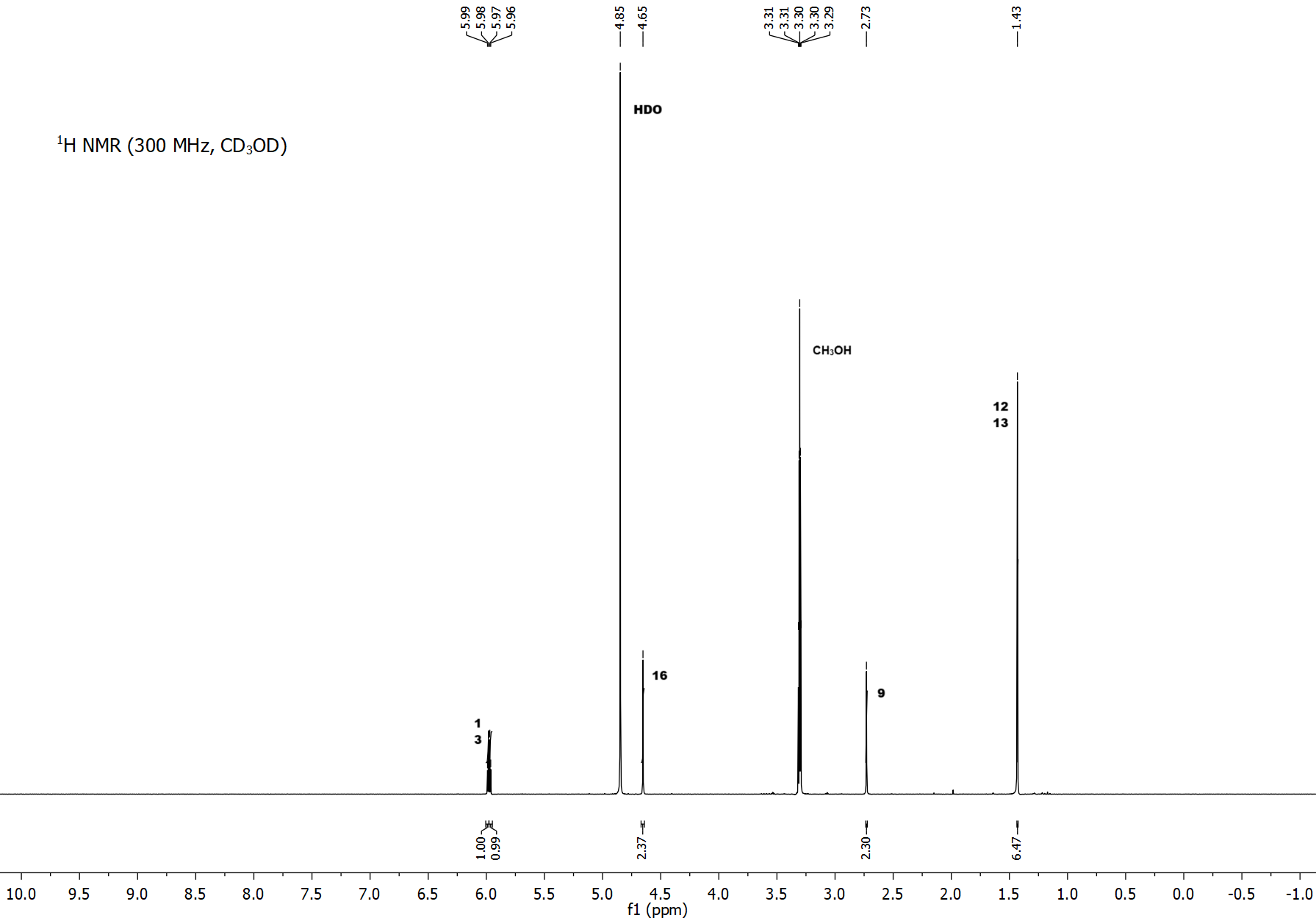


Figure S25. ^1^H NMR spectrum of 4.


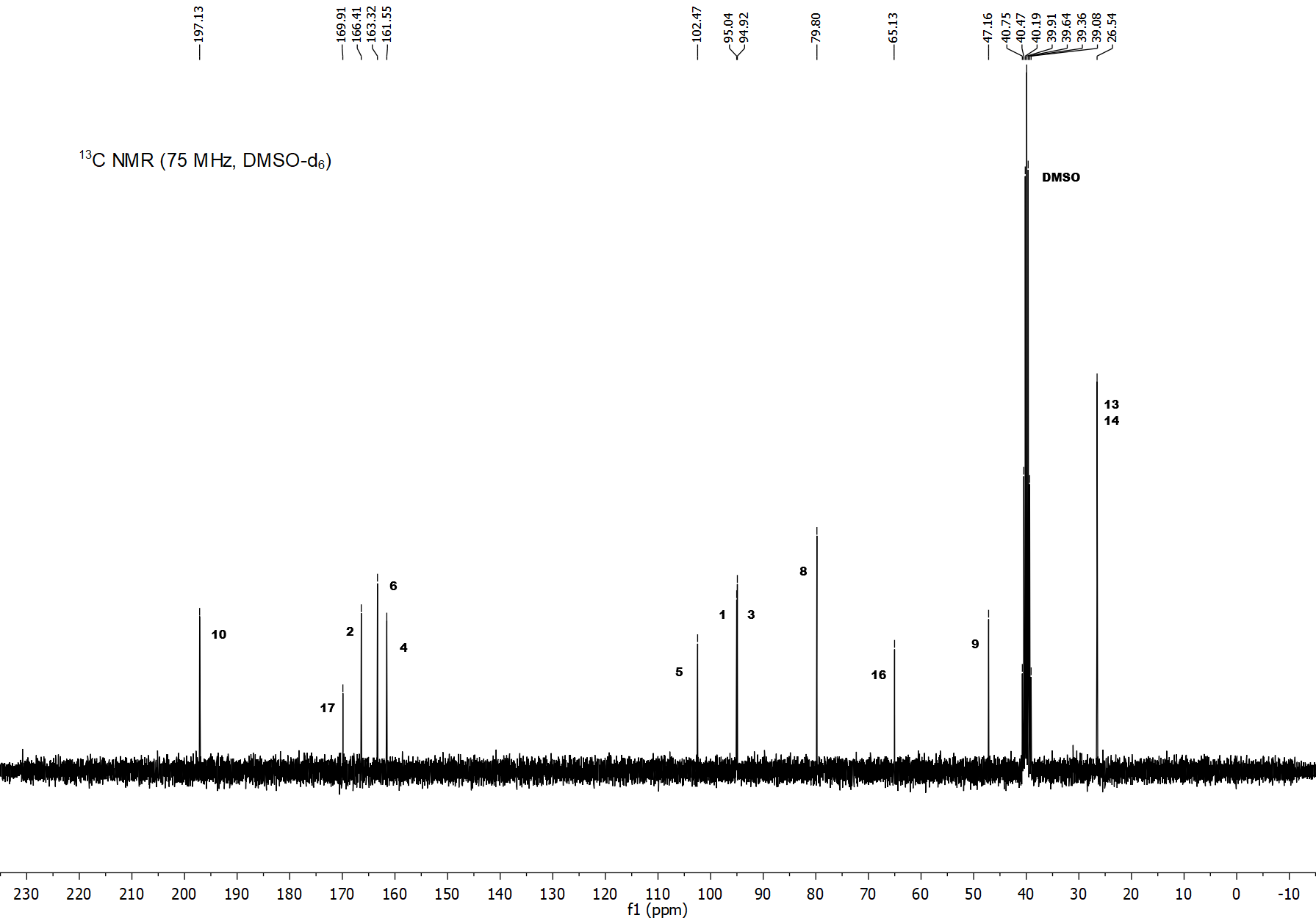


Figure S26. ^13^C NMR spectrum of 4.

*N^1^-(Naphthalen-1-ylmethyl)ethane-1,2-diamine (5)*


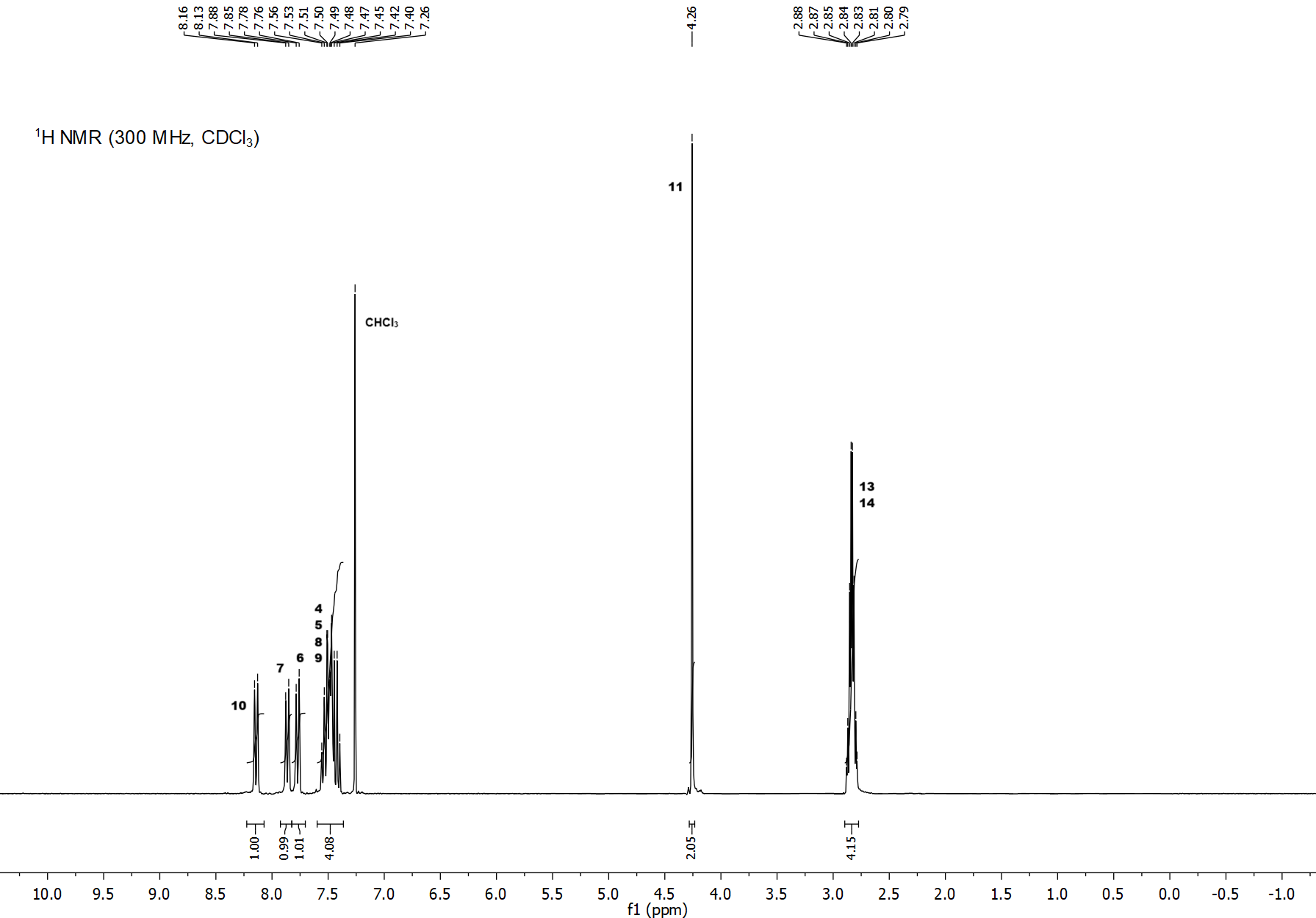


Figure S27. ^1^H NMR spectrum of 5.


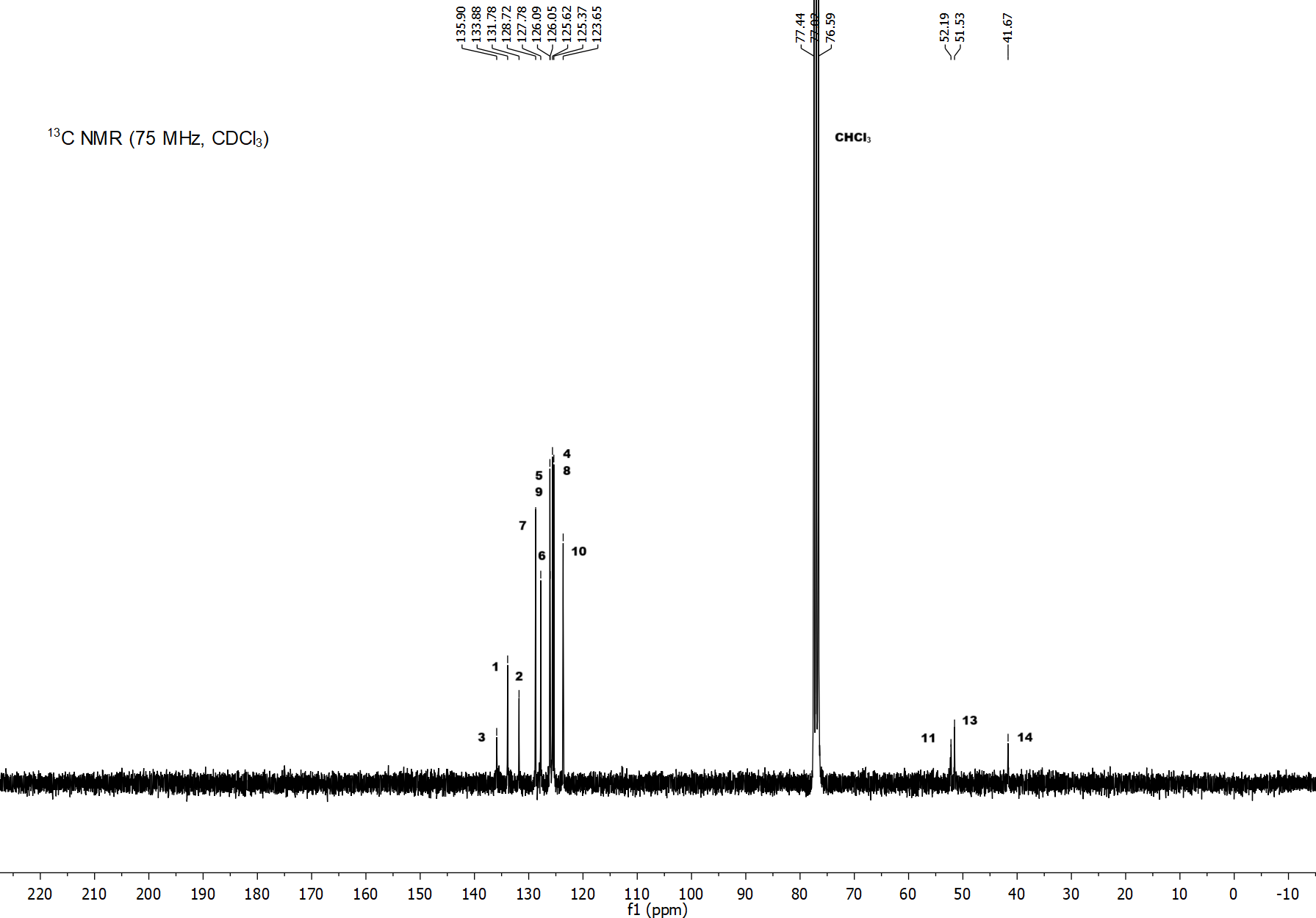


Figure S28. ^13^C NMR spectrum of 5.

***Naphthalen-1-ylmethanamine (6)***


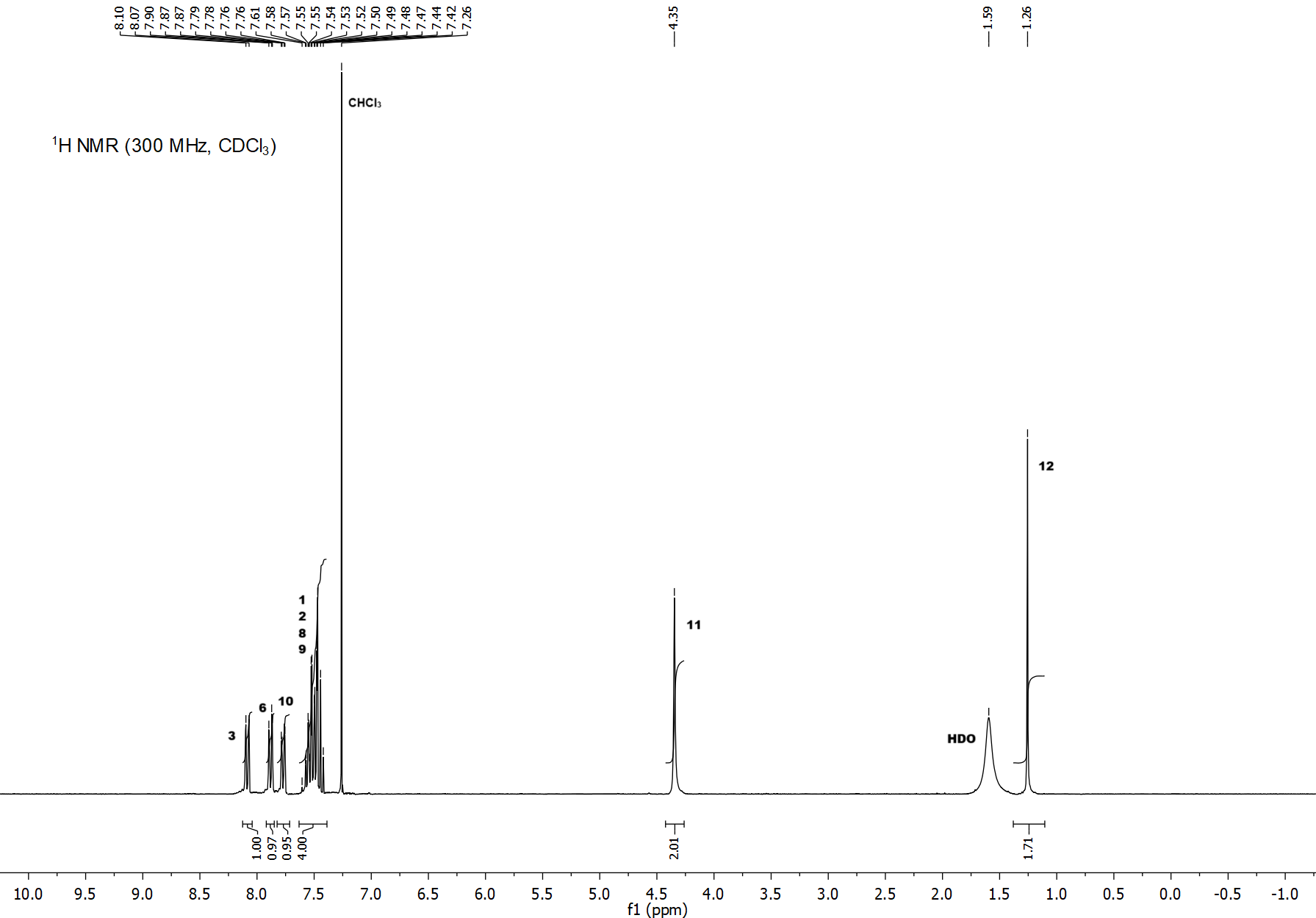


Figure S29. ^1^H NMR spectrum of 6.

**
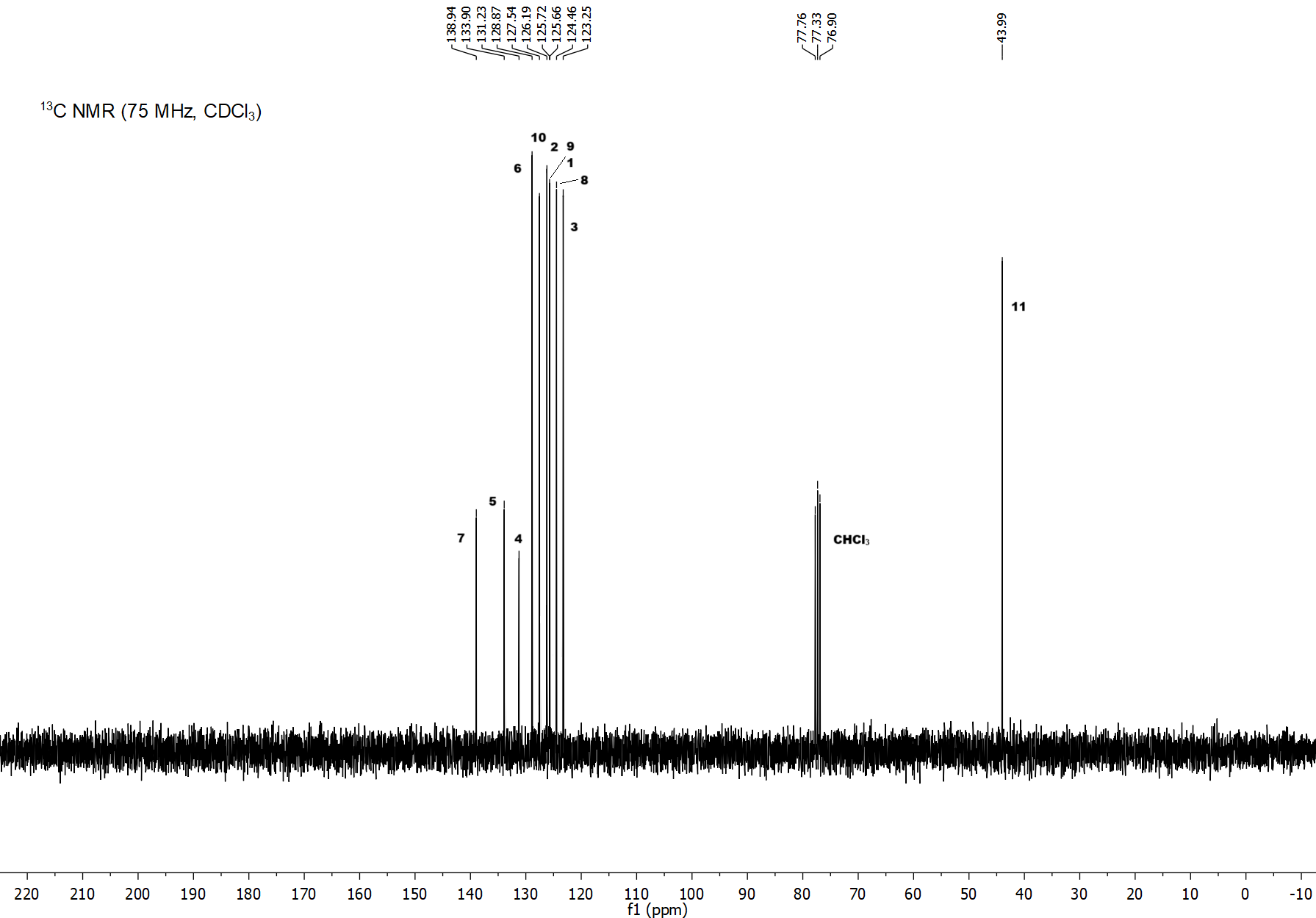
**

Figure S30. ^13^C NMR spectrum of 6.

***tert*-Butyl (2-((naphthalen-1-ylmethyl)amino)ethyl)carbamate (7)**


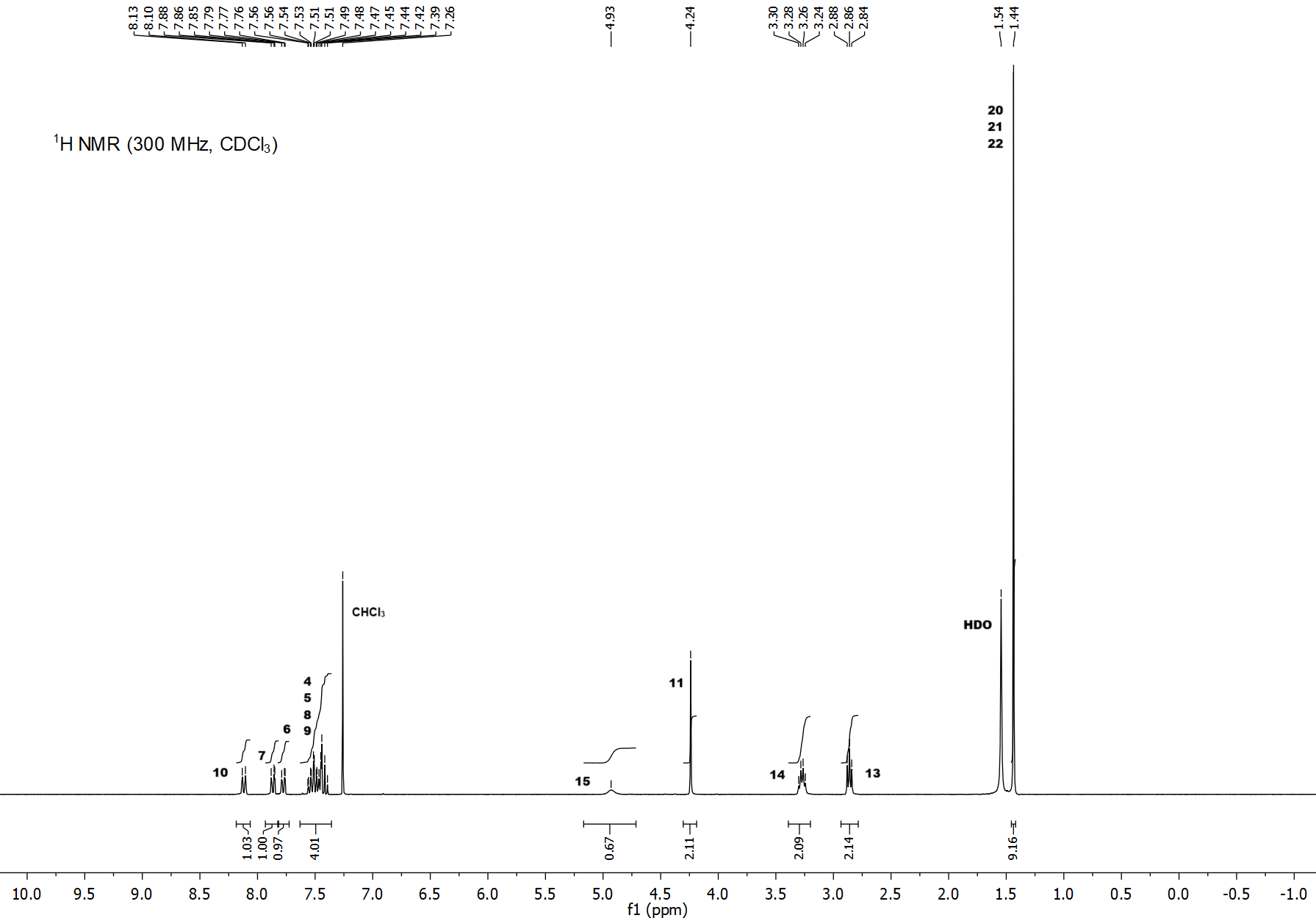


Figure S31. ^1^H NMR spectrum of 7.


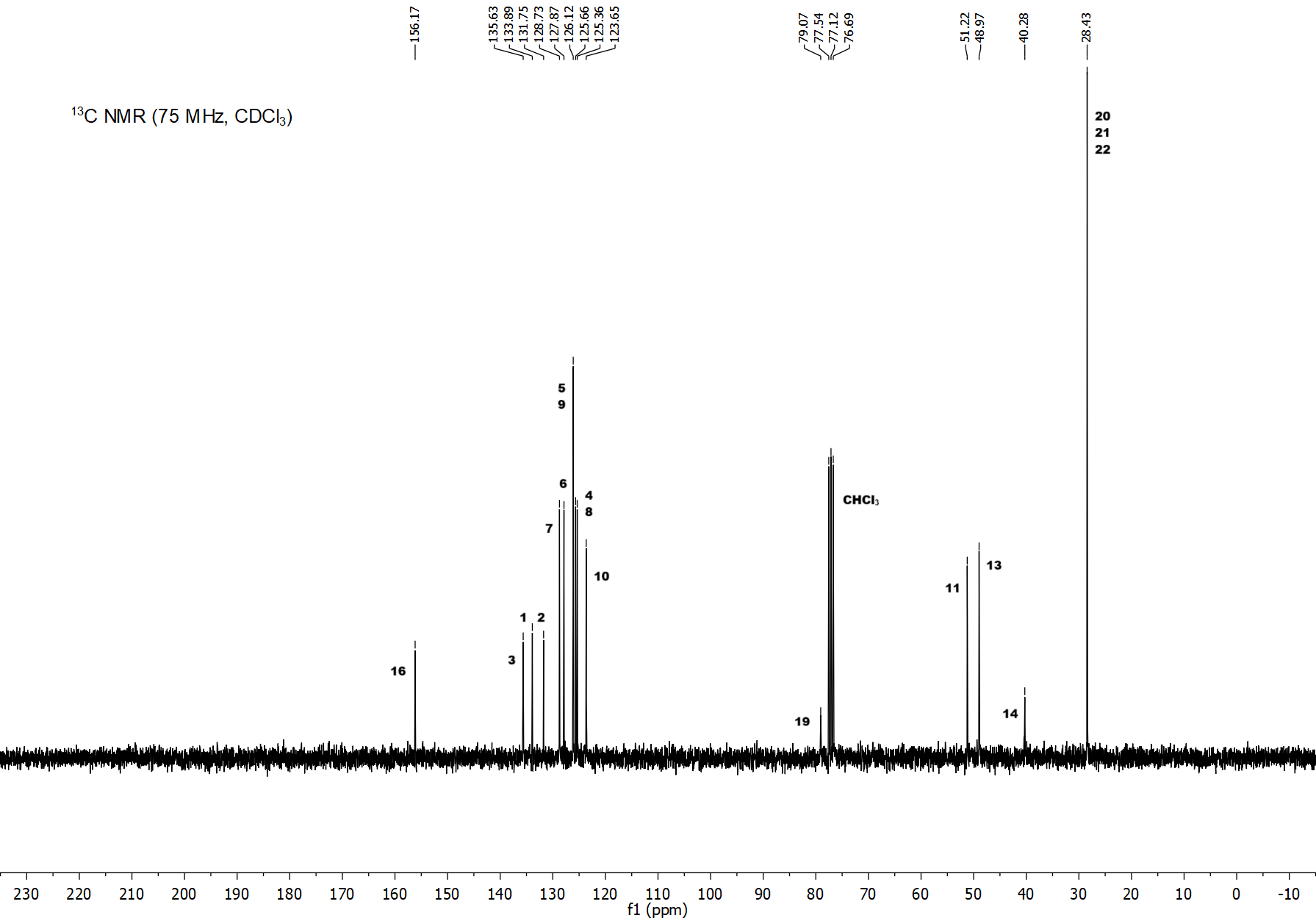


Figure S32. ^13^C NMR spectrum of 7.

**Crystallographic analysis of 4**

**Table S1.** Bond lengths [Å] and angles [°] for **4**.

| **Bond** | **Measurement [Å]** |
| --- | --- |
| O(4)-C(5) | 1.365(2) |
| O(4)-C(12) | 1.417(2) |
| C(2)-C(1) | 1.411(3) |
| C(2)-C(3) | 1.412(2) |
| C(2)-C(7) | 1.453(2) |
| O(3)-C(3) | 1.354(2) |
| O(3)-C(9) | 1.469(2) |
| O(5)-C(13) | 1.212(2) |
| O(2)-C(7) | 1.234(2) |
| C(5)-C(6) | 1.385(3) |
| C(5)-C(4) | 1.393(2) |
| O(1)-C(1) | 1.344(2) |
| O(1)-H(1) | 0.93(3) |
| C(4)-C(3) | 1.377(2) |
| C(4)-H(4) | 0.9300 |
| O(6)-C(13) | 1.306(2) |
| O(6)-H(6A) | 0.94(3) |
| C(1)-C(6) | 1.389(3) |
| C(6)-H(6) | 0.9300 |
| C(8)-C(7) | 1.498(3) |
| C(8)-C(9) | 1.518(3) |
| C(8)-H(8A) | 0.9700 |
| C(8)-H(8B) | 0.9700 |
| C(12)-C(13) | 1.501(3) |
| C(12)-H(12A) | 0.9700 |
| C(12)-H(12B) | 0.9700 |
| C(9)-C(10) | 1.516(3) |
| C(9)-C(11) | 1.517(3) |
| C(10)-H(10A) | 0.9600 |
| C(10)-H(10B) | 0.9600 |
| C(10)-H(10C) | 0.9600 |
| C(11)-H(11A) | 0.9600 |
| C(11)-H(11B) | 0.9600 |
| C(11)-H(11C) | 0.9600 |
| **Angle** | **Measurement [°]** |
| C(5)-O(4)-C(12) | 116.96(14) |
| C(1)-C(2)-C(3) | 118.10(16) |
| C(1)-C(2)-C(7) | 121.53(16) |
| C(3)-C(2)-C(7) | 120.26(16) |
| C(3)-O(3)-C(9) | 117.93(14) |
| O(4)-C(5)-C(6) | 123.61(16) |
| O(4)-C(5)-C(4) | 114.49(16) |
| C(6)-C(5)-C(4) | 121.89(16) |
| C(1)-O(1)-H(1) | 107.7(18) |
| C(3)-C(4)-C(5) | 119.10(17) |
| C(3)-C(4)-H(4) | 120.4 |
| C(5)-C(4)-H(4) | 120.4 |
| C(13)-O(6)-H(6A) | 107.1(18) |
| O(3)-C(3)-C(4) | 116.93(16) |
| O(3)-C(3)-C(2) | 122.00(16) |
| C(4)-C(3)-C(2) | 121.07(16) |
| O(1)-C(1)-C(6) | 118.20(17) |
| O(1)-C(1)-C(2) | 120.72(16) |
| C(6)-C(1)-C(2) | 121.08(17) |
| C(5)-C(6)-C(1) | 118.69(17) |
| C(5)-C(6)-H(6) | 120.7 |
| C(1)-C(6)-H(6) | 120.7 |
| C(7)-C(8)-C(9) | 112.58(15) |
| C(7)-C(8)-H(8A) | 109.1 |
| C(9)-C(8)-H(8A) | 109.1 |
| C(7)-C(8)-H(8B) | 109.1 |
| C(9)-C(8)-H(8B) | 109.1 |
| H(8A)-C(8)-H(8B) | 107.8 |
| O(2)-C(7)-C(2) | 122.61(18) |
| O(2)-C(7)-C(8) | 122.21(17) |
| C(2)-C(7)-C(8) | 115.15(16) |
| O(4)-C(12)-C(13) | 108.48(15) |
| O(4)-C(12)-H(12A) | 110.0 |
| C(13)-C(12)-H(12A) | 110.0 |
| O(4)-C(12)-H(12B) | 110.0 |
| C(13)-C(12)-H(12B) | 110.0 |
| H(12A)-C(12)-H(12B) | 108.4 |
| O(5)-C(13)-O(6) | 124.98(18) |
| O(5)-C(13)-C(12) | 123.78(17) |
| O(6)-C(13)-C(12) | 111.23(16) |
| O(3)-C(9)-C(10) | 103.96(17) |
| O(3)-C(9)-C(11) | 109.05(16) |
| C(10)-C(9)-C(11) | 111.85(19) |
| O(3)-C(9)-C(8) | 108.98(15) |
| C(10)-C(9)-C(8) | 111.47(17) |
| C(11)-C(9)-C(8) | 111.23(19) |
| C(9)-C(10)-H(10A) | 109.5 |
| C(9)-C(10)-H(10B) | 109.5 |
| H(10A)-C(10)-H(10B) | 109.5 |
| C(9)-C(10)-H(10C) | 109.5 |
| H(10A)-C(10)-H(10C) | 109.5 |
| H(10B)-C(10)-H(10C) | 109.5 |
| C(9)-C(11)-H(11A) | 109.5 |
| C(9)-C(11)-H(11B) | 109.5 |
| H(11A)-C(11)-H(11B) | 109.5 |
| C(9)-C(11)-H(11C) | 109.5 |
| H(11A)-C(11)-H(11C) | 109.5 |
| H(11B)-C(11)-H(11C) | 109.5 |

**Table S2.** Anisotropic displacement parameters (Å^2^x10^3^) for **4**. The anisotropic displacement factor exponent takes the form: -2π^2^[h^2^a*^2^U^11^ + ... + 2 h k a*b*U^12^].

|  | **U^11^** | **U^22^** | **U^33^** | **U^23^** | **U^13^** | **U^12^** |
| --- | --- | --- | --- | --- | --- | --- |
| O(4) | 43(1) | 51(1) | 35(1) | 9(1) | 9(1) | -17(1) |
| C(2) | 31(1) | 35(1) | 35(1) | 12(1) | 6(1) | 0(1) |
| O(3) | 46(1) | 47(1) | 38(1) | 5(1) | 6(1) | -18(1) |
| O(5) | 45(1) | 48(1) | 42(1) | 4(1) | 13(1) | -11(1) |
| O(2) | 52(1) | 70(1) | 39(1) | 11(1) | 14(1) | -9(1) |
| C(5) | 32(1) | 39(1) | 35(1) | 13(1) | 7(1) | -2(1) |
| O(1) | 55(1) | 56(1) | 32(1) | 2(1) | 10(1) | -13(1) |
| C(4) | 38(1) | 41(1) | 29(1) | 7(1) | 4(1) | -5(1) |
| O(6) | 48(1) | 55(1) | 47(1) | 1(1) | 15(1) | -21(1) |
| C(3) | 31(1) | 35(1) | 35(1) | 9(1) | 2(1) | -3(1) |
| C(1) | 37(1) | 37(1) | 31(1) | 7(1) | 6(1) | 2(1) |
| C(6) | 37(1) | 35(1) | 37(1) | 6(1) | 5(1) | -8(1) |
| C(8) | 29(1) | 47(1) | 45(1) | 17(1) | 7(1) | 0(1) |
| C(7) | 34(1) | 43(1) | 38(1) | 14(1) | 8(1) | 5(1) |
| C(12) | 38(1) | 43(1) | 37(1) | 8(1) | 6(1) | -10(1) |
| C(13) | 36(1) | 35(1) | 39(1) | 10(1) | 7(1) | -3(1) |
| C(9) | 34(1) | 38(1) | 50(1) | 12(1) | 11(1) | -6(1) |
| C(10) | 51(1) | 58(1) | 65(2) | -1(1) | 12(1) | -22(1) |
| C(11) | 49(1) | 47(1) | 105(2) | 28(1) | 23(1) | 11(1) |

**Table S3.** Analysis of the intermolecular contacts on the HS of **4**, according to Jelsch *et al*. (see reference in the main text). The first part of the table gives the surface contribution S*_X_* of each chemical type *X* to the HS. The second part shows the proportions of the actual contacts (C*_XY_*), and the third part indicates the enrichment ratios (E*_XY_*) of the various contact types. Reciprocal contacts X···Y and Y···X are merged. The E*_XY_* were not computed when the random contacts (R*_XY_*) were lower than 1%. E*_XY_* larger than unity indicate enriched contacts (in bold). The percentages of actual contacts were calculated using CrystalExplorer21.5.

| **Atoms** | **H** | **C** | | **O** |
| --- | --- | --- | --- | --- |
| *Surface (%)* | 65.3 | 9.8 | | 25.1 |
| *Contacts (%)* |  | |  |  |
| H | 38.5 |  | |  |
| C | 12.7 | 1.3 | |  |
| O | 40.8 | 4.3 | | 2.5 |
| *Enrichments* |  | |  |  |
| H | 0.9 |  | |  |
| C | **1.0** | - | |  |
| O | **1.2** | 0.9 | | 0.4 |

**Biophysical Investigation – Detailed MST assays**


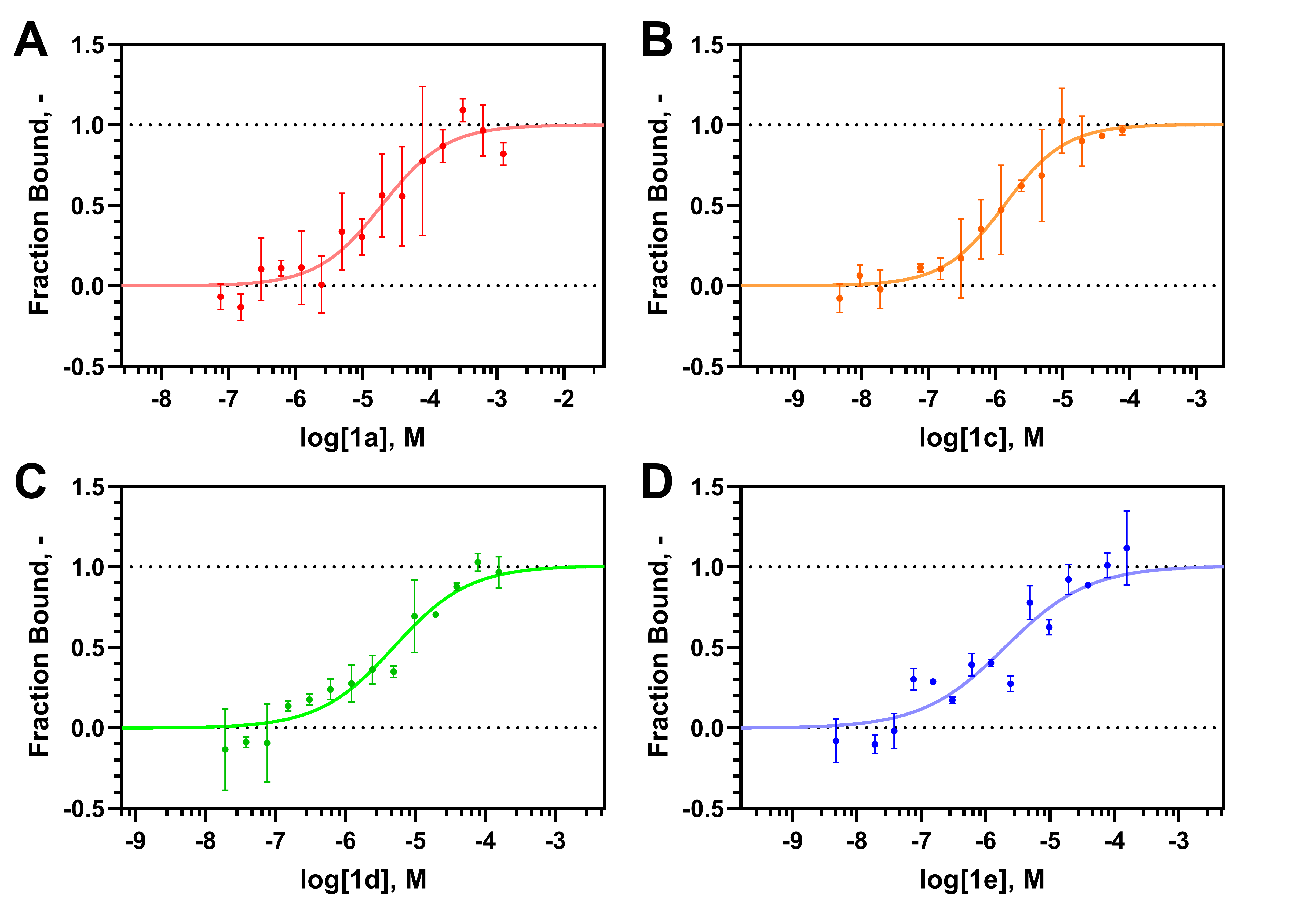


**Figure S27.** MST curves normalized by fraction bound acquired by recombinant WrbA incubated with scaling concentrations of compounds **1a** (A), **1c** (B), **1d** (C), and **1e** (D) using a Monolith NT.115 instrument. In the case of **1c** and **1e**, the two data points corresponding to concentrations of 38.4 nM and 9.6 nM, respectively, were excluded from the fitting as they were clear outliers.

**Table S4.** Summary of the MST experiments conducted with a fixed concentration of WrbA and scaling concentrations of compounds **1a**, **1c**, **1d**, and **1e**. Two independent experiments were performed to determine the K_d_ value.

| **Compound** | **[WrbA]** | **MST Power** | **Exc. Power** | **Temp.** | **[Ligand] Range** | **Time** | **RA** | **SNR** | **K_d_ (µM)** |
| --- | --- | --- | --- | --- | --- | --- | --- | --- | --- |
| **1a** | 50 nM | 60% | 60% | 25 °C | 1.25 mM - 76.3 nM | 5 s | 2.7 | 10.0 | 18.7 ± 6.3 |
| **1c** | 35 nM | 40% | 80% | 25 °C | 78.1 µM - 4.8 nM | 5 s | 2.6 | 7.5 | 1.4 ± 0.7 |
| **1d** | 35 nM | 40% | 80% | 25 °C | 156 µM - 19 nM | 2.5 s | 5.0 | 10.1 | 5.0 ± 1.8 |
| **1e** | 35 nM | 40% | 80% | 25 °C | 156 µM - 4.8 nM | 10 s | 5.4 | 5.6 | 2.1 ± 1.3 |

RA = Response Amplitude, SNR = Signal-to-Noise Ratio.

**MD simulations – RMSD graphs of compounds 1a-e**


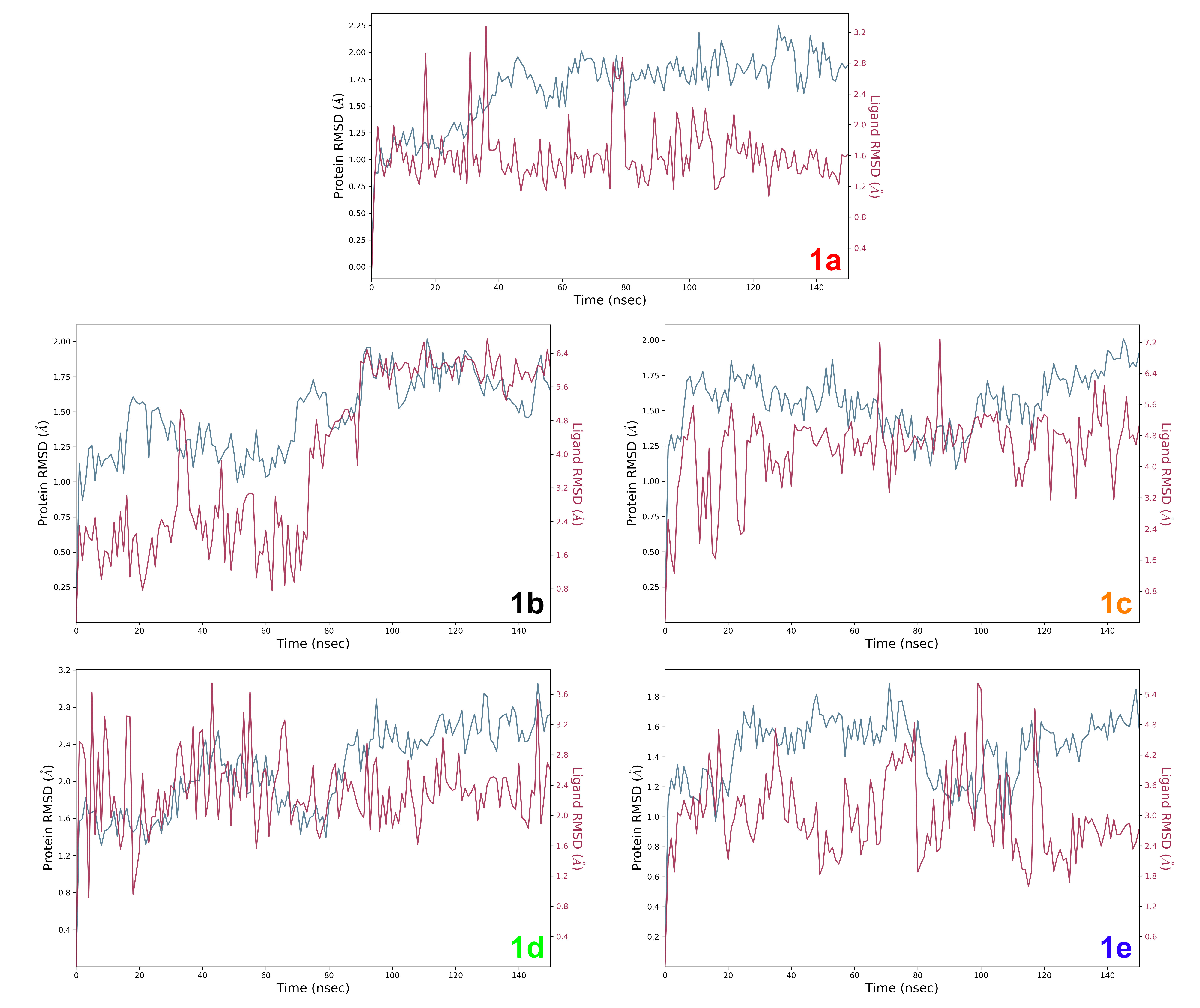


**Figure S28.** Cα atoms RMSD plots of the compounds **1a-e** over 150 ns of MD simulation time.

**Total Antioxidant Capacity (TAC) of 1a-e**

The Total Antioxidant Capacity (TAC) of **1a-e** was evaluated using the MAK334 Antioxidant Assay Kit, produced by Sigma-Aldrich (Merck KGaA, Darmstadt, German). The test detects antioxidants by measuring their capacity to reduce Cu²⁺ to Cu⁺, which then reacts with a dye reagent to form a colored complex. The color intensity at 570 nm is proportional to the TAC of the sample. Because the assay uses Trolox (6-hydroxy-2,5,7,8-tetramethylchroman-2-carboxylic acid), a water-soluble analog of vitamin E, as the standard, the TAC is expressed as concentration (μM) of Trolox equivalents. The experiment was performed according to the manufacturer’s instructions. Briefly, 250 μL of a 1 mM Trolox standard was prepared by combining 5 μL of standard with 245 μL of ultrapure water. The standard was further diluted with water to 600 and 300 μM. A blank was also prepared only with water. Then, 20 μL of standard, blank, and samples at the desired concentrations, *i.e.*, 500, 50, 5, 0.5 μM, were transferred to a clear, flat-bottom 96-well plate. 100 μL of reaction mix (previously prepared) was added to each well, and the plate was incubated for 10 min at room temperature after gentle mixing. The absorbance was read at 570 nM using an Agilent BioTek Synergy H1 Multimode Microplate Reader (Agilent Technologies, Inc., Santa Clara, CA, USA), and the TAC was calculated using the following equation (Eq. 1), based on the mean of two replicates:

$$TAC \left( \mu M \right)=\frac{{(A_{570})}_{sample}-{(A_{570})}_{blank}}{slope ({\mu M}^{-1})}\times n$$

where:

(A_570_)_sample_ = absorbance of the sample

(A_570_)_blank_ = absorbance of the medium blank

n = sample dilution factor

**Panel 1.** Top left: TAC (μM) values of **1a-e**, calculated according to Eq. 1. Top right: standard curve of Trolox, with the equation and the R^2^ value. Bottom left: photograph of the 96-well plate used in the experiment. Bottom right: distribution plan for the 96-well plate.

| \|  \| **TAC (μM)** \| \| \| \| \| --- \| --- \| --- \| --- \| --- \| \|  \| *500 μM* \| *50 μM* \| *5 μM* \| *0.5 μM* \| \| **1a** \| 0.588 \| 0.588 \| 0 \| 0 \| \| **1b** \| 18.824 \| 0.588 \| 0 \| 0 \| \| **1c** \| 5.882 \| 5.294 \| 0 \| 0 \| \| **1d** \| 14.706 \| 1.765 \| 0 \| 0 \| \| **1e** \| 30.588 \| 0 \| 0 \| 0 \| | 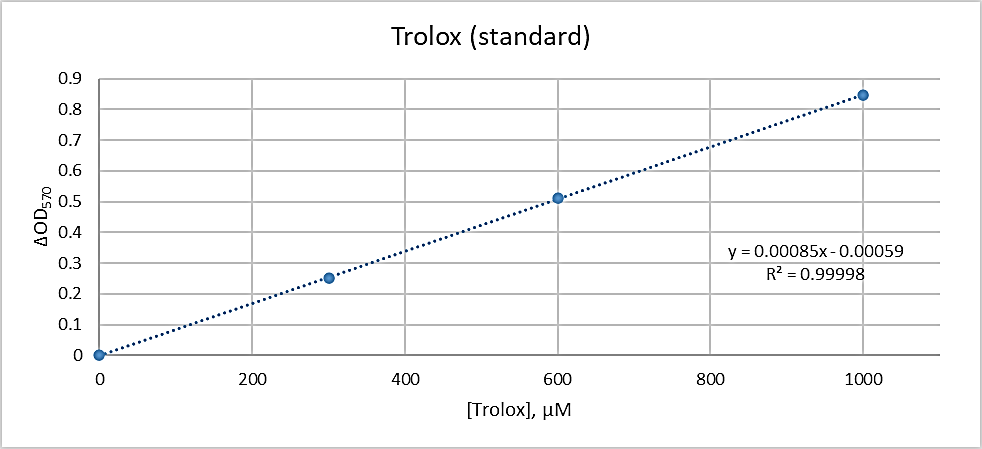 |
| --- | --- | --- | --- | --- | --- | --- | --- | --- | --- | --- | --- | --- | --- | --- | --- | --- | --- | --- | --- | --- | --- | --- | --- | --- | --- | --- | --- | --- | --- | --- | --- | --- | --- | --- | --- | --- |
| 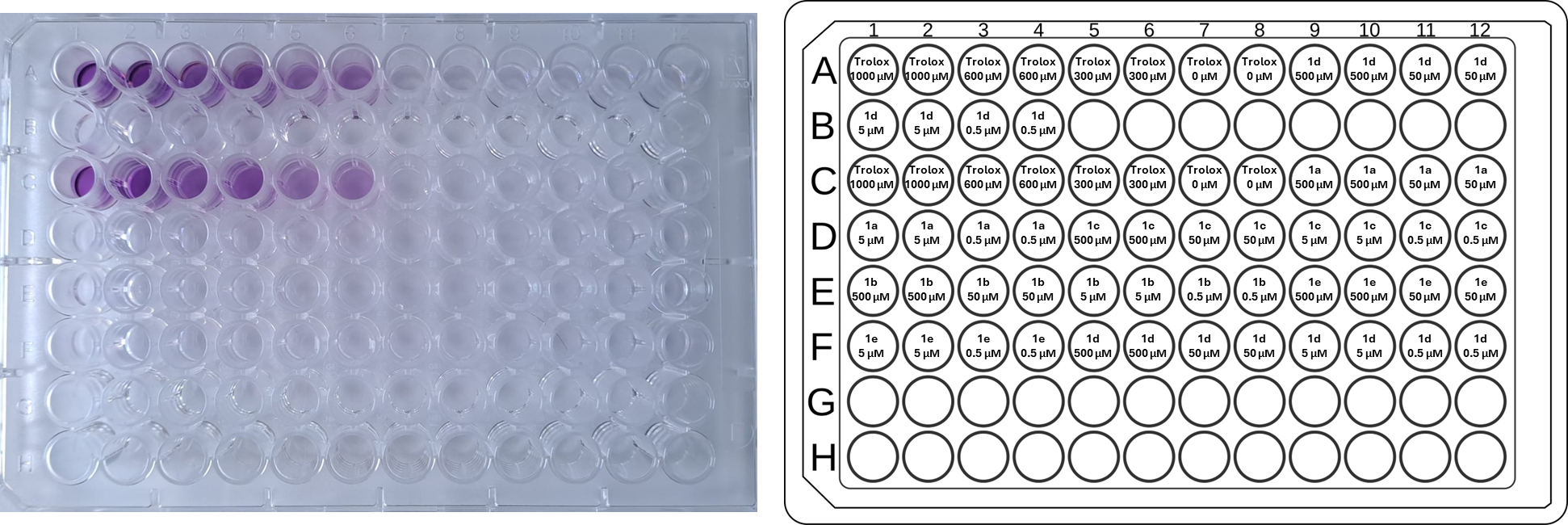 | |

**Predicted human toxicity of compounds 1a-e**

**Table S5.** *In silico* prediction of the human toxicity of compounds **1a-e**, performed using the ADMETLab server (<https://admet.scbdd.com/home/index/>; *Nucleic Acids Res.* **2024**, *528*(W1), W422–W431).

| **Compound** | **Property** | **Predicted value** | **Probability** |
| --- | --- | --- | --- |
| **1a** | hERG (hERG Blockers) | --- | 0.282 |
|  | H-HT (Human Hepatotoxicity) | - | 0.492 |
|  | AMES (Ames Mutagenicity) | --- | 0.286 |
|  | SkinSen (Skin sensitization) | --- | 0.185 |
|  | LD_50_ (LD_50_ of acute toxicity) | 2.438 -log mol/kg  (967.565 mg/kg) | / |
|  | DILI (Drug-Induced Liver Injury) | - | 0.440 |
|  | FDAMDD (Maximum Recommended Daily Dose) | + | 0.616 |
| **1b** | hERG (hERG Blockers) | ++ | 0.708 |
|  | H-HT (Human Hepatotoxicity) | ++ | 0.700 |
|  | AMES (Ames Mutagenicity) | - | 0.334 |
|  | SkinSen (Skin sensitization) | --- | 0.189 |
|  | LD_50_ (LD_50_ of acute toxicity) | 2.616 -log mol/kg  (1085.878 mg/kg) | / |
|  | DILI (Drug-Induced Liver Injury) | - | 0.480 |
|  | FDAMDD (Maximum Recommended Daily Dose) | + | 0.548 |
| **1c** | hERG (hERG Blockers) | - | 0.486 |
|  | H-HT (Human Hepatotoxicity) | ++ | 0.742 |
|  | AMES (Ames Mutagenicity) | - | 0.334 |
|  | SkinSen (Skin sensitization) | --- | 0.189 |
|  | LD_50_ (LD_50_ of acute toxicity) | 2.666 -log mol/kg  (859.773 mg/kg) | / |
|  | DILI (Drug-Induced Liver Injury) | - | 0.480 |
|  | FDAMDD (Maximum Recommended Daily Dose) | + | 0.532 |
| **1d** | hERG (hERG Blockers) | - | 0.390 |
|  | H-HT (Human Hepatotoxicity) | +++ | 0.904 |
|  | AMES (Ames Mutagenicity) | --- | 0.250 |
|  | SkinSen (Skin sensitization) | --- | 0.174 |
|  | LD_50_ (LD_50_ of acute toxicity) | 2.545 -log mol/kg  (1111.427 mg/kg) | / |
|  | DILI (Drug-Induced Liver Injury) | - | 0.456 |
|  | FDAMDD (Maximum Recommended Daily Dose) | + | 0.538 |
| **1e** | hERG (hERG Blockers) | + | 0.538 |
|  | H-HT (Human Hepatotoxicity) | + | 0.656 |
|  | AMES (Ames Mutagenicity) | - | 0.354 |
|  | SkinSen (Skin sensitization) | --- | 0.175 |
|  | LD_50_ (LD_50_ of acute toxicity) | 2.735 -log mol/kg  (746.341 mg/kg) | / |
|  | DILI (Drug-Induced Liver Injury) | - | 0.450 |
|  | FDAMDD (Maximum Recommended Daily Dose) | + | 0.548 |
| ***Meaning and preferred values***   - *hERG (hERG Blockers):* molecules with IC_50_ < 40 μM are regarded as blockers [*Trends Pharmacol. Sci.* **2005**, *26*(3), 119-124]. Features that may lead to hERG blocking: a basic amine (positively ionizable, pK_a_ >7.3); hydrophobic/lipophilic substructure(s) (ClogP > 3.7); absence of negatively ionizable groups or oxygen H-bond acceptors [ISBN: 978-0-1236-9520-8. pp. 213; *Mol. Pharm.* **2016**, *13*(8), 2855–2866]. - *H-HT (Human Hepatotoxicity):* H-HT positive(+) & negative(-) classification criteria are based on the reference [*Chem. Res. Toxicol.* **2016**, *29*(5), 757-767]. - *AMES (Ames Mutagenicity):* Ames positive(+) compounds significantly induce revertant colony growth in at least one out of usually five strains; otherwise, compounds are classified as negative(-) [*J. Chem. Inf. Model.* **2012**, *52*(11), 2840-2847]. - *SkinSen (Skin sensitization):* prediction is based on data from the (reduced) Local Lymph Node Assay ((r)LLNA), which classifies compounds as either sensitizers or non-sensitizers [*Toxicol. Appl. Pharm.* **2015**, *284*(2), 262-272]. - *LD_50_ (LD_50_ of acute toxicity):* median lethal dose (LD_50_) usually represents the acute toxicity of chemicals. It refers to the dose required to cause death in 50% of a test animal population within a specified time frame [*Chem. Res. Toxicol.* **2009**, *22*(12), 1913–1921]. High toxicity: 1~50 mg/kg; toxicity: 51~500 mg/kg; low toxicity: 501~5000 mg/kg (suggestion: > 500 mg/kg) [*J. Cheminformatics* **2016**, *8*(1), 6]. - *DILI (Drug-Induced Liver Injury):* DILI positive(+) & negative(-) classification criteria are based on the reference [*J. Chem. Inf. Model.* **2015**, *55*(10). 2085-2093]. - *FDAMDD (Maximum Recommended Daily Dose):* FDAMDD positive(+) & negative(-) classification criteria are based on the reference [*Chemometr. Intell. Lab.* **2015**, *146*, 494-502]. | | | |
